# Identification and quantification of glucose degradation products in heat-sterilized glucose solutions for parenteral use by thin-layer chromatography

**DOI:** 10.1371/journal.pone.0253811

**Published:** 2021-07-02

**Authors:** Sarah Leitzen, Matthias Vogel, Anette Engels, Thomas Zapf, Martin Brandl

**Affiliations:** 1 Department of Physics, Chemistry and Pharmacy, University of Southern Denmark, Odense, Denmark; 2 Federal Institute for Drugs and Medical Devices, Bonn, Germany; Cairo University, EGYPT

## Abstract

During heat sterilization of glucose solutions, a variety of glucose degradation products (GDPs) may be formed. GDPs can cause cytotoxic effects after parenteral administration of these solutions. The aim of the current study therefore was to develop a simple and quick high-performance thin-layer chromatography (HPTLC) method by which the major GDPs can be identified and (summarily) quantified in glucose solutions for parenteral administration. All GDPs were derivatized with o-phenylenediamine (OPD). The resulting GDP derivatives (quinoxalines) were applied to an HPTLC plate. After 20 minutes of chamber saturation with the solvent, the HPTLC plate was developed in a mixture of 1,4-dioxane-toluene-glacial acetic acid (49:49:2, v/v/v), treated with thymol-sulfuric acid spray reagent, and heated at 130°C for 10 minutes. Finally, the GDPs were quantified by using a TLC scanner. For validation, the identities of the quinoxaline derivatives were confirmed by liquid chromatography-tandem mass spectrometry (LC-MS/MS). Glyoxal (GO)/methylglyoxal (MGO) and 3-deoxyglucosone (3-DG)/3-deoxygalactosone (3-DGal) could be identified and quantified in pairs, glucosone (2-KDG), 5-hydroxymethylfurfural (5-HMF), and 3,4-dideoxyglucosone-3-ene (3,4-DGE) each individually. For 2-KDG, the linearity of the method was demonstrated in the range of 1–50 μg/mL, for 5-HMF and 3,4-DGE 1–75 μg/mL, for GO/MGO 2–150 μg/mL, and for 3-DG/3-DGal 10–150 μg/mL. All GDPs achieved a limit of detection (LOD) of 2 μg/mL or less and a limit of quantification (LOQ) of 10 μg/mL or less. R^2^ was 0.982 for 3.4-DGE, 0.997 for 5-HMF, and 0.999 for 2-KDG, 3-DG/3-DGal, and GO/MGO. The intraday precision was between 0.4 and 14.2% and the accuracy, reported as % recovery, between 86.4 and 112.7%. The proposed HPTLC method appears to be an inexpensive, fast, and sufficiently sensitive approach for routine quantitative analysis of GDPs in heat-sterilized glucose solutions.

## 1. Introduction

Sterile glucose solutions are commonly used as reconstitution solvents or diluents for injectable drugs and also for peritoneal dialysis solutions [1]. In Germany, regulatory requirements for the different strengths of glucose solutions used for parenteral administration are regulated and published as standard marketing authorizations.

Indications for the use of glucose solutions range from the treatment of carbohydrate deficiencies and fluid losses, carrier solutions for compatible drugs and electrolyte concentrates, additions of free water, energy supplies, hypoglycemic states, and high-calorie intake when fluids are restricted to carbohydrate components in parenteral nutrition [2]. In addition, glucose solutions for infusions are widely used as alternatives to isotonic 0.9% saline solutions to reconstitute powders or dilute concentrates for parenteral use, especially when the respective drugs are incompatible with saline solutions [3, 4].

Thus, sterility of parenteral glucose solutions represents a crucial safety requirement [5]. To guarantee the sterility of glucose solutions, steam sterilization, i.e., exposure to saturated steam under pressure (autoclaving), is the method of choice. Terminal sterilization is compulsory unless the parenteral product or its primary packaging does not tolerate this technique [5, 6]. For the standard autoclaving process at 121°C, a sterility assurance level of 10^−6^ is typically achieved; at the same time glucose degradation products (GDPs) may be formed during such heat treatment, negatively affecting the quality of glucose solutions [7–9]. To date, several monocarbonyl and dicarbonyl degradation products have been identified [10–12]. Most GDPs can be deduced from glucose after oxidative and dehydration processes caused by the effect of heat.

Furthermore, a keto-enol tautomerism can be observed in aqueous solutions of glucose. Upon dehydration and tautomerization deoxyglucosones can be formed, which then might degrade further to either 5-HMF by cyclization or to short-chain products such as MGO and GO by retro aldol cleavage reactions [13].

Physiologically, GDPs modulate cellular functions by triggering inflammation, which can cause structural and functional changes in membranes and, thus, promote fibrosis, angiogenesis, and loss of ultrafiltration capacity (peritoneal dialysis) [10]. Additionally, binding to proteins can impair the enzymatic activity and thus lead to reduced cell viability. Furthermore, reactive amino acid side chains of proteins may react with GDPs and form so-called AGEs (advanced glycation end products) [1, 7], which are being investigated in the context of Alzheimer’s disease [14], cardiovascular disease, and stroke [15, 16]. Concerning toxic effects after peritoneal administration, Nakayama *et al* found that AGEs could also accumulate in vessels outside the peritoneum. It can be assumed that AGEs are deposited on the vessel walls during parenteral administration of a glucose solution [17]. To date, however, only limited information is available concerning the amount of GDPs in glucose solutions used for parenteral administration [1, 7, 18–20]. This underscores the need to more closely examine the GDPs in glucose solutions for parenteral administration [17] and supports the goal of reducing the overall amount of GDPs generated during the sterilization process to a minimum.

Sensitive and reliable (ultra) high-performance liquid chromatography with ultraviolet/ diode array/ tandem mass spectrometry detection ((U)HPLC-UV/ DAD/ MS/MS) methods to identify and quantify GDPs in glucose solutions have already been described in the literature [10]. However, these methods require the use of advanced analytical equipment, which is not readily available at many nonindustrial sites where glucose infusions are produced, in particular hospital pharmacies.

The objective of this work, therefore, was to establish and validate a fast, reliable, and sufficiently sensitive high-performance thin-layer chromatography (HPTLC) method for the qualitative and quantitative analysis of the seven most common GDPs, including the α-dicarbonylic compounds glyoxal (GO), methylglyoxal (MGO), glucosone (2-KDG), 3-deoxyglucosone (3-DG), 3-deoxygalactosone (3-DGal), 3,4-dideoxyglucosone-3-ene (3,4-DGE), and the impurity 5-HMF. Ideally the method should be suitable for later use in routine analysis under standardized conditions. While limit values of up to 22 mg/L (5%), 44 mg/L (10%), 88 mg/L (20%), 175 mg/L (40%), and 220 mg/L (50%) of 5-HMF are allowed for 5–50% glucose solutions according to standard marketing authorizations in Germany, no limit values have been published for α-DCs either in pharmacopoeias or by regulatory authorities to the best of our knowledge.

Following an approach suggested by Mittelmaier *et al*. (2011) [10] in the context of HPLC-based analysis, GDPs were derivatized with o-phenylenediamine (OPD) in our study, resulting in the corresponding quinoxaline products, and the suitability of the derivatization protocol was verified (see paragraph 2.2.2). Using HPTLC, the derivatized degradation products were developed in a mixture of 1,4-dioxane-toluene-glacial acetic acid (49:49:2, v/v/v), sprayed with thymol-sulfuric acid reagent, then heated at 130°C for 10 minutes, and subsequently identified by UV determination at 366 nm. For validation purposes, the identities of the derivatized spots were verified by LC-MS/MS.

## 2. Materials and methods

### 2.1 Reagents and chemicals

For all experiments, freshly prepared ultrapure water was taken from the Sartorius arium® pro UV water-treatment system (Sartorius AG, Göttingen, Germany). All chemicals were of analytical grade, unless noted otherwise. Acetonitrile, OPD, 2,3-dimethylquinoxaline, MGO, GO, 2-KDG, 3-DG, 5-HMF, D-(+)-glucose, methanol, and ammonium acetate (for mass spectrometry) were purchased from Sigma (Sigma-Aldrich Chemie GmbH, Steinheim, Germany). Acetic acid (glacial) 100%, toluene, sulfuric acid 95–98%, and ethanol 96% were obtained from Merck (Merck KGaA, Darmstadt, Germany). Furthermore, 1,4-dioxane and thymol were purchased from Roth (Carl Roth GmbH & Co. KG, Karlsruhe, Germany). The 3-DGal was obtained from Cayman (Cayman Chemical Company, Ann Arbor, Michigan, USA), and 3,4-DGE was purchased from Carbosynth (Carbosynth Ltd, Compton, Berkshire, UK).

Thymol-sulfuric acid reagent was freshly prepared according to the monograph "Glucose" from the Ph. Eur., section C "Thin Layer Chromatography", subitem “Detection” [21]:

0.5 g of thymol were solved in a mixture of 5 mL of sulfuric acid and 95 mL of ethanol 96% (v/v). No cooling was required for this step.

The aqueous phase during LC-MS/MS measurement was a 5 mM ammonium acetate buffer solution adjusted to pH = 3.5 using 0.1% (v/v) acetic acid. It was freshly prepared according to Thomas *et al* (2013) [22].

### 2.2 Experimental

#### 2.2.1 Derivatization of GDPs with OPD

**Fig 1:**
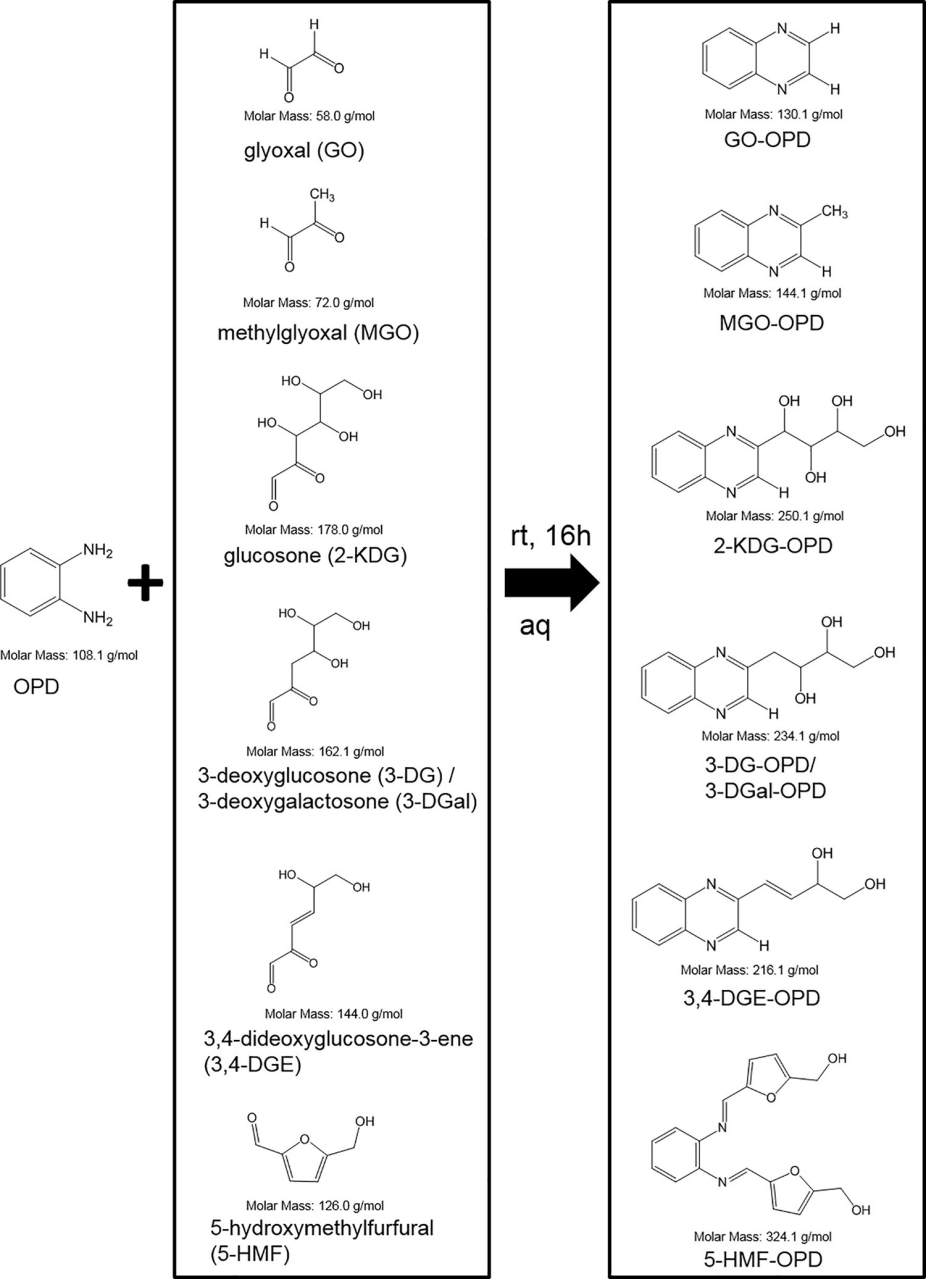
Scheme of the derivatized GDPs upon addition of OPD after 16 h of derivatization time at room temperature. The reaction of OPD with glucose and 5-HMF can result in stoichiometric ratios of either 1:1 or 1:2, resulting in single- or double-substituted OPD molecules.

According to Mittelmaier *et al*. (2011) the derivatization method was performed simultaneously for all GDPs [10]. For each degradation product (GO, MGO, 2-KDG, 3-DG, 3-DGal, 3,4-DGE, and 5-HMF) a solution was produced in a concentration of 0.5 mg/mL in freshly prepared ultrapure water, and a mixture containing all seven GDPs was produced at a concentration level of 0.5 mg/mL. All solutions also contained 50 mg/mL glucose and 7.5 mg/mL OPD. They were left to stand in the dark for 16 hours at ambient temperature. An overview of all expected [1, 7, 10, 13, 23, 24] derivatized GDPs can be found in Fig 1.

#### 2.2.2 Verification of the selected derivatization procedure with OPD to quantify all seven GDPs

Four different solutions were prepared in amber vials, each containing different concentrations of OPD and glucose matrix. Solutions 1–3 contained a GDP mix, with each GDP present in the mix at a concentration of 10 μg/mL. In addition, the solutions contained 50 mg/mL glucose and 5 μg/mL internal standard. Solution 1 contained 0.60 mg/mL OPD, solution 2 0.75 mg/mL OPD, and solution 3 0.90 mg/mL OPD. Solution 4 corresponded to solution 2 without glucose matrix. The LC-MS/MS analysis of solutions 1–4 was carried out hourly over a period of 24 h at room temperature. The peak area of the derivatized GDP was related to the peak area of the internal standard 2,3-dimethylquinoxaline and plotted against the derivatization time.

#### 2.2.3 Qualitative HPTLC analysis

An HPTLC plate (Silica gel 60 F 254 glass plate 20x20 cm, Merck, Darmstadt) was prepared with all derivatized GDP standards and the described mixture, as reported in in section 2.2.1. A volume of 10 μL of each sample/standard was applied as a band with a width of 10 mm and at a distance of 9 mm between the bands. A CAMAG Automatic TLC Sampler 4 (CAMAG AG, Muttenz, Switzerland) was used to apply the derivatized samples. Afterwards, the bands were air-dried under the laboratory fume hood at room temperature (ambient air) for 5 minutes. The eluent consisted of 1,4-dioxane-toluene-glacial acetic acid at a ratio of 49:49:2, (v/v/v). Various alternative mobile phases were tried as part of the method development, which is described in more detail in the results and discussion sections. Each trough of a double-trough chamber was filled with 25 mL of the eluent. One trough of the TLC chamber was lined with filter paper. The chamber was kept at room temperature for 20 minutes to achieve equilibration of the vapor atmosphere. The HPTLC plate was developed over a migration distance of approx. 16 cm and took about 75 minutes, with a subsequent air-drying period of 10 minutes (ambient air under the laboratory fume hood at room temperature). Afterwards, the spots on the plate were detected in daylight (R White, T White, RT White), 254 nm and 366 nm by CAMAG TLC Visualizer 2. While establishing the method, two identical plates were run simultaneously, where one of the two plates was treated with 2 mL of a solution of 0.5 g thymol in a mixture of 5 mL sulfuric acid and 95 mL ethanol 96% (v/v) using the CAMAG Derivatizer, yellow nozzle, level 4. Hereafter, the plate was heated for 10 minutes at 130°C on the CAMAG TLC Plate Heater III and again detected in daylight (R White, T White, RT White), 254 nm and 366 nm. The software used throughout the method was visionCATS, version 2.3.

The purpose of the duplicate plate (plate without 2^nd^ derivatization step) was to identify the compounds in the bands by LC-MS/MS in order to assign the respective derivatives to the spots. In preparation for the LC-MS/MS analysis, the Rf values of the other derivatized plate were noted and spots scraped off of the duplicate plate at the expected height. Afterwards, the OPD-GDPs were extracted individually from the spots: the silica gel was extracted three times, using 200 μL methanol each time, and centrifuged for approx. 2 min at 16,000 rpm/ 21,130 rcf (Eppendorf Centrifuge 5424/5424R, Rotor FA-45-24-11, r_max_ = 8.4 cm, Hamburg, Germany). The supernatants were evaporated with nitrogen for 1.5 h at 40.0°C (VLM metal block thermostat type EC-1V-130). The residues were finally reconstituted in 200 μL of a 5 mM ammonium acetate buffer solution adjusted to pH = 3.5 using 0.1% (v/v) acetic acid and the identity of the compounds verified via LC-MS/MS (see paragraph 2.2.4).

#### 2.2.4 Spot identification via liquid chromatography–tandem mass spectrometry

As the starting point for the LC-MS/MS method, we optimized the process reported in Mittelmaier *et al*. [10] for this study. A qualitative analysis and structure elucidation was performed via HPLC-MS/MS. The respective LC-MS/MS parameters and ion transitions are shown in S2 Table. Liquid chromatography was performed on a Shimadzu Nexera UFLC high-performance ultra-fast liquid chromatograph equipped with an analytical C8 column (Accucore C8, 50 mm× 3 mm, 2.6-μm particle size, Thermo Scientific, Schwerte, Germany). The UHPLC system (degasser, binary pump, autosampler, and column oven) was coupled to a SCIEX QTrap6500 triple quadrupole mass spectrometer (Sciex, Darmstadt, Hessen, Germany) and operated under positive electrospray ionization (ESI) conditions with a needle voltage of 5500 V at 450°C and nitrogen as drying gas.

The collision energies were 40 eV for the MGO and GO derivatives and 20 eV for 2-KDG, 3-DG, 3-DGal, 3,4-DGE, and 25 eV for 5-HMF derivatives, respectively. Mobile phase A consisted of a 5 mM ammonium acetate buffer solution adjusted to pH 3.5 using 0.1% (v/v) acetic acid, and mobile phase B consisted of acetonitrile. The total flow rate was 0.5 mL/min. The gradient started at 99% solvent A, remained isocratic for 0.2 min, and increased to 30% B within 10 minutes. Afterwards, within 3 minutes, B increased to 100% and remained at 100% B for 1 minute. The column was re-equilibrated from 14.01 min to 16.00 min at 99% A. The overall run time was 16 min. The injection volume was 5 μL. System control, data acquisition, and processing were performed using Analyst 1.6.2 software. Product ion mass spectra for all GDPs can be found in the (S8–S15 Figs).

#### 2.2.5 Purity of 3,4-DGE

In order to investigate the purity of 3,4-DGE, a solution of 50 μg/mL was prepared in methanol. The solutions were measured via LC-MS/MS using an EMS full scan. The setting for the LC-MS/MS was taken from section 2.2.4.

#### 2.2.6 Stability of GDPs in the chosen eluent

To evaluate the stability of the GDPs under acidic conditions, four solutions were prepared and the ratio of the peak area of each GDP/ the peak area of the internal standard (= 2,3-dimethylquinoxaline) was computed using the LC-MS/MS method described in section 2.2.4. Solutions 1 and 3 each contained all GDPs at a concentration of 10 μg/mL, 0.75 mg/mL OPD, 5 μg/mL 2,3-dimethylquinoxaline, and 50 mg/mL glucose. Solutions 2 and 4 had the same composition as solutions 1 and 3, but without added glucose. All four solutions were left to stand in the dark at room temperature for 16 hours.

Then, 100 μL of solutions 1 and 2 were each made with 900 μL of water, and 100 μL of solutions 3 and 4 were each made with 900 μL of the eluent described in section 2.2.3 and incubated for 75 minutes. The solutions were then diluted 1:10 with water and measured by LC-MS/MS.

#### 2.2.7 Quantitative analysis, method validation, and analysis of a finished medicinal product

A calibration series containing all seven GDPs at concentration levels of 75 μg/mL, 50 μg/mL, 25 μg/mL, 10 μg/mL, 5 μg/mL, and 1 μg/mL was prepared. All solutions of the quantitative approach contained 50 mg/mL glucose and 0.75 mg/mL OPD.

A mixture of all GDPs was also prepared, containing concentrations at the levels (Table 1) that are expected to occur in a 5% glucose solution. This artificial mix was intended to simulate expected concentrations of GDPs in an autoclaved 5% glucose solution. The concentrations were based on averages from the references compiled in Table 1. The mix also contained glucose in a concentration of 50 mg/mL and OPD in a concentration of 0.75 mg/mL. After a 16-h reaction time at room temperature, the samples were analyzed by HPTLC as described in section 2.2.3. After staining with thymol-sulfuric acid, a scan step with the TLC scanner was performed. To finally determine the optimal substance-specific measuring wavelengths, UV-VIS scans of the substance zones were recorded in 20-nm steps in the wavelength range 190–800 nm. In this way, the optimal wavelength for each substance was analyzed. The objective was to achieve optimal separation of the substances and, at the same time, the highest signal strengths at the corresponding wavelengths. As a result, the HPTLC plate was scanned in both absorption and fluorescence mode at the wavelengths 330 nm, 366 nm, 370 nm, and 420 nm with different sharp-cut filters (K400, K540). Chromatograms were recorded from the calibration solutions of the GDPs at the optimal wavelengths that had been determined and calibration lines were generated from them. The final regression was calculated (Table 5).

**Table 1:** Averaged levels of expected GDPs in standard autoclaved 5% glucose solutions in reference to published data [1, 7, 26].

Analyte	Expected concentrations [μg/mL]
GO	1.0
MGO	1.0
2-KDG	7.0
3-DG	45.0
3-DGal	25.0
3,4-DGE	5.0
5-HMF	5.0

These contents served as reference values for the validation.

To validate the method, all parameters listed in the ICH Q2 (R1) guideline [25] were performed: Accuracy (reported as percent recovery), precision, linearity, range, limit of detection (LOD), and limit of quantitation (LOQ) were determined.

In order to calculate the accuracy (reported as % recovery), an unheated 5% glucose solution fluid was spiked with 5, 25, and 50 μg/mL of each GDP and 0.75 mg/mL OPD (i.e. 3 concentrations/3 replicates). These samples and an unspiked fluid were analyzed as described in section 2.2.3 after 16 h of derivatization The mean recovery of three experiments for each concentration level was determined and expressed as: (GDP concentration-GDP concentration of the unspiked sample)/ added GDP concentration×100% (Table 7).

Precision was expressed as standard deviation and coefficients of variation (% RSD). Nine determinations covering the specified range for the procedure (3 concentrations/3 replicates each) were made. The mean value, the standard deviation, and the precision were calculated (Table 6).

The LOD was determined based on visual evaluation as a ratio of 3:1 against the background noise and LOQ was determined based on visual evaluation as a ratio of 10:1 against the background noise [25].

A six-point-calibration curve in order to determine linearity was prepared in three replicates (1–75 μg/mL each GDP in water as well as 5% glucose and 0.75 mg/mL OPD). The calibration curve was obtained by plotting the peak areas or height of the derivatized GDPs (ordinate) against the concentration of the derivatized GDPs (abscissa).

Linear regression analysis was used to assess the linearity of the calibration curve.

The statistical formulas used here as a basis for calculation can be found in the supplementary information (supplement, pp. 33–34, lines 247–289).

Subsequently, the concentrations of the degradation products GO/MGO, 2-KDG, 3-DG/3-DGal, 3,4-DGE, and 5-HMF were determined in a 5% glucose finished drug solution. For this purpose, calibration solutions, as described in part 2.2.7, were used at concentrations 1–75 μg/mL, which contained 50 mg/mL glucose and 0.75 mg/mL OPD. Here, 1 mL of the finished drug also contained 0.75 mg/mL OPD. The solutions were left to stand in the dark at room temperature for 16 hours and then analyzed by HPTLC as described in section 2.2.3.

The selected concentration limits of the single GDPs in autoclaved 5% glucose solutions from Table 1 were averaged from previously published data [1, 7, 26] and served as reference limits for the future optimization of the autoclaving process.

## 3. Results and discussion

The purpose of this study was to develop and evaluate an HPTLC-based method to identify and quantify major GDPs in the form of α-DCs, in particular GO, MGO, 2-KDG, 3-DG, 3-DGal, and 3,4-DGE as well as the aldehyde 5-HMF.

### 3.1 Derivatization of GDPs with OPD

In the literature, complete separation of GDPs upon prior derivatization with OPD has been described for various (U)HPLC methods [10, 24, 27, 28]. For this reason, the derivatization reagent OPD was chosen for this HPTLC method, too.

A prerequisite for reliably quantifying GDPs is to ensure that the analytes have been completely derivatized and that no new degradation products were formed or existing degradation products volatilized during the derivatization process. This was shown by Mittelmaier *et al*. [10]. Furthermore, because of the formation of the quinoxaline structures, reactions for alpha-dicarbonyls were favored over monocarbonyls such as 5-HMF and glucose. The glucose, as a highly polar molecule, did not react with OPD, or only to a lesser degree, and maintained its hydrophilic character without forming the quinoxaline system. Therefore, retention of glucose was observed at the starting line of the HPTLC plate (S6 Fig).

The reaction of α-dicarbonyl GDPs with OPD led to the formation of much more lipophilic substances (quinoxalines). Therefore, the substances could be separated by normal-phase (NP) HPTLC. The fact that a quinoxaline system with a highly conjugated π-electron system was formed beneficially impacted detection via UV/Vis spectroscopy. Thereby, the of OPD concentration was carefully adapted to avoid undesired signal overlap.

### 3.2 Evaluation of the derivatization procedure

All relevant graphs can be found in the (S16–S21 Figs).

An evaluation of the derivatization process was carried out in parallel for all 7 GDPs.

The solutions to be tested contained a mix of 10 μg/mL of all GDPs, as well as 50 μg/mL glucose, 5 μg/mL internal standard, and OPD. The OPD concentration was varied in each solution (0.60, 0.75, and 0.90 mg/mL) in order to compare the derivatization effect at different OPD concentrations. In addition, the effect of the glucose matrix was investigated by omitting the addition of glucose to the 4^th^ solution.

It could be observed that an OPD concentration of 0.75 mg/mL, as was used in the quantification method, is well suited to completely derivatize the GDPs. Solution 4, spiked without glucose for comparison but which contained both 0.75 mg/mL OPD and the GDPs in concentrations of 10 μg/mL, shows a derivatization behavior identical to that of solution 2, to which 50 mg/mL glucose was added. This means that glucose itself does not react with OPD, or only very slightly, and the selected concentration of OPD of 0.75 mg/mL is sufficient to convert all GDPs. In addition, this experiment was used to test which derivatization time is adequate for all 7 GDPs. It could be shown that a plateau was reached for all GDPs after 16 h, except for 5-HMF (without matrix) and likewise for 2-KDG (with and without matrix) (see S16–S21 Figs). This was also described by Mittelmaier [29]. A higher concentration of OPD would probably help to reach a plateau faster. However, the HPTLC method limits the use of too-large amounts of OPD, as otherwise a very broad spot of OPD would occur here, overlapping the surrounding substances 3,4-DGE and 5-HMF (all Rf values are between 0.3 and 0.4). The chosen derivatization time of 16 h and the concentration of 0.75 mg/mL thus appear to represent a good compromise to be able to identify and quantify all 7 GDPs simultaneously in the presence of a considerable excess of glucose matrix. Moreover, both the test solution and the calibration solutions were treated in the same way, rendering the results for determining the concentration reproducible.

### 3.3 Eluent development

The analytes investigated here represent molecules with very similar structures. Glucose was present in a significant excess compared to the amounts of degradation products and should not be eluted. With the aim to separate the derivatized GDPs from each other and to identify them clearly, a suitable eluent should be developed. Interactions between the stationary phase, the mobile phase, and the sample were theoretically estimated using Stahl’s triangle [30]. In addition, elution power and miscibility of the solvents were taken into account while developing the method. For the derivatized GDPs described above, no HPTLC eluent for straight-phase separation has been reported in the literature so far.

First, the HPTLC steps were performed by adapting the recommended eluent from the European Pharmacopoeia for determining anhydrous glucose [21]. Here, the mobile phase consisted of water-methanol-glacial acetic acid-dichloroethane (10:15:25:50, v/v/v/v). After the plates were developed, they were sprayed with thymol-sulfuric acid reagent, as described in section 3.4 “Advantage of colorimetric determination with thymol-sulfuric acid reagent for qualitative TLC analysis”. For this eluent composition the Rf values of the derivatized GDPs were generally quite good (between 0.3–0.76), but glucose was also migrating and streaking (Rf = 0.3–0.4). GO and MGO were not separated at Rf = 0.76 and 2-KDG migrated into the OPD band (Rf = 0.54). Due to the unsatisfactory separation, 3,4-DGE, 3-DG, 3-DGal, and 5-HMF were not investigated here. Presumably, however, the latter four substances would all be in the Rf range 0.6–0.7 and would only be poorly separated from each other because they have similar migration distances.

Since the derivatized GDPs were significantly more apolar than the glucose itself, the solvent was modified using Stahl’s triangle [30] and a more nonpolar solvent was chosen.

A differentiated description up to the development of the final eluent can be found in the (S1 Table and S1–S5 Figs). All plates shown in the supplement were also sprayed with thymol-sulfuric acid reagent after they were developed.

**Fig 2:**
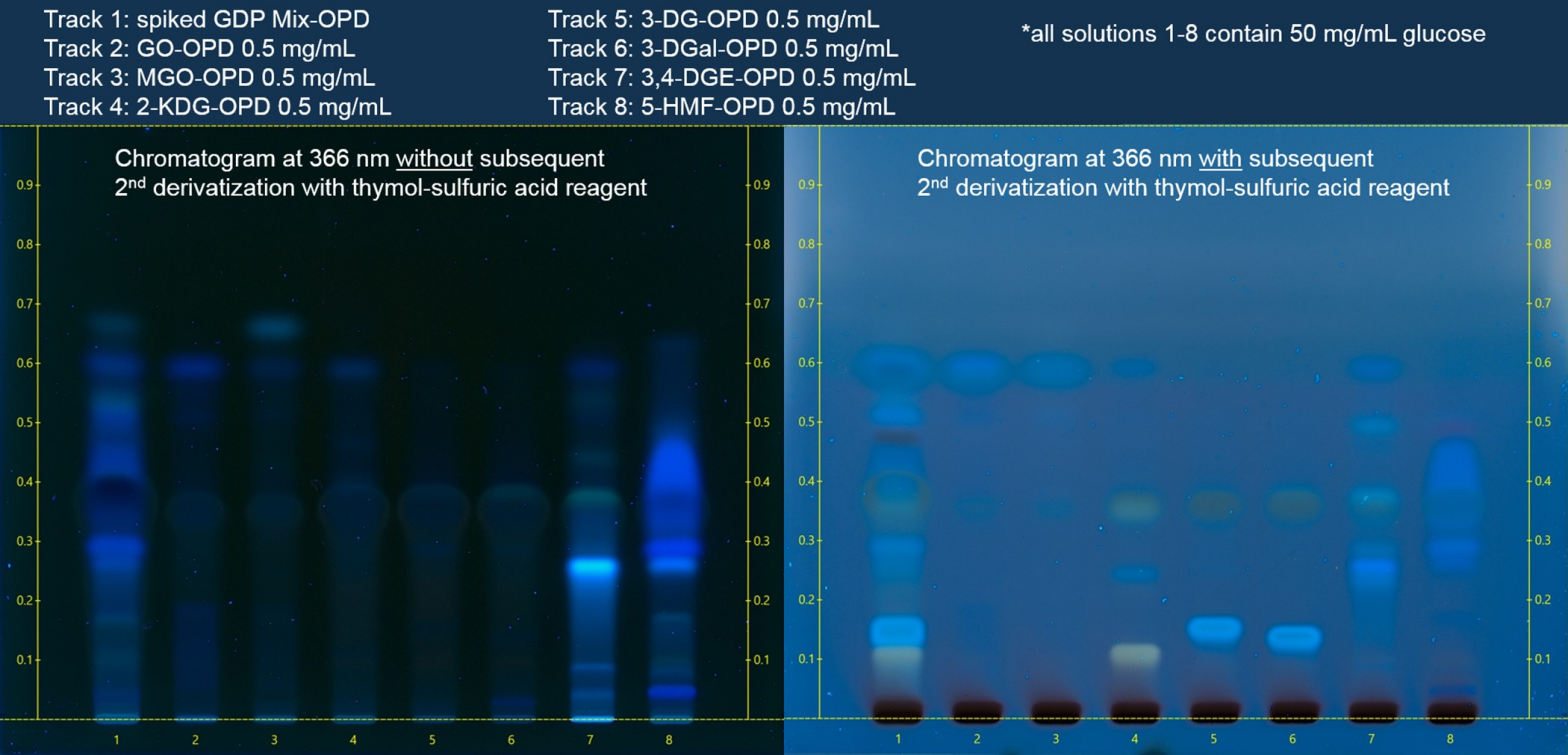
Chromatograms of the derivatized seven GDPs and a mixture (= spiked glucose solution) in the solvent 1,4-dioxane-toluene-glacial acetic acid (49:49:2, v/v/v). The chromatograms were shown at 366 nm without (2A) and with (2B) subsequent 2^nd^ derivatization with thymol-sulfuric acid reagent at concentration levels of 0.5 mg/mL.

The final eluent composition was 1,4-dioxane-toluene-glacial acetic acid (45:45:2, v/v/v). All derivatized analytes were used at concentrations of 0.5 mg/mL with volumes of 10 μL at an application length of 10.0 mm. The most suitable wavelength at which all spots of the derivatized GDPs could be identified, after they had been treated with thymol-sulfuric acid reagent, was 366 nm in fluorescence mode. Finally, glucose remained at the starting line, and background noise was low. The HPTLC plate was developed in a linear ascending manner (Fig 2B).

The resulting Rf values after developing the plate are shown in Table 2.

**Table 2:** Overview of the Rf values of the mixture 1,4-dioxane-toluene-glacial acetic acid (49:49:2, v/v/v) obtained after derivatization with thymol-sulfuric acid reagent.

Analyte	Rf value
2-KDG	0.102
3-DGal	0.124
3-DG	0.142
3,4-DGE	0.237
5-HMF	0.278
GO	0.571
MGO	0.571

These Rf values served as reference values for the entire method.

In Fig 2B it can be observed that the substances are optically well separated from each other. Glucose remains at the starting line and is colored black after spraying with thymol-sulfuric acid reagent. 2-KDG can be easily distinguished from 3-DG and 3-DGal by color. 5-HMF and 3,4-DGE (Rf 0.278 and 0.237) can be distinguished by color and by the height of their bands.

A limitation of the presented HPTLC method was, however, that both 3-DG/3-DGal (Rf = 0.124 and 0.142) and MGO/GO (Rf = 0.571) could only be detected in sum and it must be considered that cis/trans isomers exist for 3,4-DGE.

### 3.4 Advantage of colorimetric determination with thymol-sulfuric acid reagent for qualitative TLC analysis

According to the European Pharmacopoeia (Ph. Eur.) monograph for underivatized glucose [21], the derivatized GDPs were treated with 2 mL of thymol-sulfuric acid reagent as staining agent on a trial basis and were heated afterwards on a plate heater at 130°C for 10 minutes, as explained in section 2.2.3. The objective was to detect all substances in the UV range.

Table 3 gives an overview of the quality of visualization at the wavelengths 254 nm (absorption mode) and 366 nm (fluorescence mode), each with and without the 2^nd^ derivatization step with thymol-sulfuric acid reagent.

**Table 3:** Selection of the appropriate wavelength and derivatization to identify the GDPs.

1,4-dioxane-Toluene-Glacial acetic acid (49:49:2, v/v/v)	254	366	254	366
	- T/S	- T/S	+ T/S	+ T/S
MGO	++	+	+	++
GO	+	+	+	++
2-KDG	+	-	++	++
3-DG	+	-	++	++
3-DGal	+	-	++	++
3,4-DGE	+	++	0	+
Glucose	-	-	0	++
5-HMF	+	++	0	+

++ Very well visible

+ Clearly visible

0 Faintly visible

- Cannot be seen

-T/S Without 2^nd^ derivatization step with thymol-sulfuric acid reagent

+T/S With 2^nd^ derivatization step with thymol-sulfuric acid reagent

For comparison, Fig 2 shows chromatograms of the GDPs analyzed with 1,4-dioxane-toluene-glacial acetic acid (49:49:2, v/v/v) at 366 nm without (Fig 2A) and with (Fig 2B) subsequent thymol-sulfuric acid derivatization. The bands for 3,4-DGE and 5-HMF could unambiguously be determined without subsequent thymol-sulfuric acid derivatization. However, GO and MGO-OPD derivatives revealed only weak signals. Unfortunately, the derivatized products of 2-KDG, 3-DG, and 3-DGal could not be detected under the conditions used here.

To visualize the derivatized analytes GO, MGO, 2-KDG, 3-DG, and 3-DGal, the plate shown in Fig 2A was subsequently treated with thymol-sulfuric acid spray reagent and another photo was taken under illumination at 366 nm. Obviously, upon thymol-sulfuric-acid-treatment, all derivatized GDPs could be identified at 366 nm (Fig 2B). According to the Ph. Eur. monograph for glucose [21], spray derivatization served to visualize glucose, fructose, lactose, and sucrose. The so-called Seliwanoff reaction also worked under the condition that the GDPs had already been derivatized with OPD.

Fortunately, glucose remained at the starting line and did not show excessive tailing when it migrated.

### 3.5 Spot confirmation via LC-MS/MS

The objective of identifying the spots via LC-MS/MS was to evaluate the derivatization reactions of the seven GDPs and to clearly assign the spots. Preparation of the samples for identification by LC-MS/MS is described in section 2.2.3.

At least three bands were visible on each track of the HPTLC plate (Fig 2B). The black spot that remained at the starting line (Rf = 0) was identified as glucose. The spot at Rf = 0.350 corresponds to the derivatizing agent OPD. The most intensive spots, which indicated the presence of the reaction products, were analyzed by LC-MS/MS. Due to the formation of the novel quinoxaline chromophores in the resulting derivatization products, spots with fluorescing properties were expected at 366 nm in the UV scans.

A preliminary test showed that the derivatized GDPs were generally sensitive to the detection reagent thymol-sulfuric acid, but could no longer be evaluated by mass spectrometry. Therefore, the experiment was performed in duplicate. Only one plate was treated with thymol-sulfuric acid reagent to determine the migration distances of the derivatized GDPs on the plate. For the second HPTLC plate, this step was omitted and untreated HPTLC spots were scraped and further transferred for LC-MS/MS analysis.

The MS/MS spectra obtained for structure identification can be found in the (S8–S15 Figs). Deduced from the product ion spectra, respective MRM experiments were performed for qualitative analysis. The precalculated mass-to-charge ratios for all precursor and product ions under ESI conditions are listed S2 Table. Additionally, the most suitable ion transitions for the MRM experiment were also added for each analyte.

**Fig 3:**
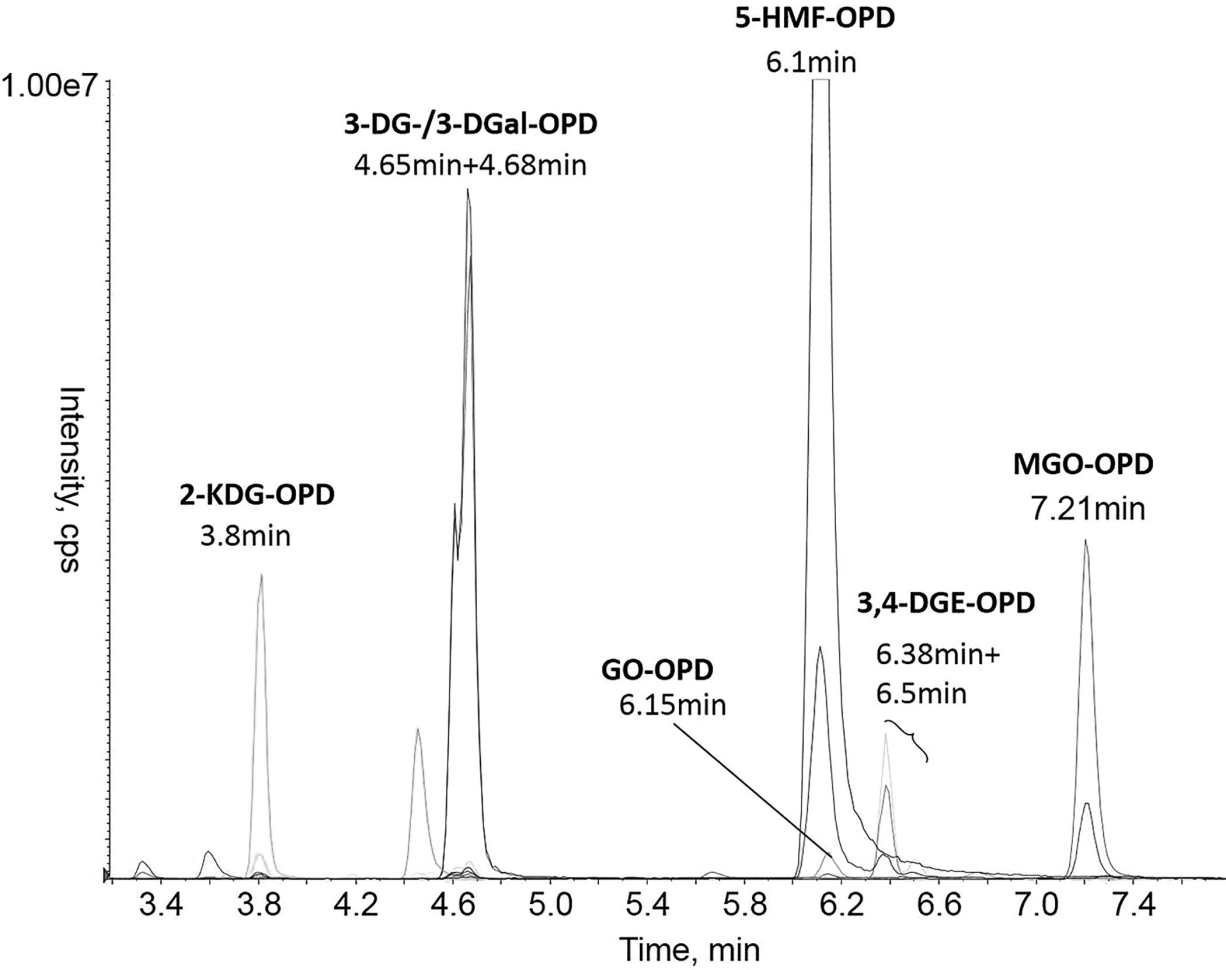
Extracted MRM chromatogram of the derivatized GDPs. The respective *m/z* ratios were MGO: *m/z* = 145.1/77, GO: *m/z* = 131.1/76.7, 3,4-DGE: *m/z* = 217.1/169.1, 3-DG/3-DGal: *m/z* = 235.1/199.1, 2-KDG: *m/z* = 251.1/173.2, and 5-HMF: *m/z* = 215.1/197.1.

All analytes could be separated by baseline separation via LC-MS/MS, except for the diastereomers 3-DG/3-DGal, which were evaluated here as total sum (Fig 3). All presumed spots of the analytes on the HPTLC plate coincided with the results of the LC-MS/MS analysis.

When comparing the HPTLC method with the LC-MS/MS method, both methods provide a good qualitative evaluation.

### 3.6 Purity of 3,4-DGE

The determination of purity via LC-MS/MS showed a clear signal for the derivatized 3,4-DGE. In addition, a few smaller signals indicated the presence of dimers formed during the derivatization process. However, this is negligible as, on the one hand, the signal strength was not significant and, on the other hand, it could be shown by the quantitative determination/validation that 3,4-DGE shows linear regression and thus that the content determinations are reproducible.

### 3.7 Stability of GDPs in the chosen eluent

The stability of the GDPs was tested both in the presence and in the absence of the added glucose, as well as in the aqueous and in the chosen eluent. Evaluation of the peak areas of solutions 1–4 showed that all analytes could be detected. Even after 75 minutes in the presence of the acidic solvent, no further signals were detected by LC-MS/MS. This indicates that the derivatized GDPs did not degrade further to any significant degree here and that the derivatized GDPs are stable in the acidic solvent. The signal strength of solutions 3 and 4 was even slightly higher after exposure to the solvent. This can be explained by the fact that more GDPs were protonated in the acidic solvent and were thus also detected in higher quantities by mass spectrometry. However, this effect can be neglected here, too, as the signal strength was not significantly higher and it could be shown by quantitative determination/validation that the derivatized GDPs show linear or polynomial regression and thus that the content determinations are reproducible. Therefore, either a significantly higher acid strength of the solvent or a higher acid concentration is required to degrade the derivatized GDPs.

### 3.8 Quantitative analysis, method validation, and analysis of a finished medicinal product

**Fig 4:**
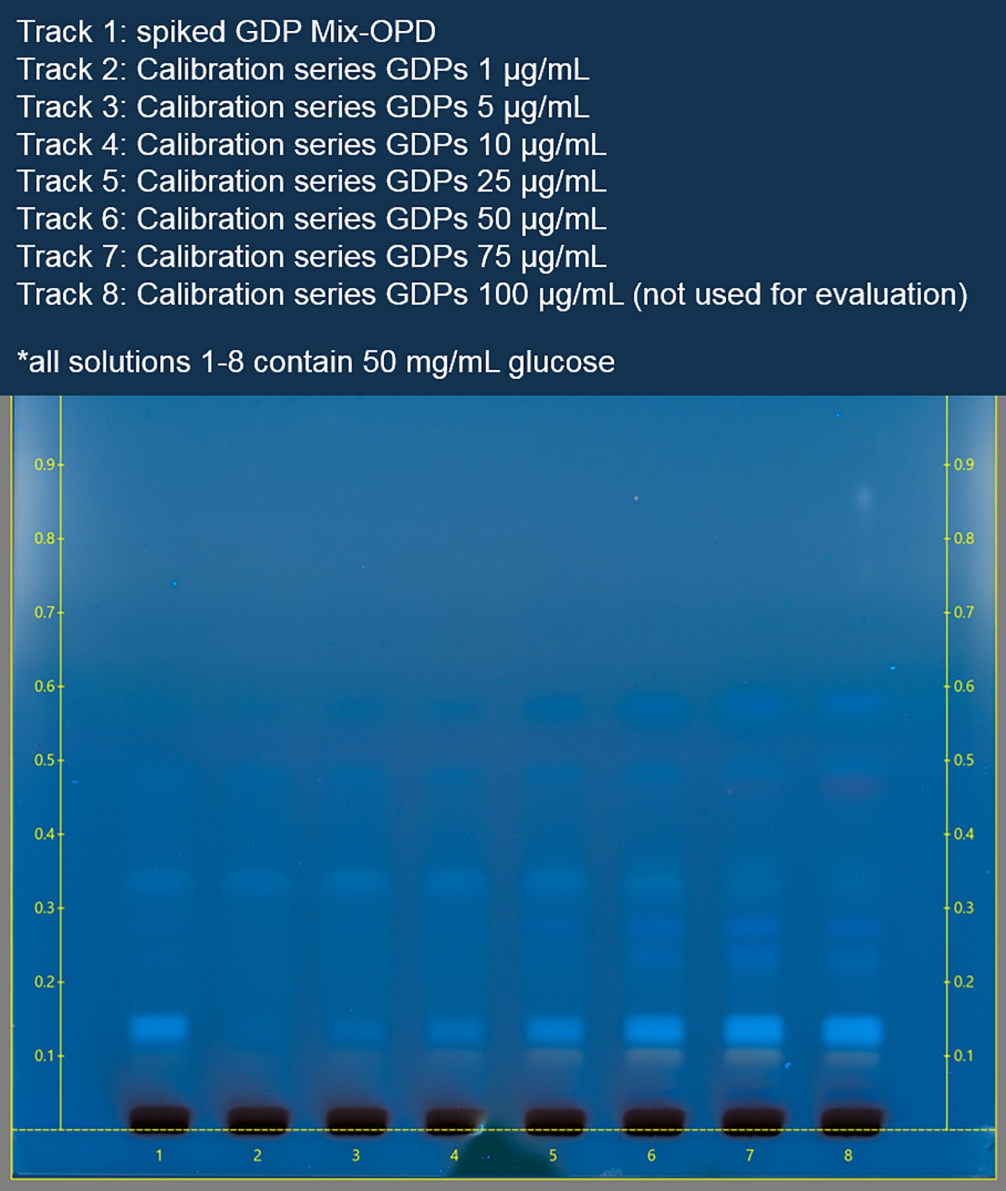
Developed, sprayed, and heated plate at 366nm. Track 1 shows the spiked 5% glucose solution; tracks 2–7 contain the calibration solutions in ascending concentrations (1 μg/mL—75 μg/mL).

For quantitative analysis of the GDPs, an HPTLC analysis was carried out as described in section 2.2.3. All six calibration solutions, each consisting of 7 GDPs, 50 mg/mL glucose, and 0.75 mg/mL OPD with concentration levels between 1 and 75 μg/mL were analyzed. Additionally, a mix with all expected GDPs in a 5% glucose solution was prepared that contained 50 mg/mL glucose and 0.75 mg/mL OPD and was also analyzed. The plates were developed as described in section 2.2.3 in the final eluent composition, then treated with the spray reagent and subsequently heated for 10 min at 130°C (Fig 4).

Chromatograms were recorded from the six calibration solutions of the OPD-GDPs at the specific wavelengths previously ascertained and calibration curves were calculated by means of the respective absorption rates. The final wavelength for quantification and the Rf value for each analyte are shown in Table 4. 3,4-DGE was evaluated via Visualizer 2, the remaining analytes via CAMAG TLC-SCANNER 3. HPTLC chromatograms and regression calculations can be found in the (S22–S31 Figs).

**Table 4:** Rf values and measurement settings of the GDPs.

GDP	Rf	Wavelength	Absorption/ Fluorescence
2-KDG	0.102	420	Absorption
3-DG/3-DGal	0.134	370	Absorption
3,4-DGE	0.237	366	Fluorescence
5-HMF	0.278	330	Absorption
GO/MGO	0.571	330	Absorption

These wavelengths and modes were used for the entire method development process.

#### 3.8.1 Selectivity

The HPTLC method developed is selective for the GDPs investigated in this study, as confirmed by the observation that the glucose matrix did not affect the Rf values of the derivatized GDPs. Glucose was not derivatized at all, remained at the starting position of the HPTLC plate, and did not interfere with the quantitative analysis of the GDPs. One limitation with regard to selectivity, however, is that the method does not sufficiently separate between 3-DG/DGal and GO/MGO.

#### 3.8.2 Linearity

All analytes except 3,4-DGE were evaluated via TLC Scanner. Due to the fact that the signal of 3,4-DGE could not be identified as a peak in the scan of the plate, but rather as a peak in the scan of the image, 3,4-DGE was evaluated in fluorescence mode from an image at 366 nm via Visualizer 2. Depending on the quality of the fit, either linear or nonlinear regression was selected. The derivatized 3,4-DGE followed linear regression; the derivatized analytes 5-HMF, GO/MGO, 2-KDG, and 3-DG/3-DGal followed polynomial regression. Derivatized 3,4-DGE correlated well at R^2^ = 0.982, and 5-HMF very well at R^2^ = 0.997. All other analytes had excellent correlation coefficients (R^2^ = 0.999). Regression parameters were computed using Excel (Microsoft Office Professional Plus 2016).

#### 3.8.3 Range

The range of GDP derivatives was 1–75 μg/mL for 3,4-DGE and 5-HMF, 1–50 μg/mL for 2-KDG, 2–150 μg/mL for GO/MGO, and 10–150 μg/mL for 3-DG/3-DGal. For reasons of good correlation, the calibration solution with a concentration of 75 μg/mL for 2-KDG was not considered for the regression. Due to the sum evaluation of GO/MGO and 3-DG/3-DGal, the range doubled here compared to the range of the single GDPs.

The range was calculated as a compromise including all expected GDP concentrations. The lower limit of the range was set by the LOQ.

#### 3.8.4 LOD

To determine the LOD, signal-to-noise ratios of 3:1 were obtained from the chromatograms. All derivatized GDPs had a LOD of 1 μg/mL, except for GO/MGO and 3-DG/3-DGal. These were at higher detection limits of < 2 μg/mL (GO/MGO) or 2 μg/mL (3-DG/3-DGal).

#### 3.8.5 LOQ

To determine the LOQ, signal-to-noise ratios of 10:1 were taken from the chromatograms. Here, 3-DG/3-DGal showed a LOQ of 10 μg/mL, 2-KDG, 3,4-DGE, and 5-HMF a LOQ of 5 μg/mL. GO/MGO showed a LOQ of 2 μg/mL.

#### 3.8.6 Accuracy

Accuracy was reported as % recovery and tested in order to exclude possible systematic errors. The mean recovery (in %) was performed at 3 concentrations with 3 replicates each for the concentration levels 5, 25, and 50 μg/mL after a 16-h derivatization period with 0.75 mg/mL OPD. Accuracy ranged from 86.4 to 112.7%. It can be seen that the accuracy deviates most clearly at the higher concentration of 50 μg/mL with ± 13.6%, while at the lower concentration of 5 μg/mL only a deviation of ± 8.9% can be observed. This may be due to the reaction rate of the GDPs with the derivatization reagent.

#### 3.8.7 Precision

The intraday precision was evaluated by analyzing three different concentrations with three replicates of each concentration. Intraday precision was calculated as RSD % for peak height or width, respectively. It ranged from 0.4–0.9% for GO/MGO, 2.5–3.0% for 5-HMF, 2.0–5.2% for 3,4-DGE, 0.7–5.5% for 3-DG/3-DGal, and 5.8–14.2% for 2-KDG in spiked 5% glucose solutions. Only 2-KDG shows higher RSD % values than the other GDPs.

The results verify that the procedure described here provides a reliable and precise method for quantifying GDPs in glucose solutions in the specified range. All parameters of the method validation are presented in Tables 5–7.

**Table 5:** Method validation parameters.

Analyte	Regression	R^2^	Evaluated by peak height/ peak area	Range [μg/mL]	LOD [μg/mL]	LOQ [μg/mL]	Expected level [μg/mL]
2-KDG	y = -1E-05x2 + 0,0025x + 0,0018	0.999	Height	1–50	1	5	7
3-DG/3-DGal	y = -1E-07x2 + 7E-05x - 1E-04	0.999	Area	10–150	2	10	45/25
3,4-DGE	y = 0.0039x - 0.0149	0.982	Height	1–75	1	5	5
5-HMF	y = -3E-07x2 + 9E-05x + 0,0005	0.997	Area	1–75	1	5	5
GO/MGO	y = -1E-07x2 + 1E-04x + 0,0036	0.999	Area	2–150	< 2	2	1/1

**Table 6:** Precision in terms of % relative standard deviation (RSD) for replicate measurements (n = 3) at three different concentrations.

GDP	GDP conc. [μg/mL]	Mean [μg/mL] ± SD	RSD %
GO/MGO	5/5 (interpreted as 10 μg/mL)	11.1 ± 0.1	0.9
	25/25 (interpreted as 50 μg/mL)	47.8 ± 0.4	0.8
	50/50 (interpreted as 100 μg/mL)	106.3 ± 0.5	0.4
5-HMF	5	5.2 ± 0.1	2.6
	25	24.0 ± 0.6	2.5
	50	49.9 ± 1.5	3.0
3,4-DGE	5	4.1 ± 0.2	5.2
	25	25.5 ± 0.5	2.0
	50	50.3 ± 2.1	4.3
3-DG/3-DGal	5/5 (interpreted as 10 μg/mL)	10.7 ± 0.6	5.5
	25/25 (interpreted as 50 μg/mL)	52.6 ± 0.4	0.7
	50/50 (interpreted as 100 μg/mL)	109.1 ± 2.0	1.8
2-KDG	5	4.2 ± 0.6	14.2
	25	24.2 ± 1.4	5.8
	50	59.7 ± 4.2	7.0

**Table 7:** Accuracy reported as percent recovery for 3 concentrations/3 replicates each of the total analytical procedure, (n = 9 determinations).

GDP	GDP conc. [μg/mL]	Mean [μg/mL] ± SD	% Recovery
GO/MGO	5/5 (interpreted as 10 μg/mL)	10.9 ± 0.2	108.9
	25/25 (interpreted as 50 μg/mL)	43.8 ± 5.9	87.6
	50/50 (interpreted as 100 μg/mL)	104.3 ± 3.6	104.3
5-HMF	5	4.9 ± 0.5	97.8
	25	24.2 ± 0.5	96.8
	50	49.5 ± 2.5	99.0
3,4-DGE	5	5.3 ± 0.4	106.7
	25	25.7 ± 0.3	102.8
	50	56.3 ± 2.6	112.7
3-DG/3-DGal	5/5 (interpreted as 10 μg/mL)	10.7 ± 0.8	91.2
	25/25 (interpreted as 50 μg/mL)	51.4 ± 0.6	98.7
	50/50 (interpreted as 100 μg/mL)	112.7 ± 5.2	92.4
2-KDG	5	5.4 ± 0.2	107.3
	25	25.2 ± 2.5	100.8
	50	43.2 ± 1.6	86.4

Table 8 shows the GDP concentrations determined from a 5% glucose solution of an authorized medicinal product. The medicinal product did not contain further additives. It was a 5% glucose solution used as a carrier solution for compatible electrolyte concentrates and drugs. The GDP content was determined in 3 replicate measurements. The corresponding HPTLC plates can be found in the (S32 and S33 Figs).

**Table 8:** A 5% glucose solution of an authorized medicinal product from a 250-mL polypropylene bottle was analyzed.

GDP	Mean [μg/mL] ± SD
GO/MGO	n.a.
2-KDG	n.a.
3-DG/3-DGal	148.4 ± 1.8
3,4-DGE	9.3 ± 0.2
5-HMF	19.1 ± 3.2

Compared to the expected levels of GDPs described in the literature (Table 1), neither GO/MGO nor 2-KDG was found in the authorized 5% glucose solution. However, a value almost twice as high as expected was identified for 3-DG/3-DGal (expected value in sum: 70 μg/mL, detected value: 148.4 μg/mL). In addition, for 5-HMF instead of the expected 5 μg/mL, we found 19.1 μg/mL, and for 3,4-DGE instead of the expected 5 μg/mL, we found 9.3 μg/mL here. The observed deviations in GDPs as compared to our level of expectation may be due to different autoclaving procedures of the manufacturer or to different packaging material (polypropylene bottles, duran glass bottles etc.). More examples of marketed products will be analysed in a follow-up study.

Furthermore, the concentration levels of derivatized GDPs (Table 1) may serve as reference values for future studies in which heat-sterilized glucose solutions are tested. We have shown that this method is sufficiently selective and sensitive to qualitatively and quantitatively determine the expected content of GDPs in glucose solutions for parenteral use (Tables 5–8). The LC-MS/MS method was used solely for purposes of qualitative analysis. By repeating the HPTLC method several times, we found that the method is not sensitive to temperature and light.

It must be mentioned that for the pairs GO-OPD/MGO-OPD and 3-DG-OPD/ 3-DGal-OPD only the total content could be determined. The resolution of the HPTLC method is poorer than the previously reported (U)HPLC-UV /DAD /MS/MS methods. On the other hand, such a pair-wise quantification may very well suit the needs of the routine user of such analytical approaches and may fulfill regulatory requirements, presuming that the toxic potential of the underlying isomers is comparable [31–34].

Qualitative evaluation by LC-MS/MS confirmed the suspected associated spots of 3,4-DGE-OPD and 5-HMF-OPD. For cis/trans assignments of the bands observed by HPTLC, however, it is necessary to elucidate the structures by LS-MS/MS.

No HPTLC method has yet been described for determining GDPs in glucose solutions. Therefore, the HPTLC method presented here constitutes an alternative approach to rapidly determine GDPs in glucose solutions for parenteral use.

## 4. Conclusion

A qualitative and quantitative HPTLC-based analysis of GDPs, as they are typically found in heat-sterilized glucose solutions for parenteral administration, is described in this study.

A mobile phase was developed by which all GDPs could be separated, except for GO/MGO and the isomer pair 3-DG/3-DGal, where glucose does not disturb the separation of GDPs. The HPTLC method provides a quick means of specifying all degradation products in the solution without too much interference from the large excess of glucose as the glucose remains at the starting line. This might represent an advantage over alternative LC-MS/MS approaches under reversed-phase conditions, as described in the literature, where high amounts of glucose or OPD could possibly affect the separation and contaminate such a sophisticated setting.

The identity of GDPs separated by the HPTLC method developed in this work was verified by LC-MS/MS. This method might also represent a beneficial addition to the pharmacopeial test on impurity. For quantitative or limit analysis of GDPs by TLC, commercially available reference solutions at specific concentrations could be prepared, applied to the TLC plate, and compared with the glucose solution. By comparing the GDP spots on the HPTLC plate with the spots of the standards in terms of size and color intensity, a (semi-)quantitative analysis could be performed. For routine analysis, the LC-MS/MS verification used in the method development here would therefore no longer be necessary.

The method presented in our study was successfully validated using the ICH Q2(R1) guideline. It is easy to use, fast, and sensitive and can be applied to screen and measure the content of GDPs in heat-sterilized glucose solutions.

## Supporting information

S1 Table Overview of tested mobile phase compositions. (DOCX)

S2 Table LC-MS/MS parameters of all investigated GDPs derivatized with OPD. (DOCX)

S1 Fig TLC-plate with GDP-OPDs upon elution in methanol-ethyl acetate (30:70, v/v) and staining with thymol-sulfuric acid; image taken at the wavelength of 366 nm. (TIFF)

S2 Fig TLC-plate with GDP-OPDs upon elution in methanol-dichloromethane (30:70, v/v) and staining with thymol-sulfuric acid; image taken at the wavelength of 366 nm. (TIFF)

S3 Fig TLC-plate with GDP-OPDs upon elution in methanol-toluene (50:50, v/v) and staining with thymol-sulfuric acid; image taken at the wavelength of 366 nm. (TIFF)

S4 Fig TLC-plate with GDP-OPDs upon elution in 1,4 dioxane-toluene (95:5, v/v) and staining with thymol-sulfuric acid; image taken at the wavelength of 366 nm. (TIFF)

S5 Fig TLC-plate with GDP-OPDs upon elution in 1,4 dioxane-toluene-glacial acetic acid (45:45:10, v/v/v) and staining with thymol-sulfuric acid; image taken at the wavelength of 366 nm. (TIFF)

S6 Fig Derivatized GDP solutions with and without added glucose in order to test the influence of glucose on the consumption of the derivatization reagent OPD. (TIFF)

S7 Fig Derivatized GDP solutions and mix with added glucose in order to test the influence of the smaller migration distance (7cm). (TIFF)

S8 Fig Product ion mass spectrum of 2-KDG-OPD with hypothetical structure elucidation, m/z = 251.1, CE = 20 eV by LC-MS/MS. (TIFF)

S9 Fig Product ion mass spectrum of 3-DG-OPD with hypothetical structure elucidation, m/z = 235.1, CE = 20 eV by LC-MS/MS. (TIFF)

S10 Fig Product ion mass spectrum of 3-DGal-OPD with hypothetical structure elucidation, m/z = 235.1, CE = 20 eV by LC-MS/MS. (TIFF)

S11 Fig Product ion mass spectrum of 5-HMF-OPD with hypothetical structure elucidation, m/z = 215.1, CE = 25 eV by LC-MS/MS. (TIFF)

S12 Fig Product ion mass spectrum of GO-OPD with hypothetical structure elucidation, m/z = 131.1, CE = 40 eV by LC-MS/MS. (TIFF)

S13 Fig Product ion mass spectrum of 3,4-DGE-OPD 1^st^ isomer with hypothetical structure elucidation, m/z = 217.1, CE = 20 eV by LC-MS/MS. (TIFF)

S14 Fig Product ion mass spectrum of 3,4-DGE-OPD 2^nd^ isomer with hypothetical structure elucidation, m/z = 217.1, CE = 20 eV by LC-MS/MS. (TIFF)

S15 Fig Product ion mass spectrum of MGO-OPD with hypothetical structure elucidation, m/z = 145.1, CE = 40 eV by LC-MS/MS. (TIFF)

S16 Fig Influence of the derivatization time and the concentration of the derivatization reagent for GO at a concentration level of 10 μg/mL. (TIFF)

S17 Fig Influence of the derivatization time and the concentration of the derivatization reagent for MGO at a concentration level of 10 μg/mL. (TIFF)

S18 Fig Influence of the derivatization time and the concentration of the derivatization reagent for 2-KDG at a concentration level of 10 μg/mL. (TIFF)

S19 Fig Influence of the derivatization time and the concentration of the derivatization reagent for 3-DG/3-DGal at a concentration level of 10 μg/mL. (TIFF)

S20 Fig Influence of the derivatization time and the concentration of the derivatization reagent for 3,4-DGE at a concentration level of 10 μg/mL. (TIFF)

S21 Fig Influence of the derivatization time and the concentration of the derivatization reagent for 5-HMF at a concentration level of 10 μg/mL. (TIFF)

S22 Fig Chromatogram of 2-KDG in the concentration range of 1–50 μg/mL at 420 nm recorded in absorption mode at Rf = 0.102. (TIFF)

S23 Fig Chromatogram of 3-DG/3-DGal in the concentration range of 10–150 μg/mL at 370 nm recorded in absorption mode at Rf = 0.134. (TIFF)

S24 Fig Chromatogram of 3,4-DGE in the concentration range of 1–75 μg/mL at 366 nm recorded in fluorescence mode at Rf = 0.237. (TIFF)

S25 Fig Chromatogram of 5-HMF in the concentration range of 1–75 μg/mL at 330 nm recorded nm in absorption mode at Rf = 0.278. (TIFF)

S26 Fig Chromatogram GO/MGO in the concentration range of 2–150 μg/mL at 330 nm recorded in absorption mode at Rf = 0.571. (TIFF)

S27 Fig Polynomial regression of 2-KDG at a concentration level of 1–50 μg/mL at 420 nm in absorption mode at Rf = 0.102. (TIFF)

S28 Fig Polynomial regression of 3-DG/3-DGal at a concentration level of 10–150 μg/mL at 370 nm in absorption mode at Rf = 0.134. (TIFF)

S29 Fig Linear regression of 3,4-DGE at a concentration level of 1–75 μg/mL at 366 nm in fluorescence mode at Rf = 0.237. (TIFF)

S30 Fig Polynomial regression of 5-HMF at a concentration level of 1–75 μg/mL at 330 nm in absorption mode at Rf = 0.278. (TIFF)

S31 Fig Polynomial regression of GO/MGO at a concentration level of 2–150 μg/mL at 330 nm in absorption mode at Rf = 0.571. (TIFF)

S32 Fig Analysis of a 5% finished drug solution at 366 nm without subsequent 2^nd^ derivatization with thymol-sulfuric acid reagent. (TIFF)

S33 Fig Analysis of a 5% finished drug solution at 366 nm with subsequent 2^nd^ derivatization with thymol-sulfuric acid reagent. (TIFF)

S1 Raw images (PDF)

S1 Graphical abstract (TIF)
