# Correction: Identification and quantification of glucose degradation products in heat-sterilized glucose solutions for parenteral use by thin-layer chromatography

**DOI:** 10.1371/journal.pone.0322667

**Published:** 2025-04-10

**Authors:** The PLOS One Staff

There is an issue with the Supporting Information files. The originally submitted supplementary files, which were reviewed alongside the manuscript, contained both text and figures. Unfortunately, only the tables and figures were included on the published article, while the accompanying text was omitted. The publisher apologises for this error. Please disregard the originally published supplementary files and find this correct and complete supplementary file here, which includes essential details such as the description of eluent development (necessary for interpreting HPTLC plates from S1 Fig to S5 Fig in S1 File), the Statistical Analysis section, and the reference list. This supplementary file has also been updated with revised numbering to align with the original article.

Please view the correct S1 File in S1 File below.

## Supporting information

S1 File Supplementary data file. This dataset provides an overview of empirically tested eluent compositions for the qualitative separation of derivatized GDPs (GO, MGO, 2-KDG, 3-DG, 3-DGal, 3,4-DGE, and 5-HMF) using OPD. Various solvent systems were explored to optimize chromatographic resolution, with results documented in TLC images (Figs S1-S7) and LC-MS/MS spectra (Figs S8-S15). The dataset also evaluates derivatization efficiency (Figs S16-S21) and HPTLC quantification (Figs S22-S31), detailing calibration and regression analysis.
(DOCX)
