# Supplementary material for: Identification and quantification of glucose degradation products in heat-sterilized glucose solutions for parenteral use by thin-layer chromatography
Source: PLoS One. 2021 Jul 2;16(7):e0253811. doi: 10.1371/journal.pone.0253811 (PMC8253424; doi:10.1371/journal.pone.0253811)
Supplement: S1 Table — (DOCX) [file pone.0253811.s001.docx]

**S1 Table. Overview of tested mobile phase compositions.**

| **Eluent ( v/v or v/v/v)** | **Figure** |
| --- | --- |
| Water-methanol-glacial acetic acid-dichloroethane (10:15:25:50, v/v/v/v) | Data not shown |
| Methanol-ethyl acetate (30:70, v/v) | S1 Fig |
| Methanol-dichloromethane (30:70, v/v) | S2 Fig |
| Methanol-toluene (50:50, v/v) | S3 Fig |
| 1,4-Dioxane-toluene (95:5, v/v) | S4 Fig |
| 1,4-Dioxane-toluene-glacial acetic acid (45:45:10, v/v/v) | S5 Fig |

**All chromatograms were evaluated at 366 nm after being stained with thymol-sulfuric acid.**
