# Supplementary material for: Identification and quantification of glucose degradation products in heat-sterilized glucose solutions for parenteral use by thin-layer chromatography
Source: PLoS One. 2021 Jul 2;16(7):e0253811. doi: 10.1371/journal.pone.0253811 (PMC8253424; doi:10.1371/journal.pone.0253811)
Supplement: S2 Table — (DOCX) [file pone.0253811.s002.docx]

**S2 Table. LC-MS/MS parameters of all investigated GDPs derivatized with OPD.**

| **Analyte** | **Q1**  **[m/z]** | **Q3**  **[m/z]** | **Dwell time**  **[msec]** | **CE**  **[eV]** | **DP**  **[eV]** |
| --- | --- | --- | --- | --- | --- |
| 2-KDG-OPD | 251.1 | 173.2 | 50 | 20 | 100 |
|  | 251.1 | 145.1 | 50 | 20 | 100 |
| 3-DG-OPD/  3-DGal-OPD | 235.1 | 199.1 | 50 | 20 | 100 |
|  | 235.1 | 217.1 | 50 | 20 | 100 |
| 5-HMF-OPD | 215.1 | 197.1 | 50 | 25 | 100 |
|  | 215.1 | 169.1 | 50 | 25 | 100 |
| GO-OPD | 131.1 | 104 | 50 | 40 | 100 |
|  | 131.1 | 76.7 | 50 | 40 | 100 |
| 3,4-DGE-OPD | 217.1 | 169.1 | 50 | 20 | 100 |
|  | 217.1 | 181.1 | 50 | 20 | 100 |
| MGO-OPD | 145.1 | 118.1 | 50 | 40 | 100 |
|  | 145.1 | 77 | 50 | 40 | 100 |
