# Supplementary figures and images for: Identification and quantification of glucose degradation products in heat-sterilized glucose solutions for parenteral use by thin-layer chromatography
Source: PLoS One. 2021 Jul 2;16(7):e0253811. doi: 10.1371/journal.pone.0253811 (PMC8253424; doi:10.1371/journal.pone.0253811)

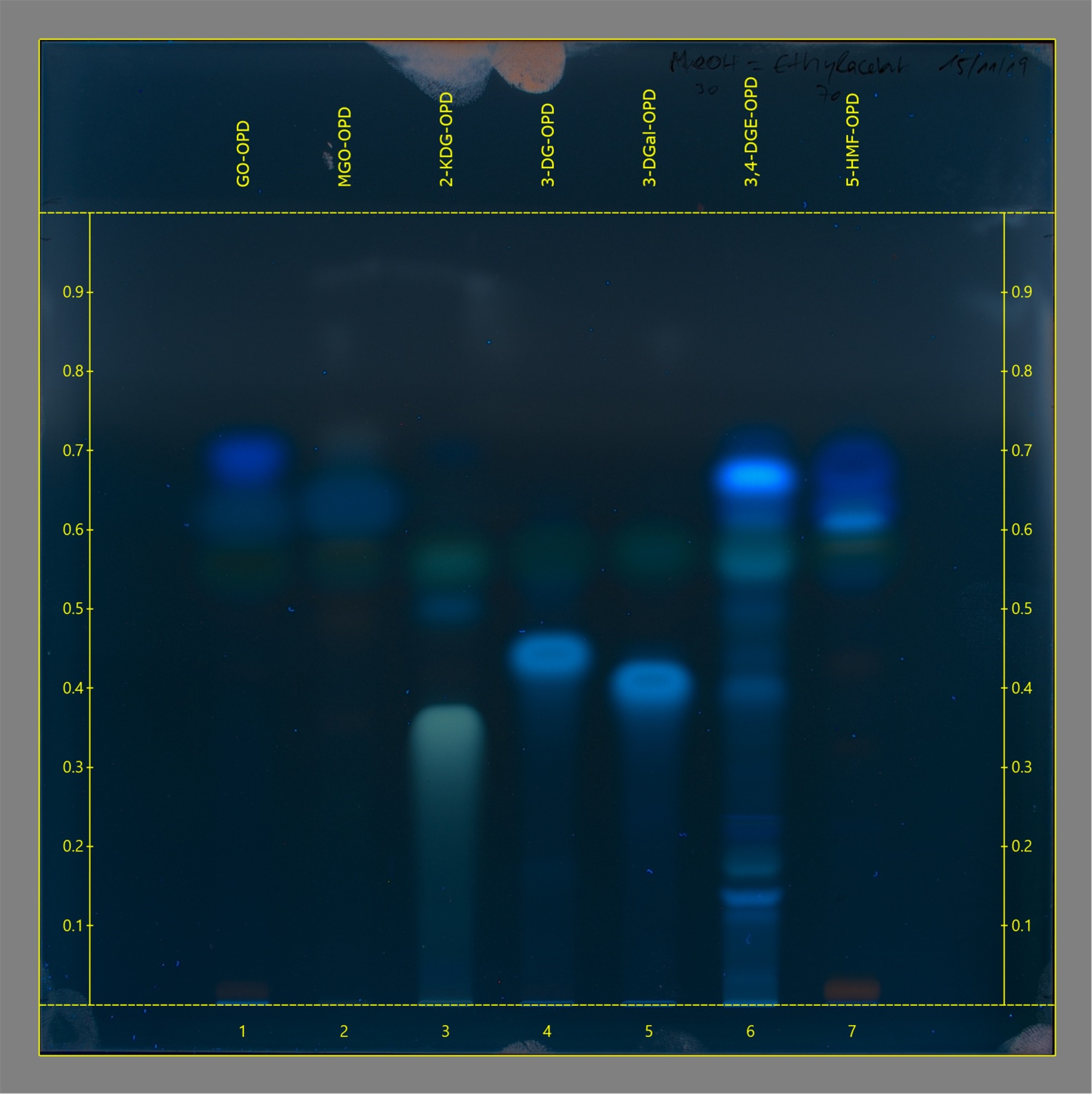

Supplement: S1 Fig — (TIFF) [file pone.0253811.s003.tiff]

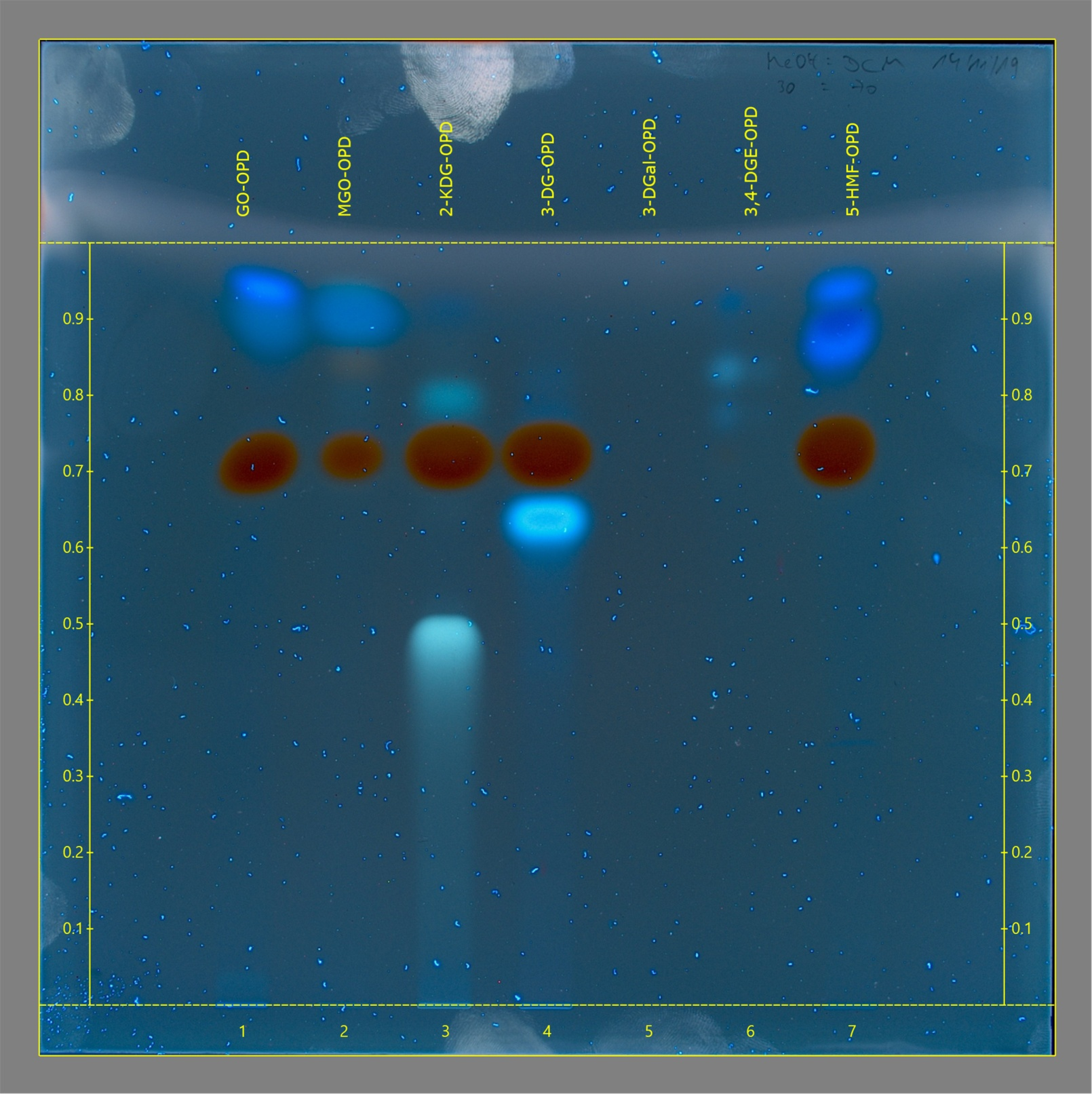

Supplement: S2 Fig — (TIFF) [file pone.0253811.s004.tiff]

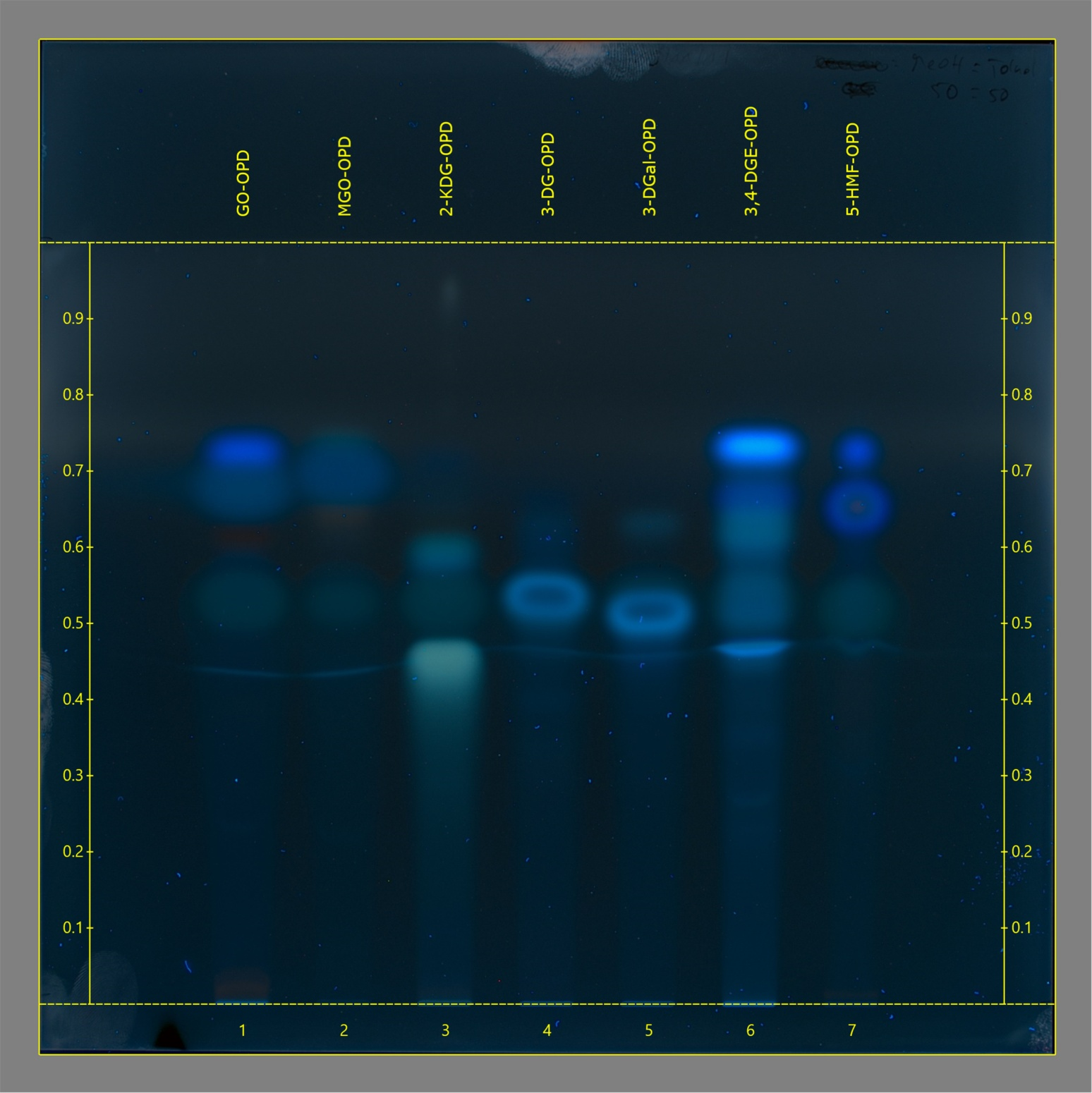

Supplement: S3 Fig — (TIFF) [file pone.0253811.s005.tiff]

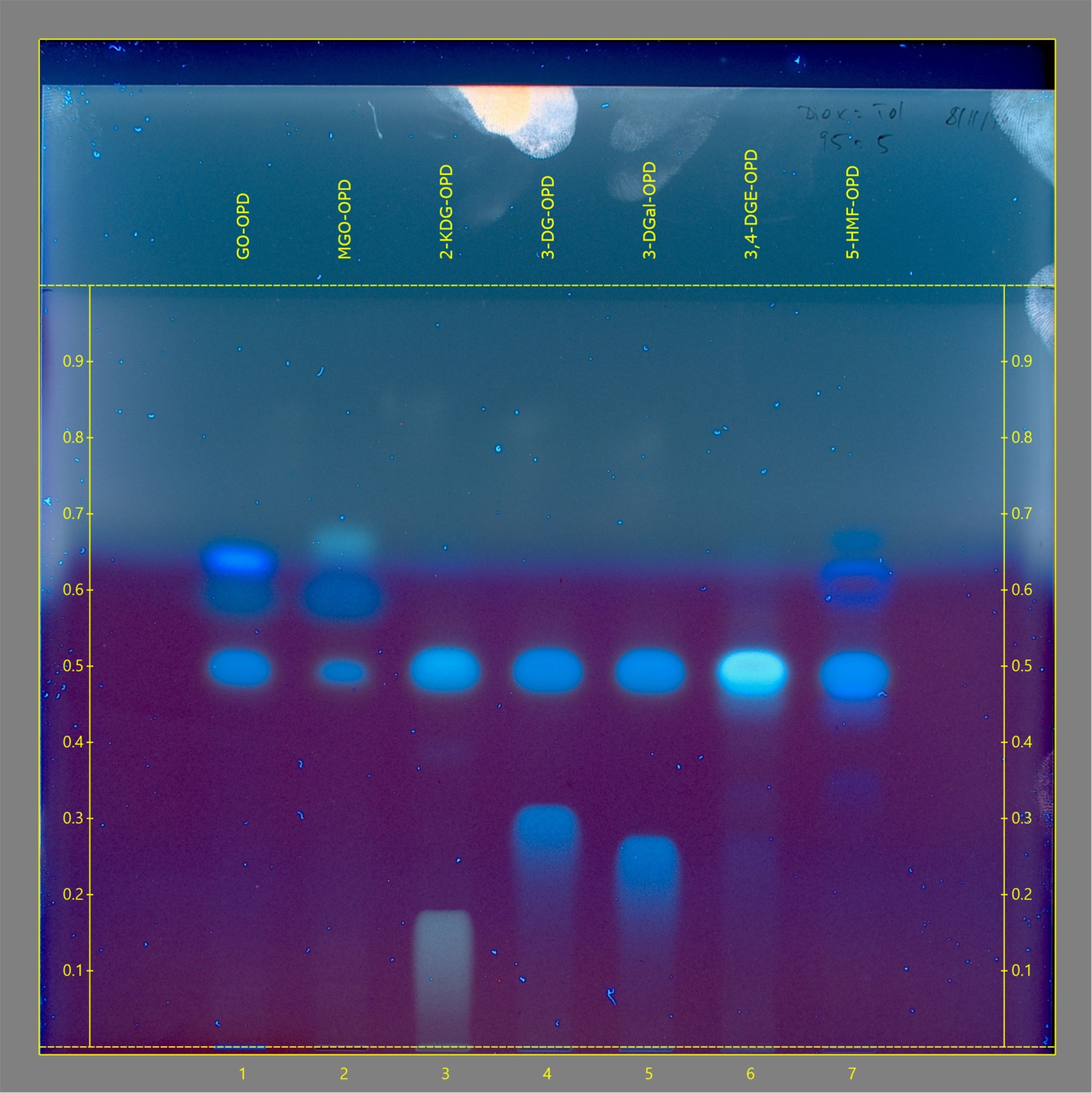

Supplement: S4 Fig — (TIFF) [file pone.0253811.s006.tiff]

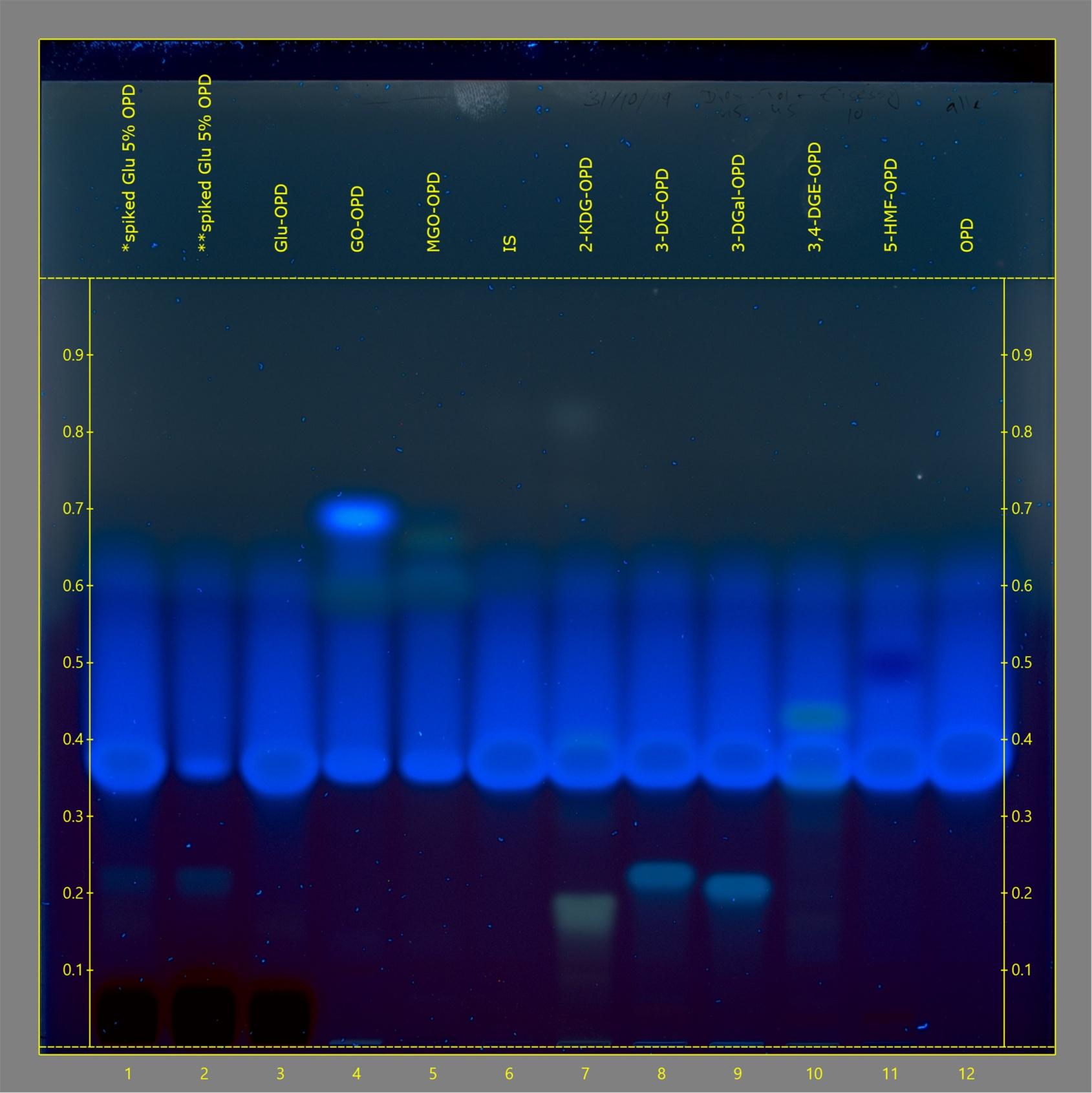

Supplement: S5 Fig — (TIFF) [file pone.0253811.s007.tiff]

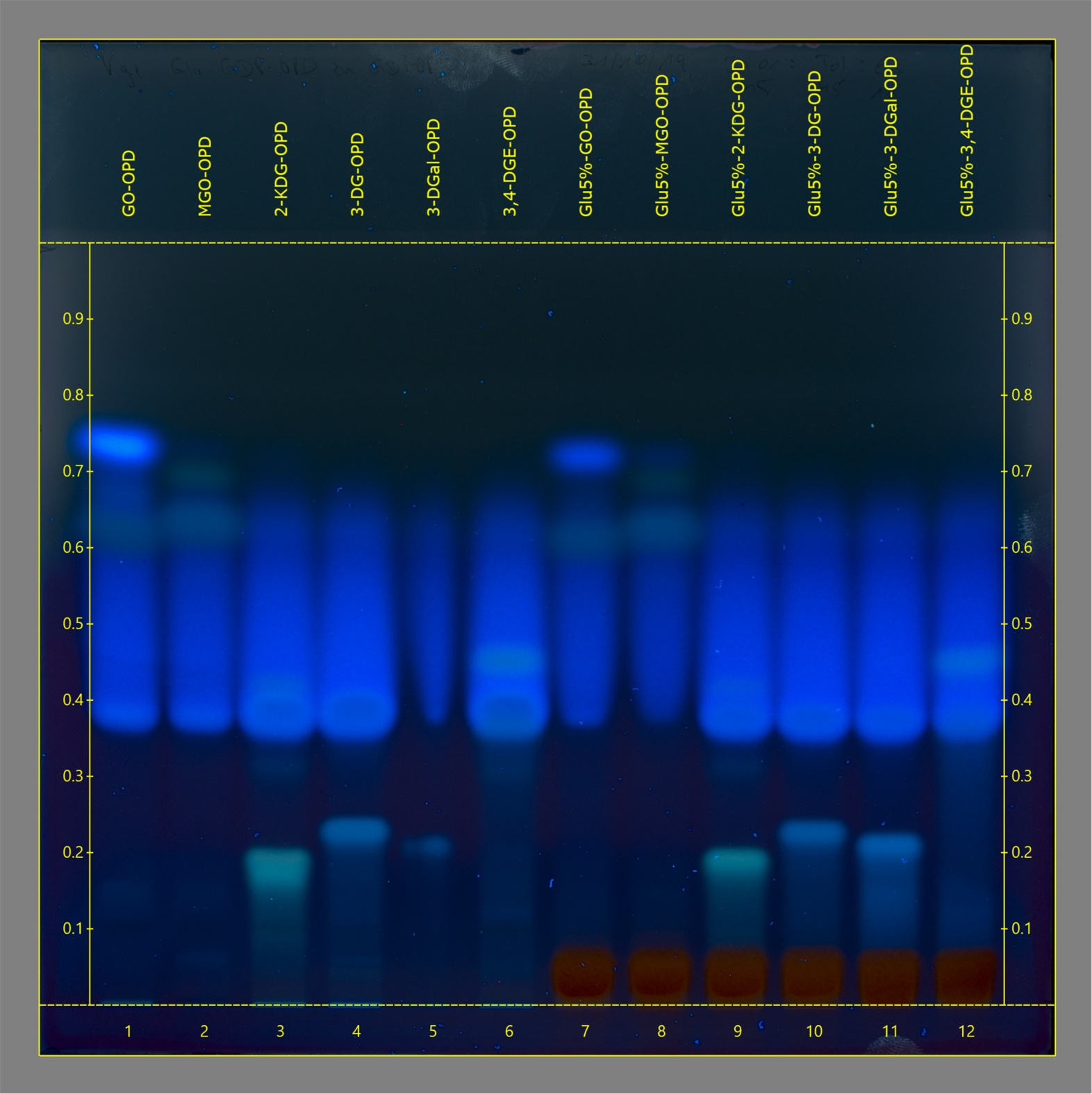

Supplement: S6 Fig — (TIFF) [file pone.0253811.s008.tiff]

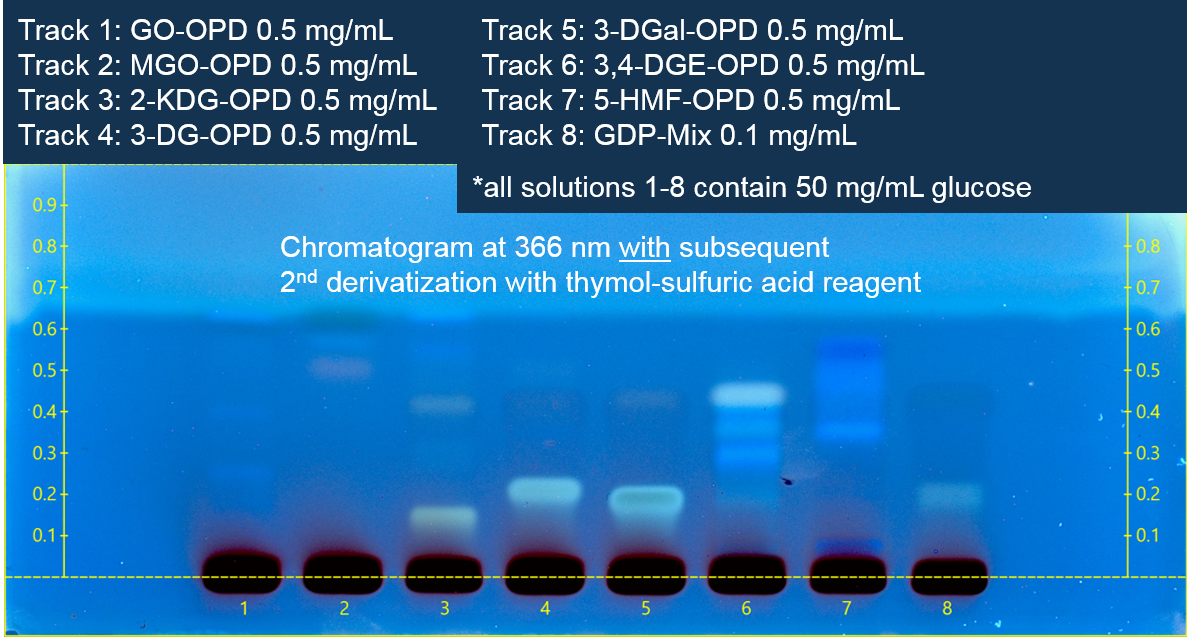

Supplement: S7 Fig — (TIFF) [file pone.0253811.s009.tiff]

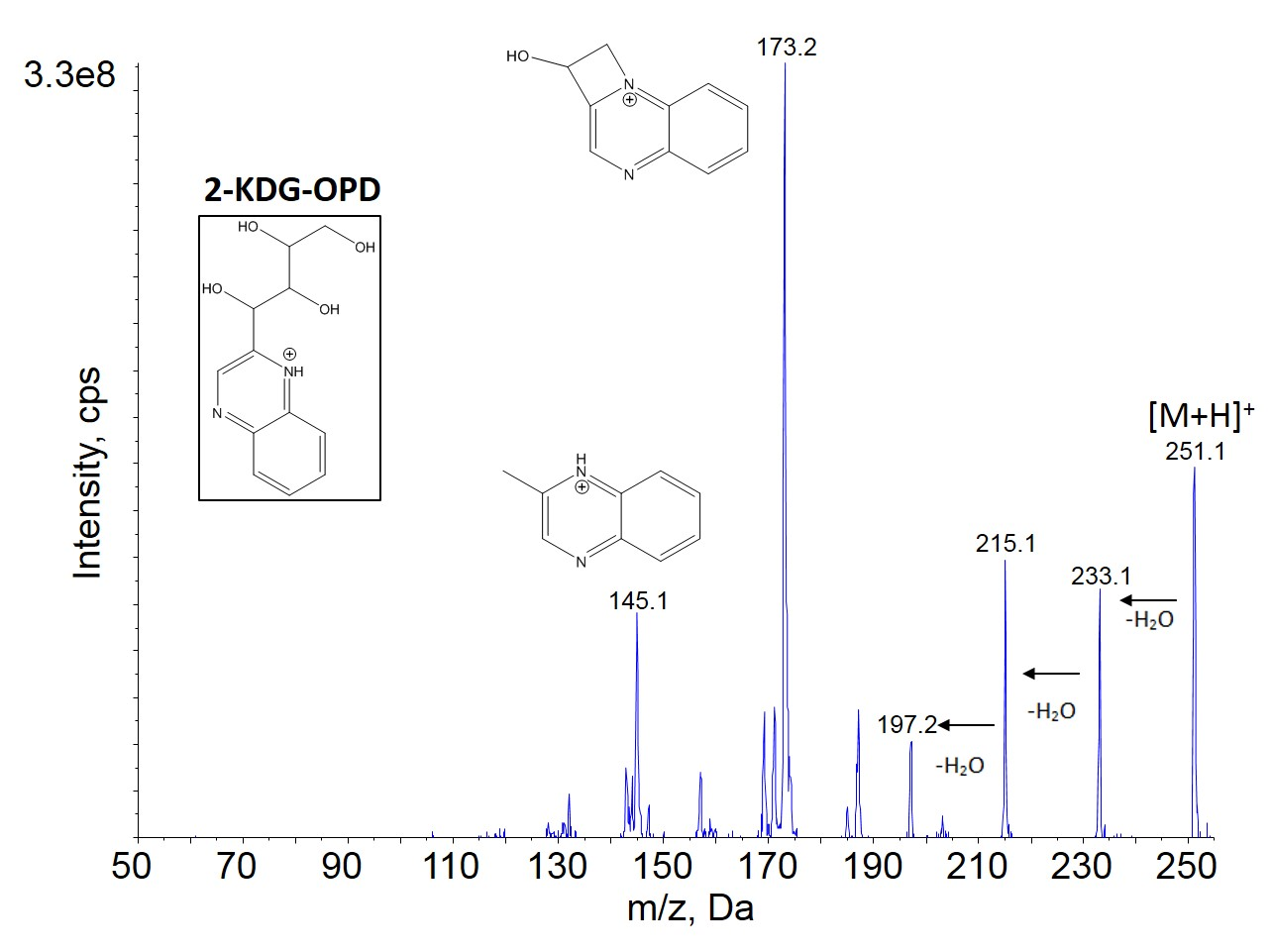

Supplement: S8 Fig — (TIFF) [file pone.0253811.s010.tiff]

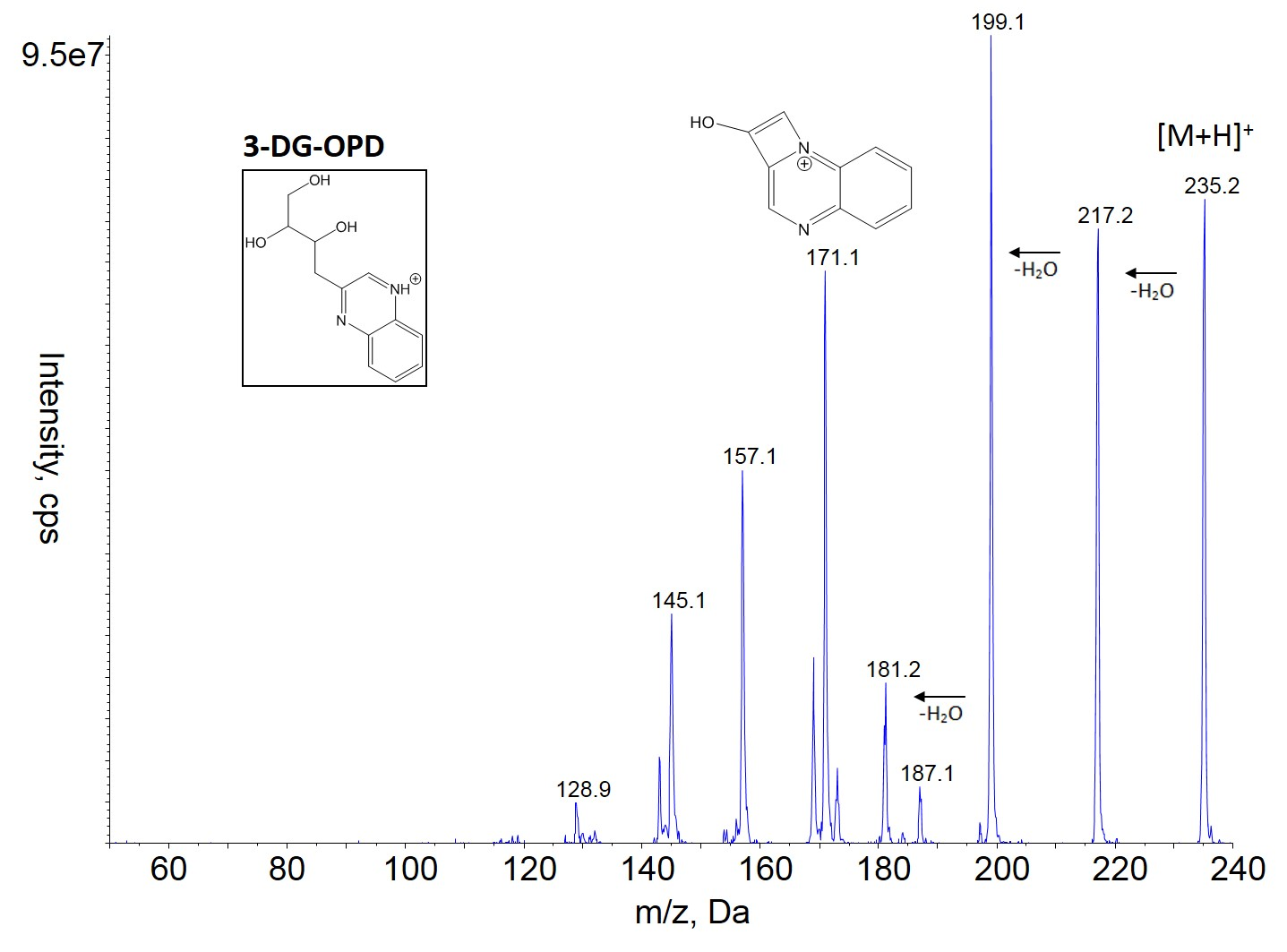

Supplement: S9 Fig — (TIFF) [file pone.0253811.s011.tiff]

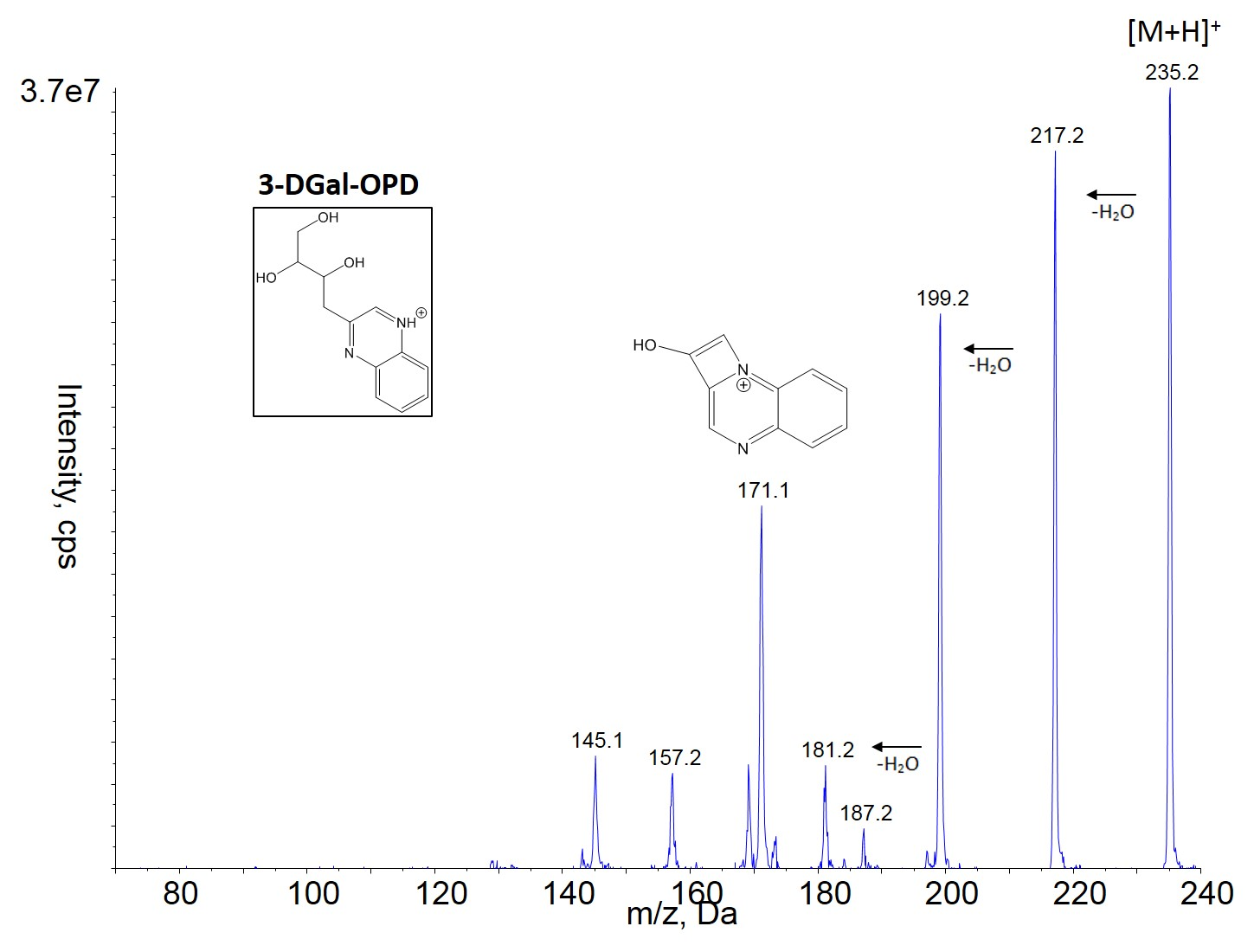

Supplement: S10 Fig — (TIFF) [file pone.0253811.s012.tiff]

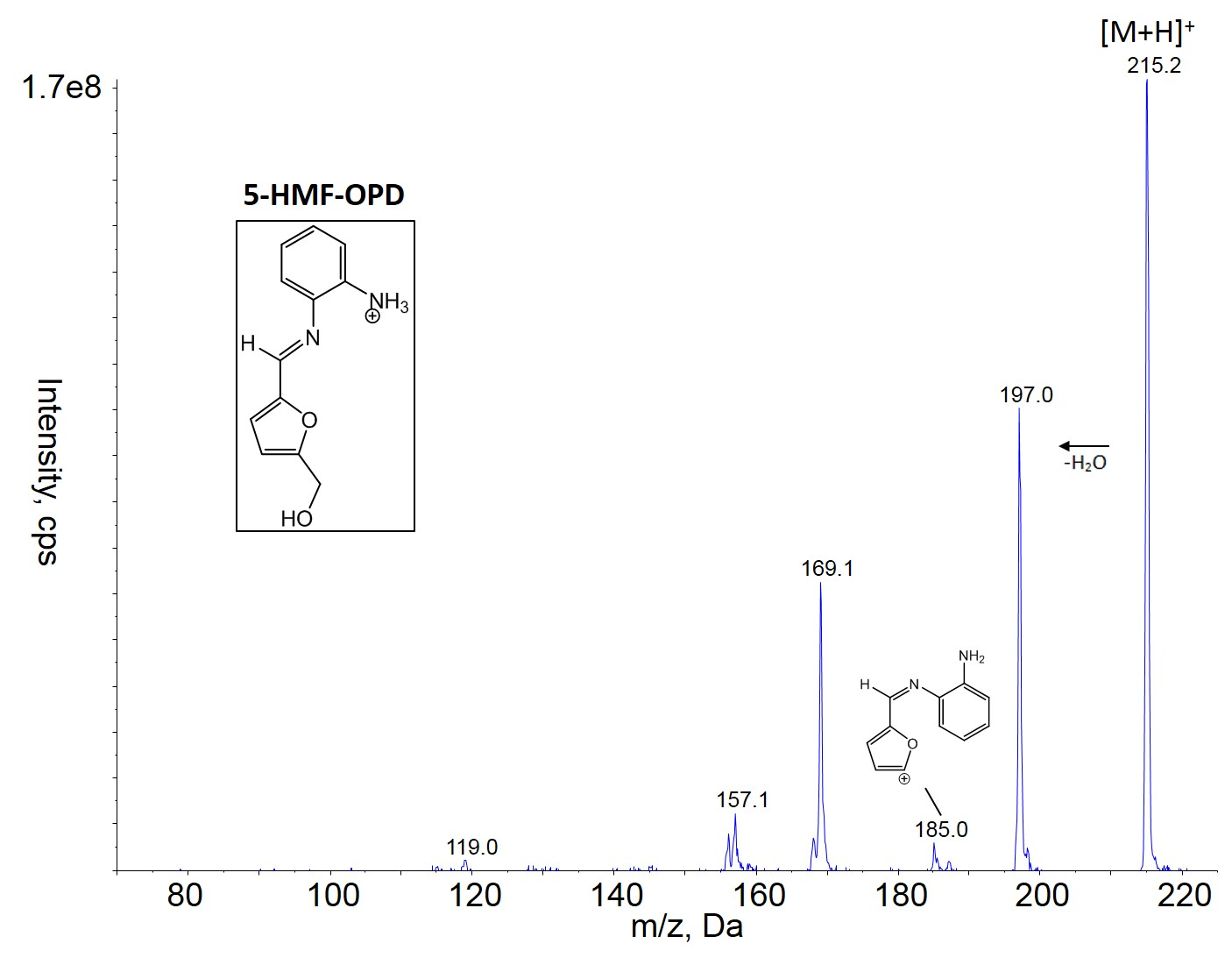

Supplement: S11 Fig — (TIFF) [file pone.0253811.s013.tiff]

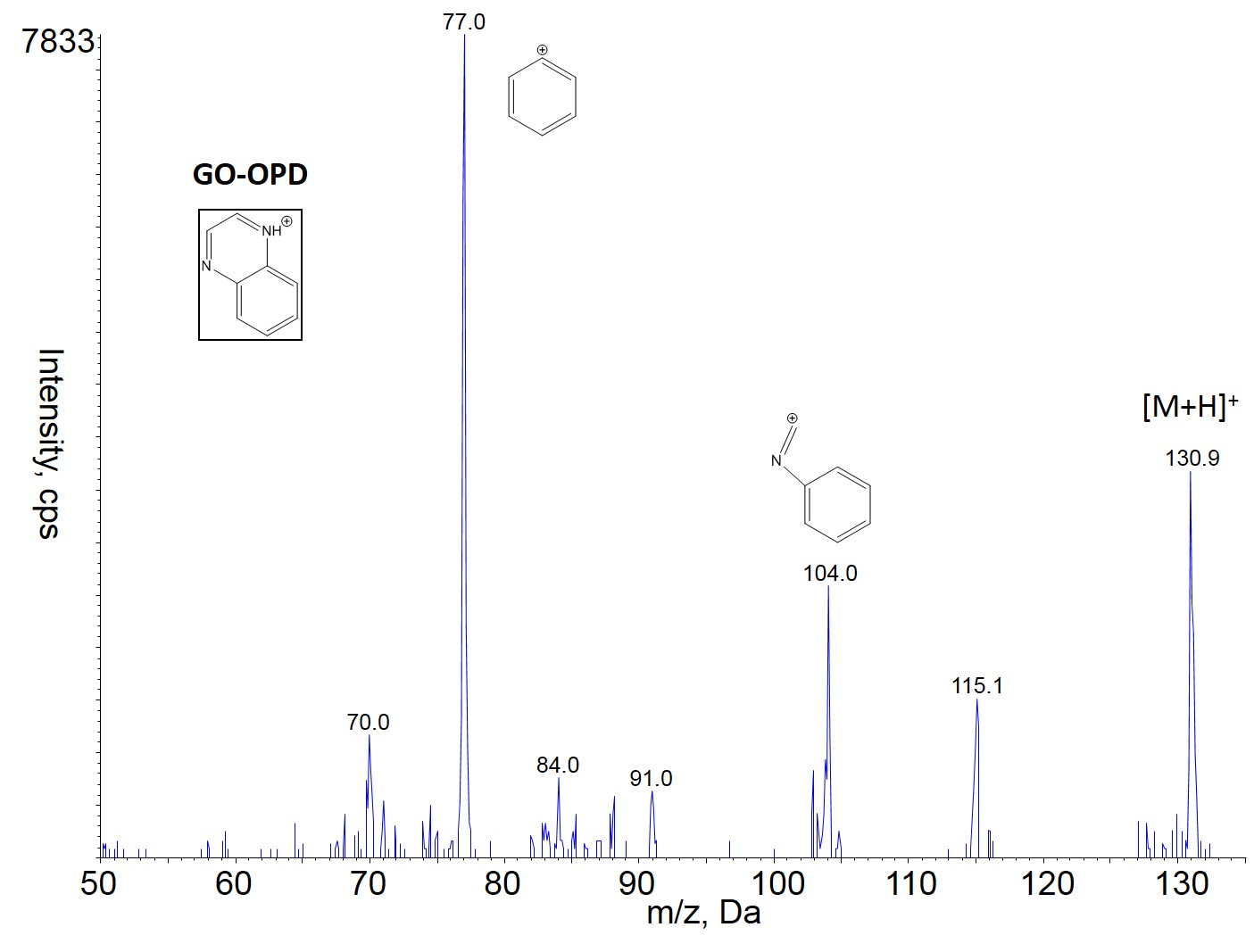

Supplement: S12 Fig — (TIFF) [file pone.0253811.s014.tiff]

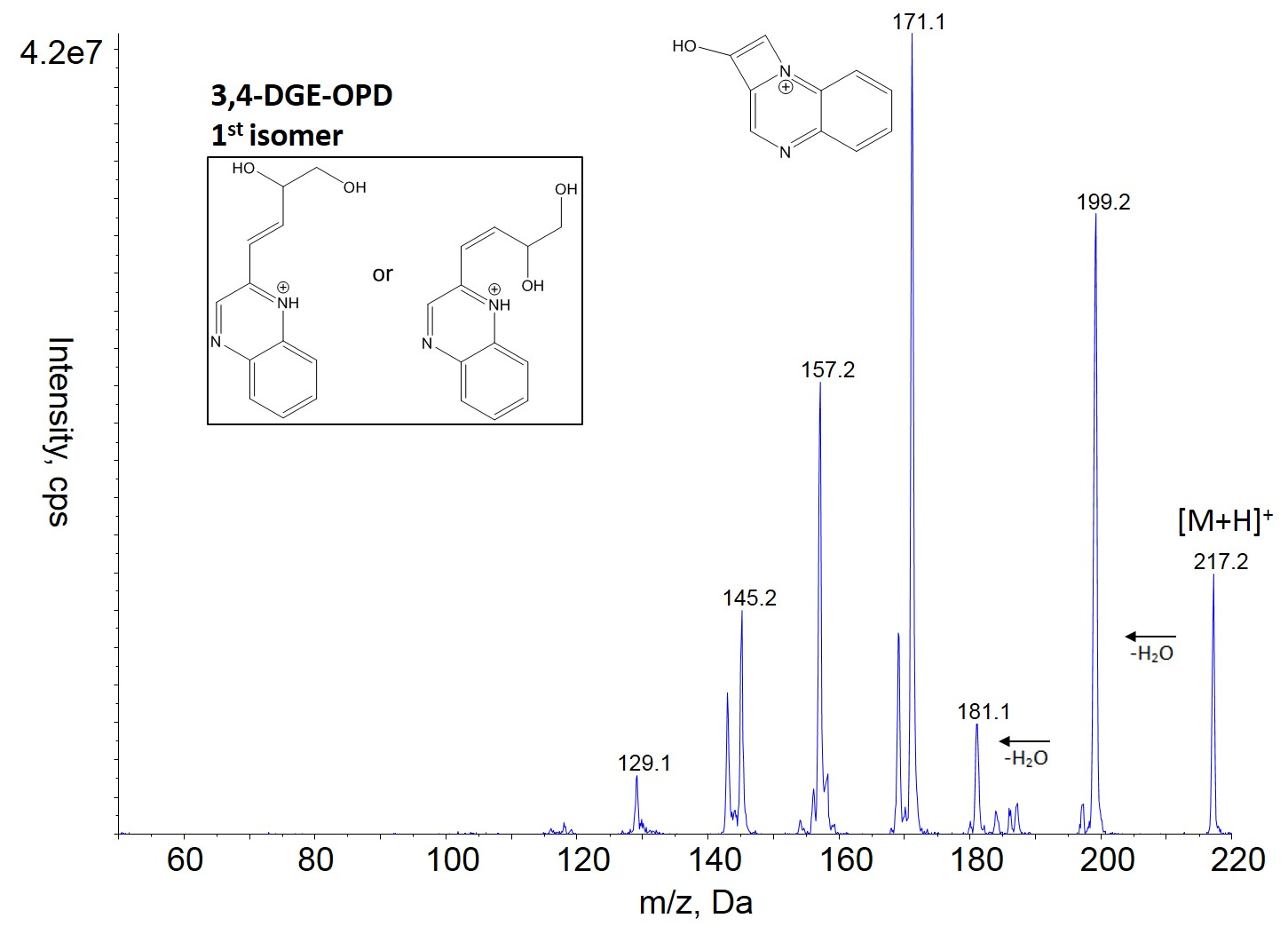

Supplement: S13 Fig — (TIFF) [file pone.0253811.s015.tiff]

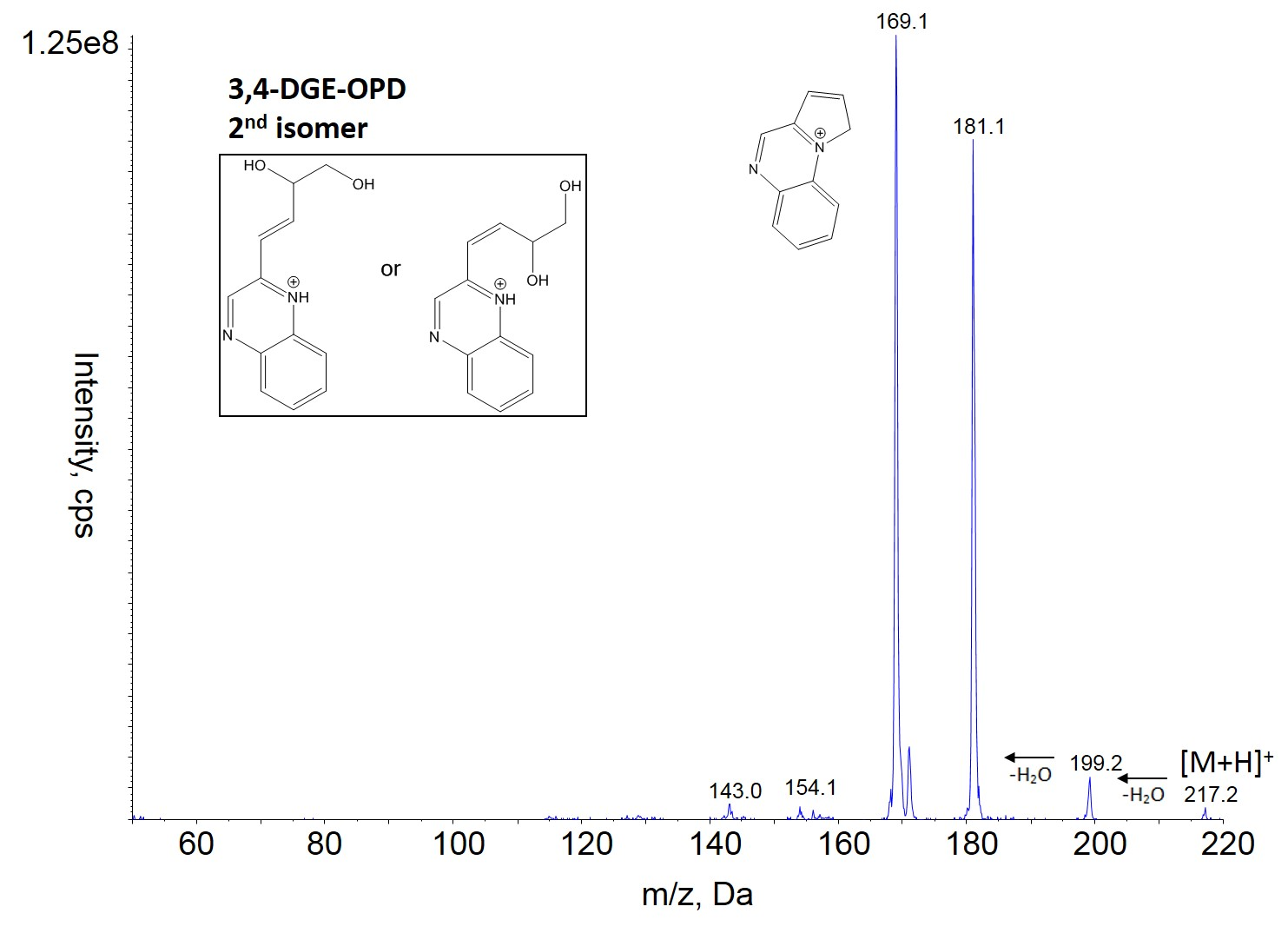

Supplement: S14 Fig — (TIFF) [file pone.0253811.s016.tiff]

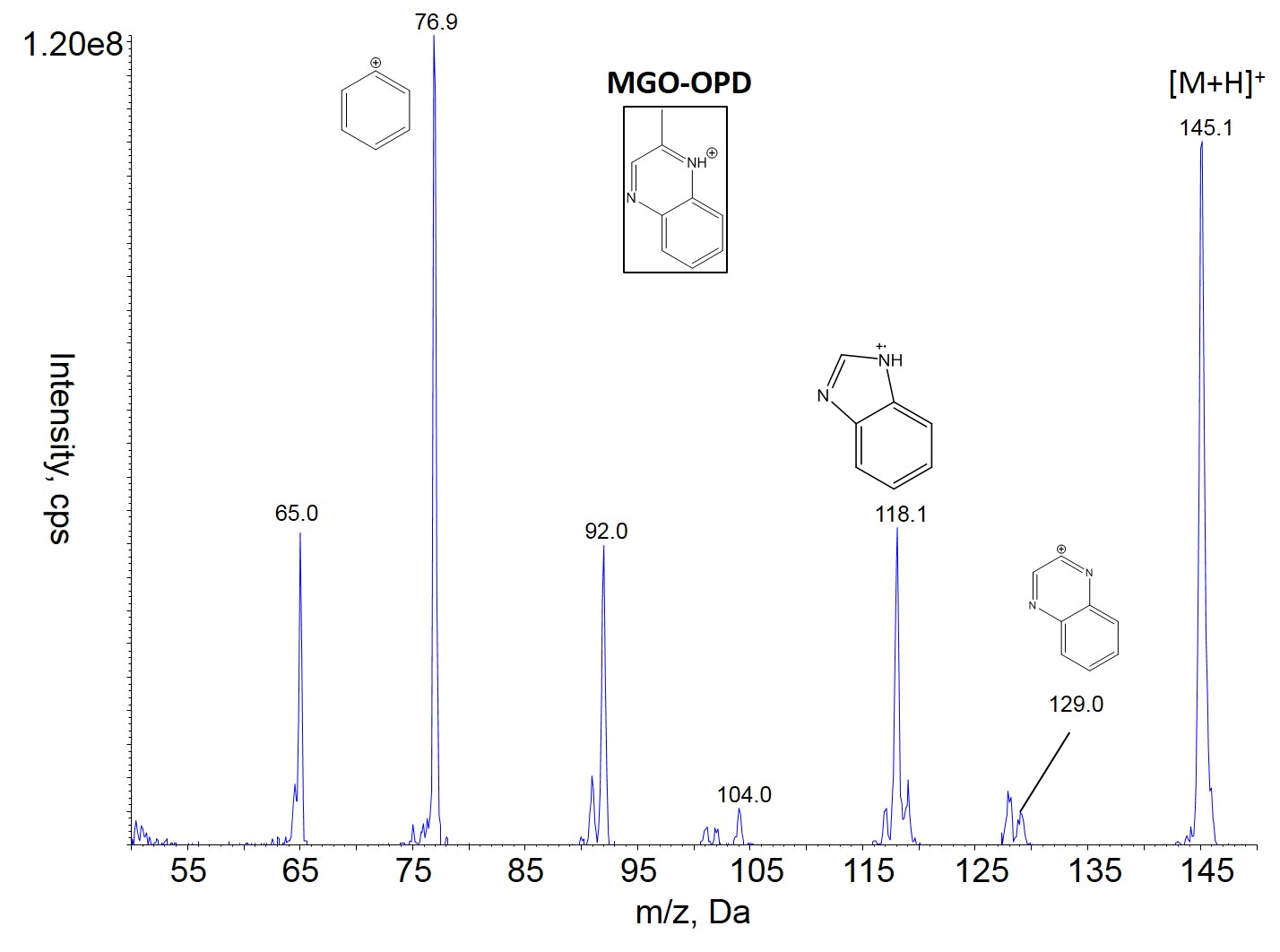

Supplement: S15 Fig — (TIFF) [file pone.0253811.s017.tiff]

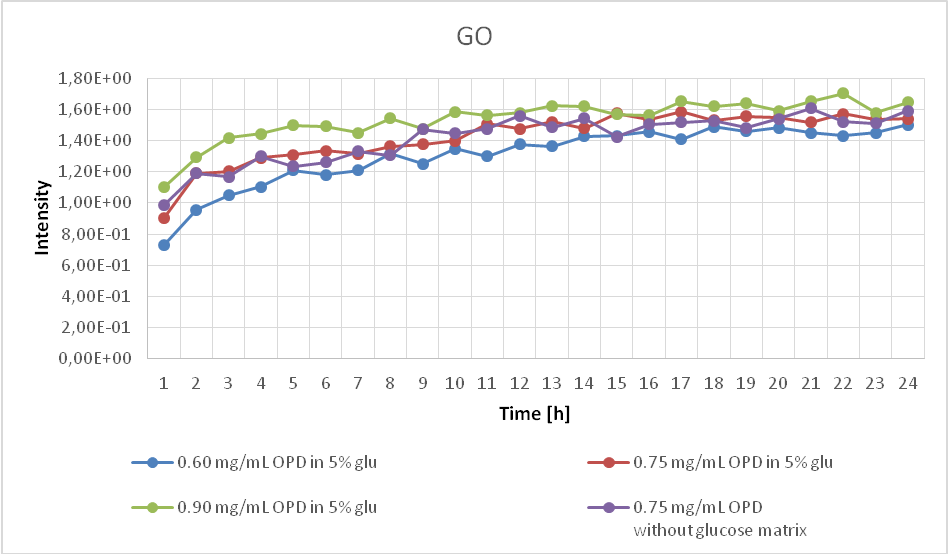

Supplement: S16 Fig — (TIFF) [file pone.0253811.s018.tiff]

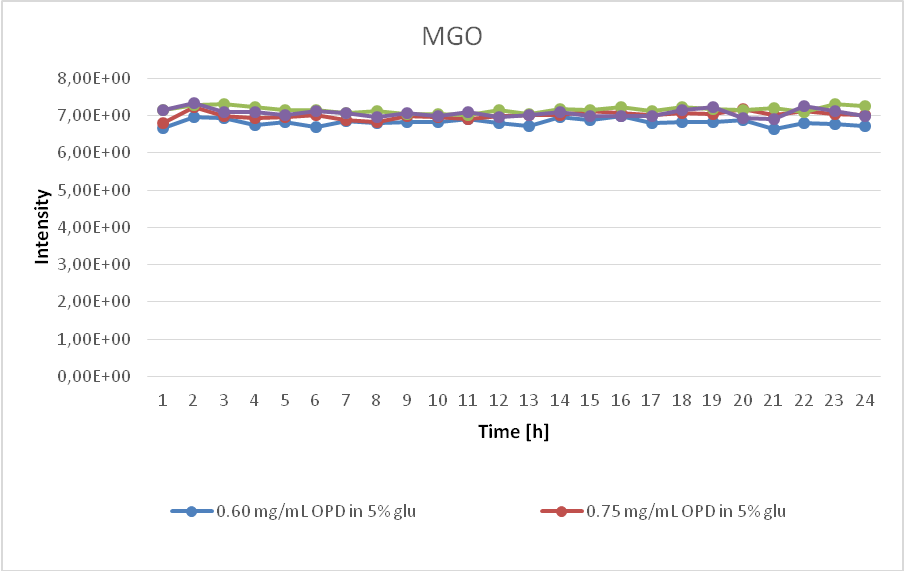

Supplement: S17 Fig — (TIFF) [file pone.0253811.s019.tiff]

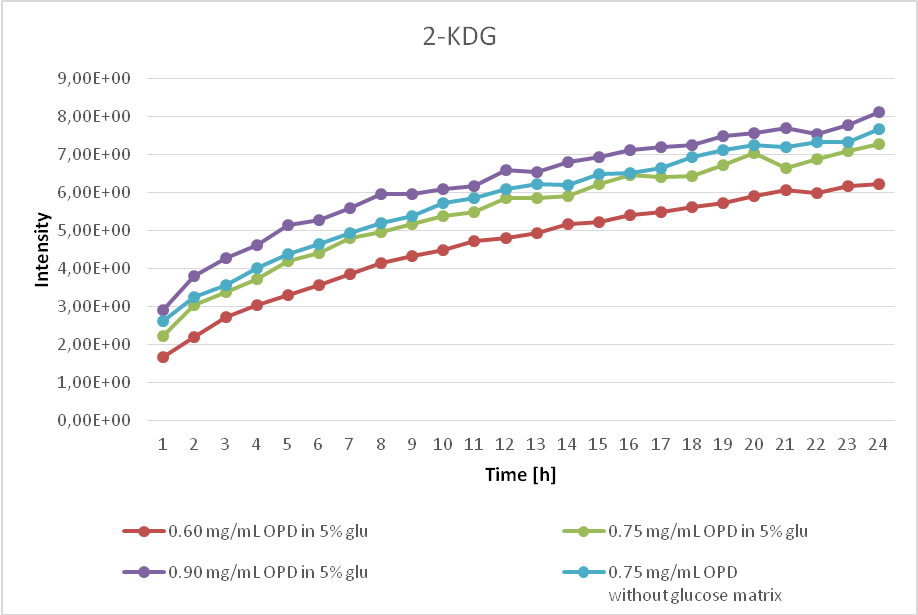

Supplement: S18 Fig — (TIFF) [file pone.0253811.s020.tiff]

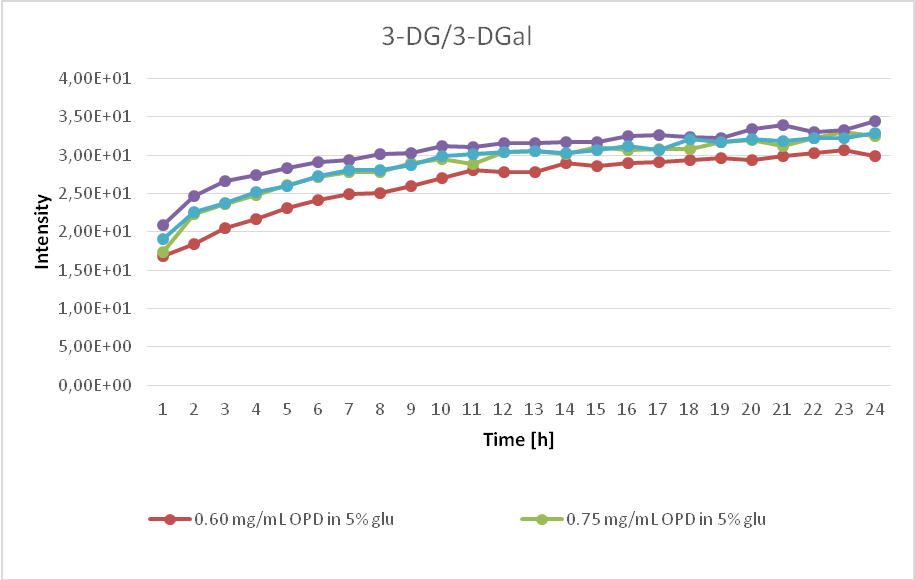

Supplement: S19 Fig — (TIFF) [file pone.0253811.s021.tiff]

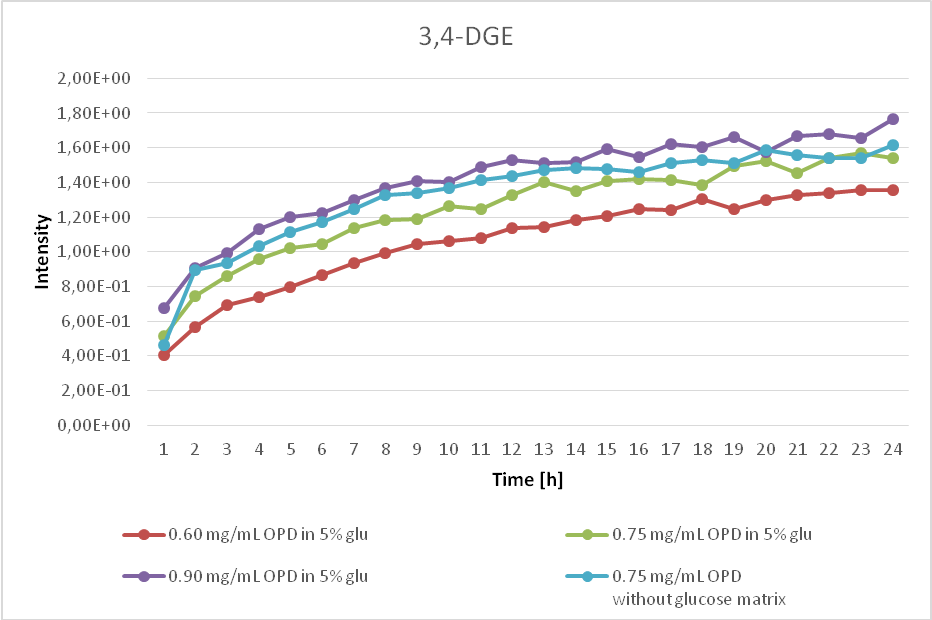

Supplement: S20 Fig — (TIFF) [file pone.0253811.s022.tiff]

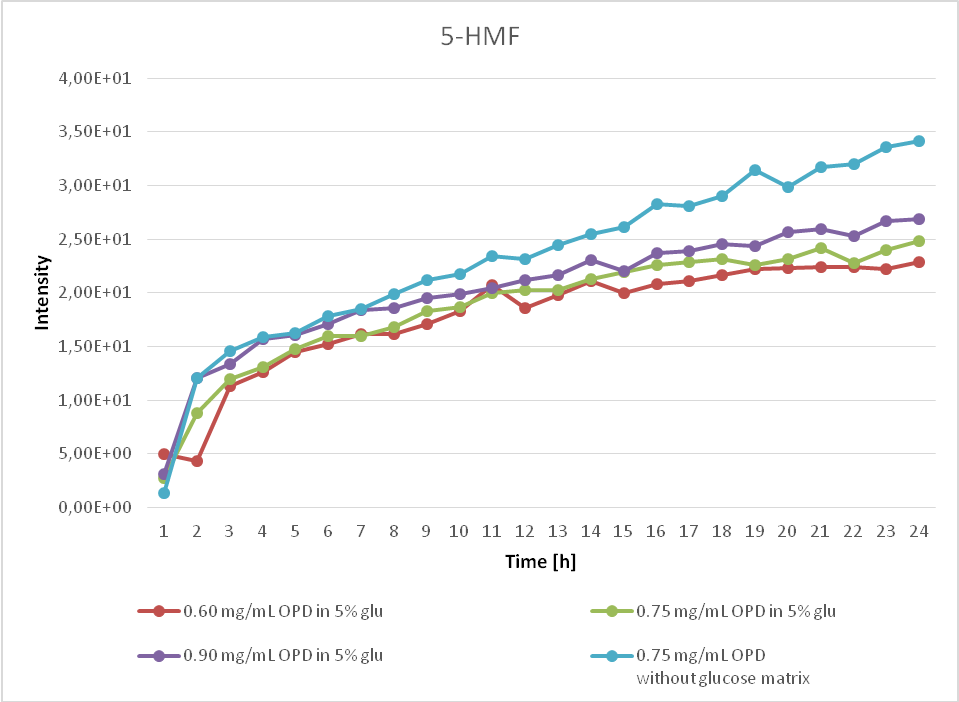

Supplement: S21 Fig — (TIFF) [file pone.0253811.s023.tiff]

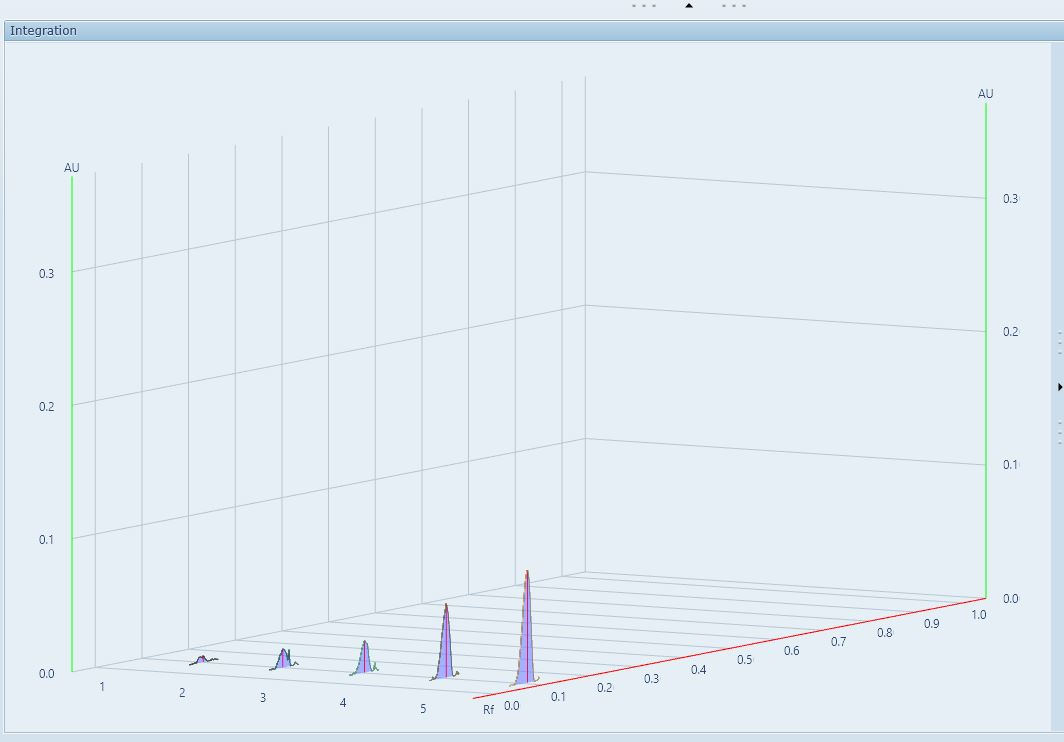

Supplement: S22 Fig — (TIFF) [file pone.0253811.s024.tiff]

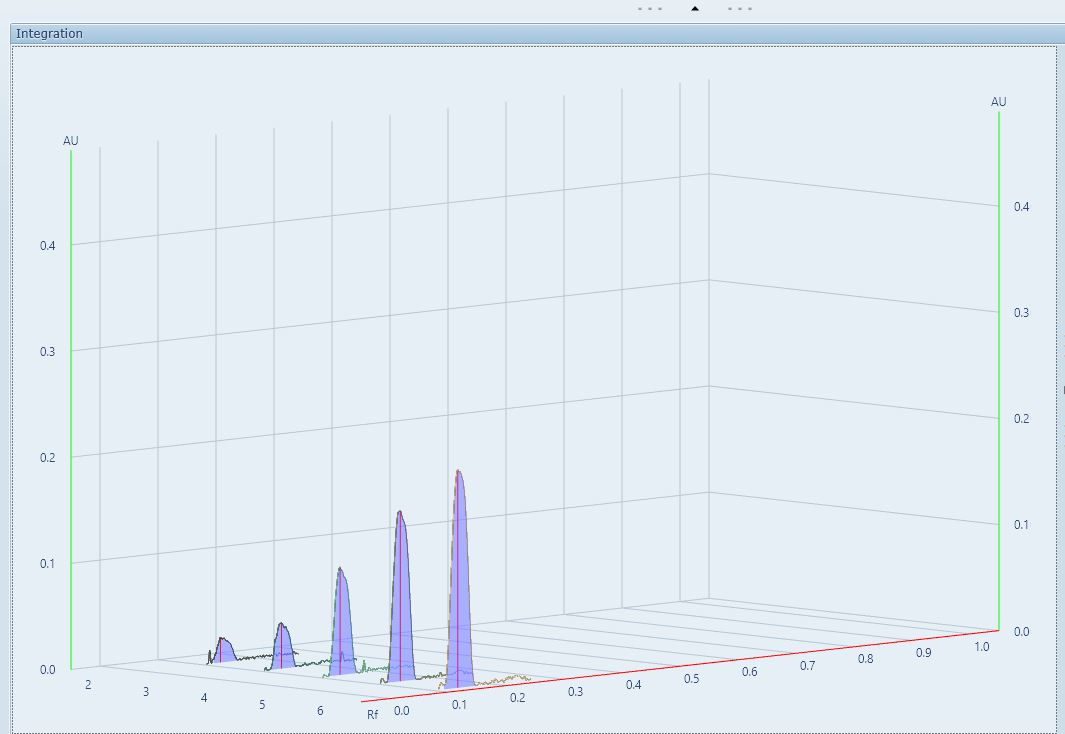

Supplement: S23 Fig — (TIFF) [file pone.0253811.s025.tiff]

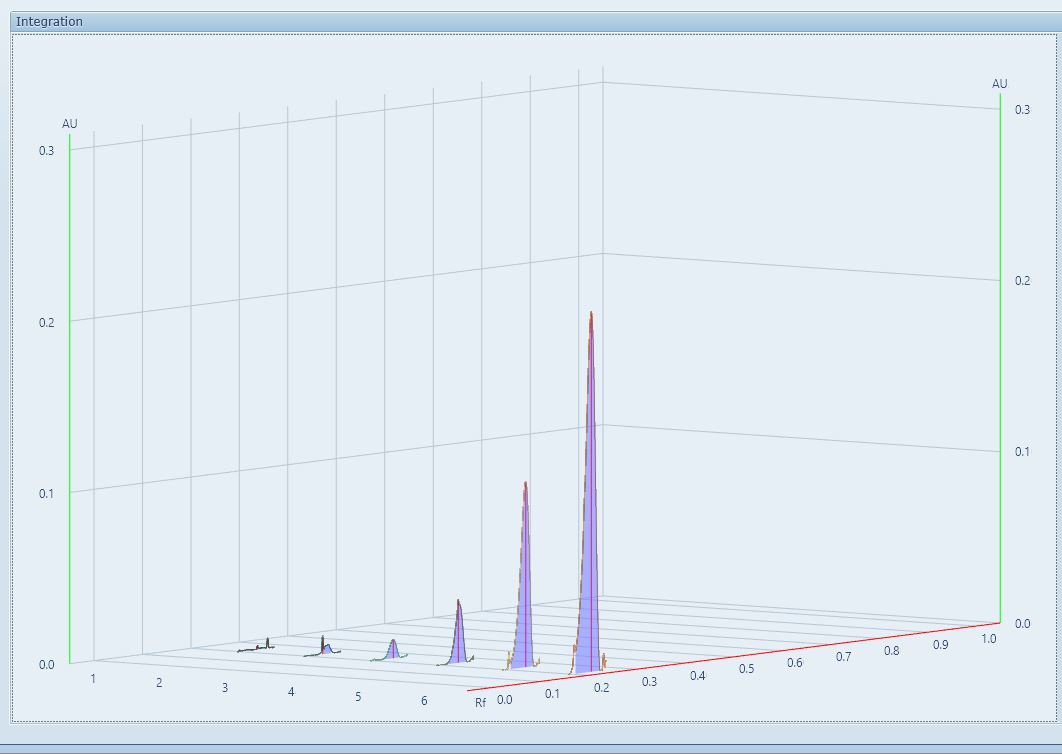

Supplement: S24 Fig — (TIFF) [file pone.0253811.s026.tiff]

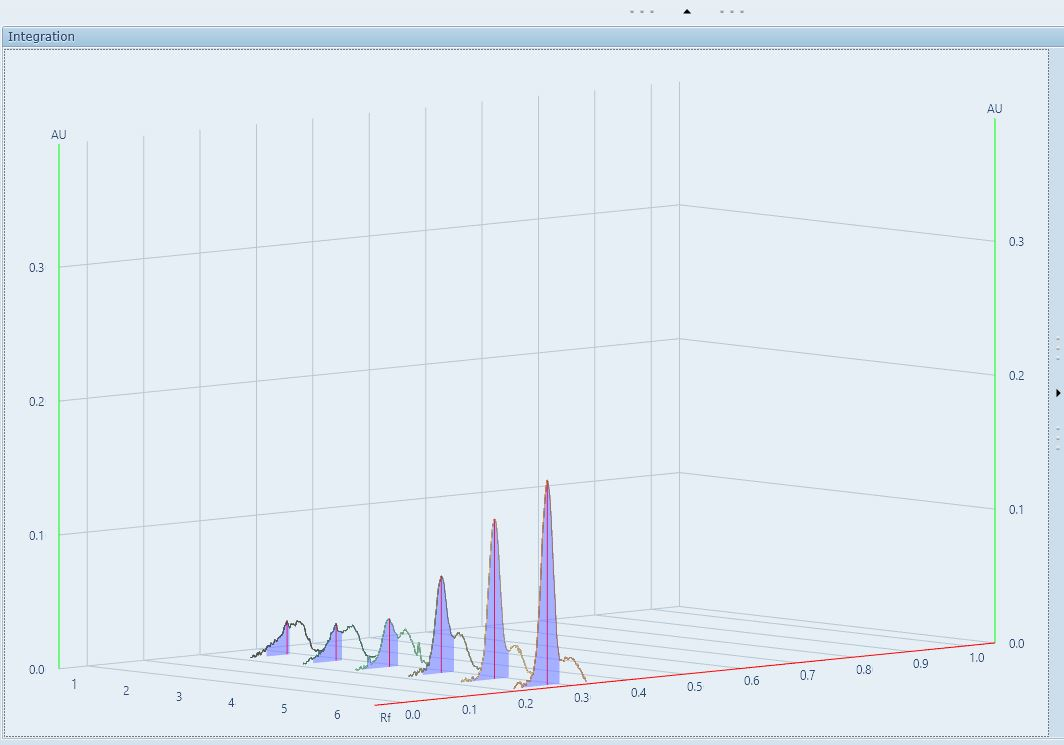

Supplement: S25 Fig — (TIFF) [file pone.0253811.s027.tiff]

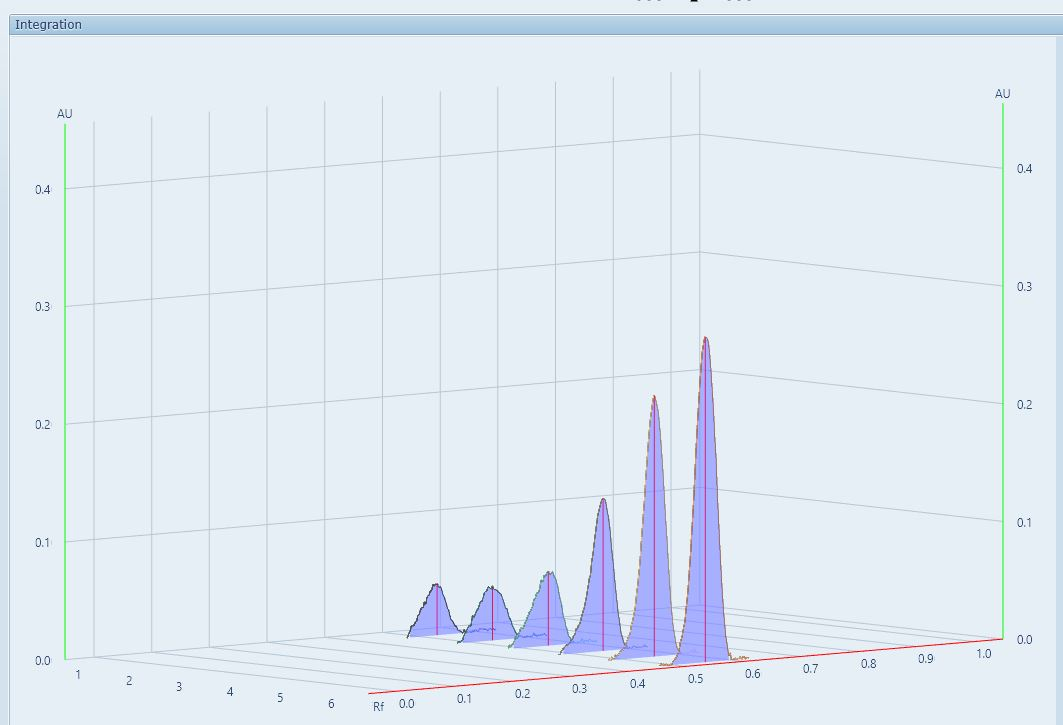

Supplement: S26 Fig — (TIFF) [file pone.0253811.s028.tiff]

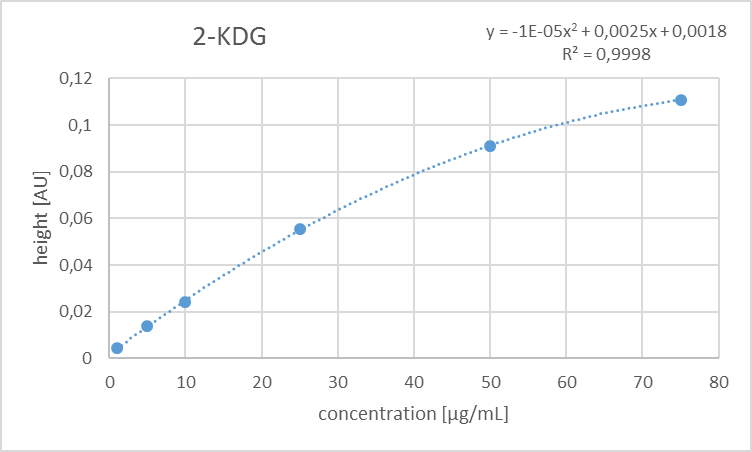

Supplement: S27 Fig — (TIFF) [file pone.0253811.s029.tiff]

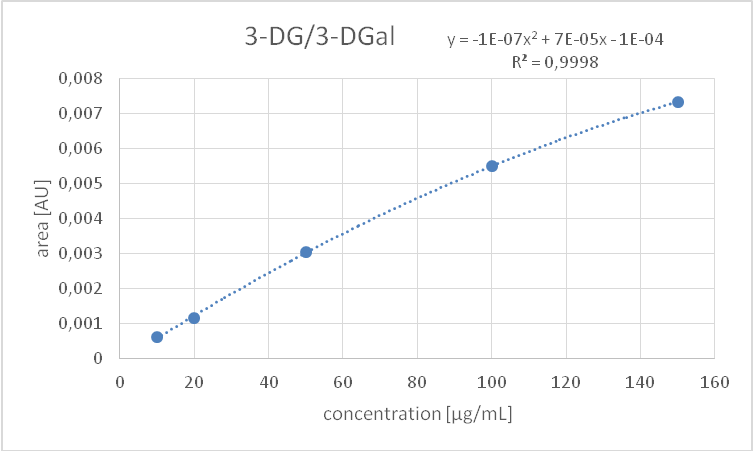

Supplement: S28 Fig — (TIFF) [file pone.0253811.s030.tiff]

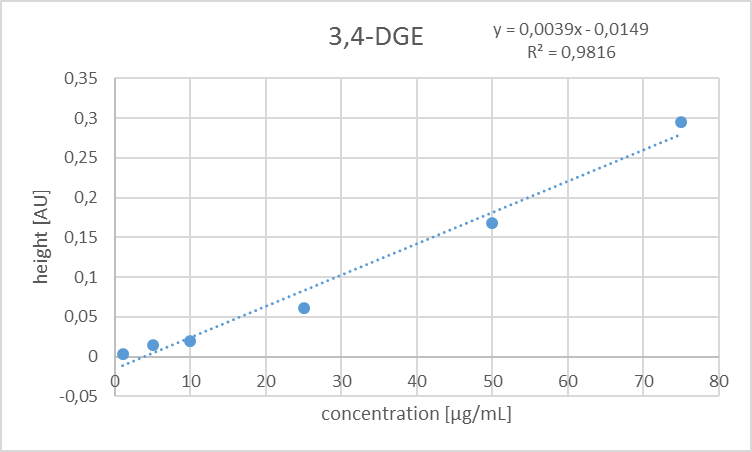

Supplement: S29 Fig — (TIFF) [file pone.0253811.s031.tiff]

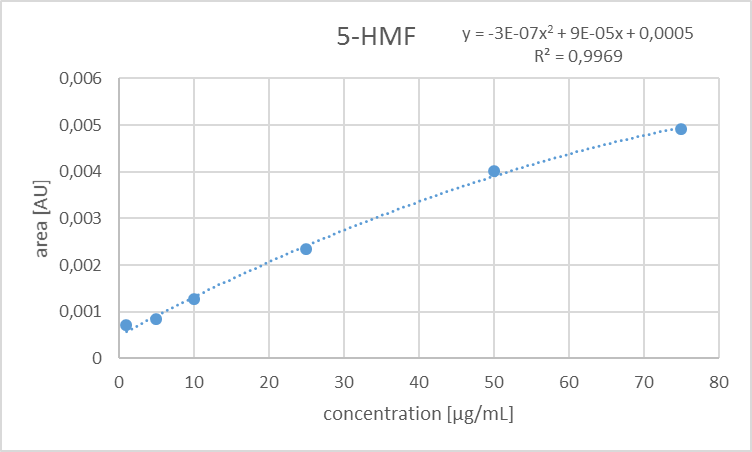

Supplement: S30 Fig — (TIFF) [file pone.0253811.s032.tiff]

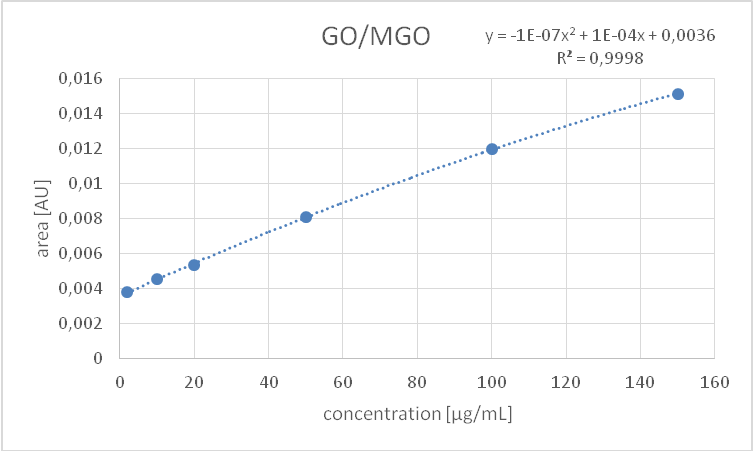

Supplement: S31 Fig — (TIFF) [file pone.0253811.s033.tiff]

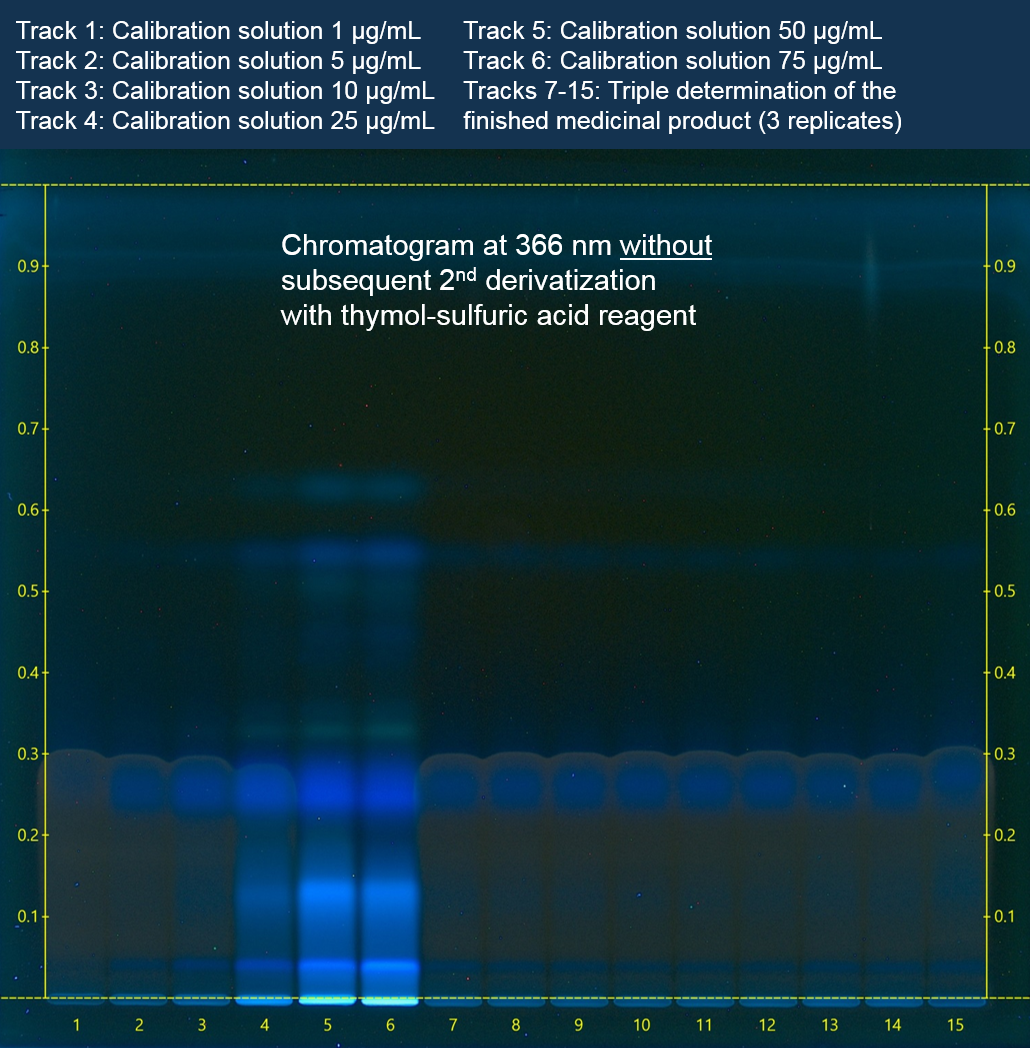

Supplement: S32 Fig — (TIFF) [file pone.0253811.s034.tiff]

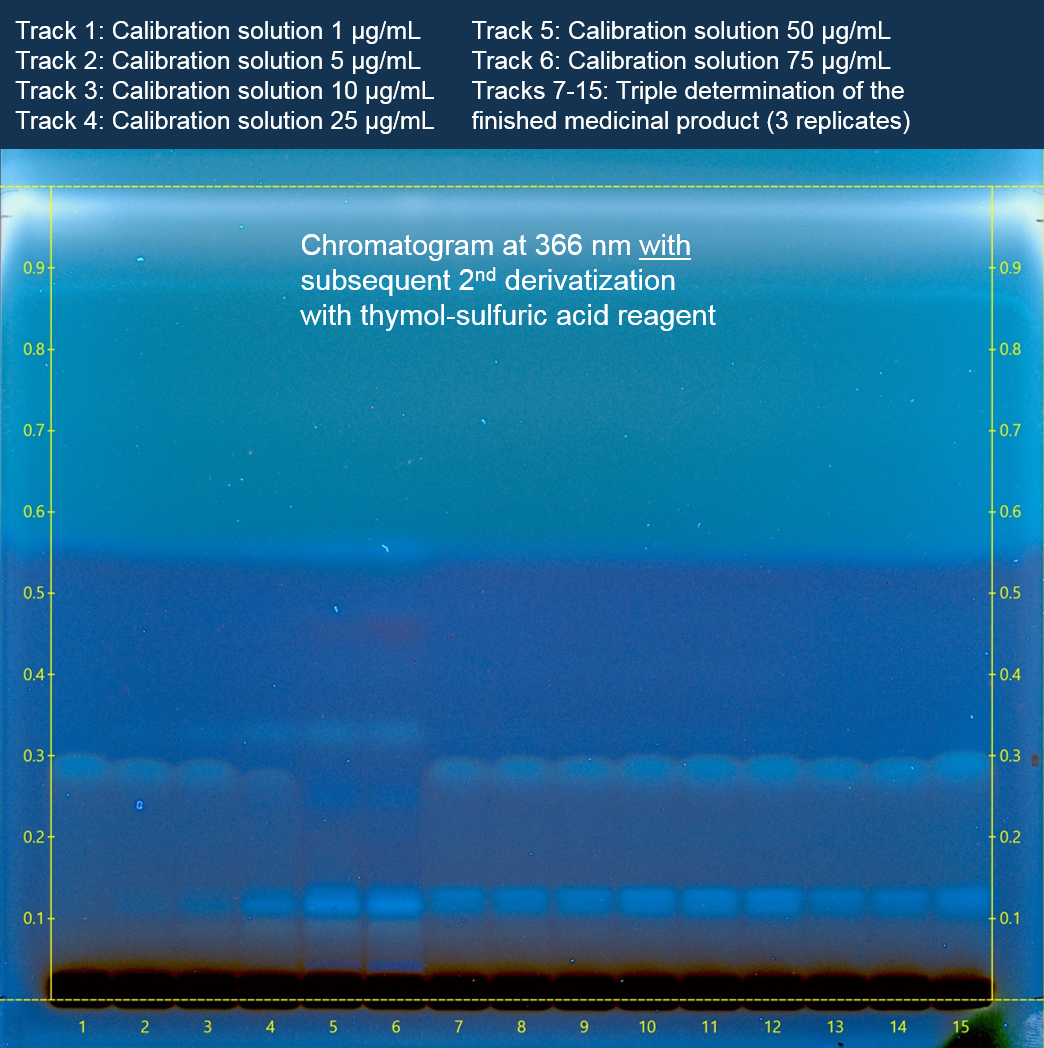

Supplement: S33 Fig — (TIFF) [file pone.0253811.s035.tiff]

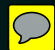

2015-01-25 10:10:10

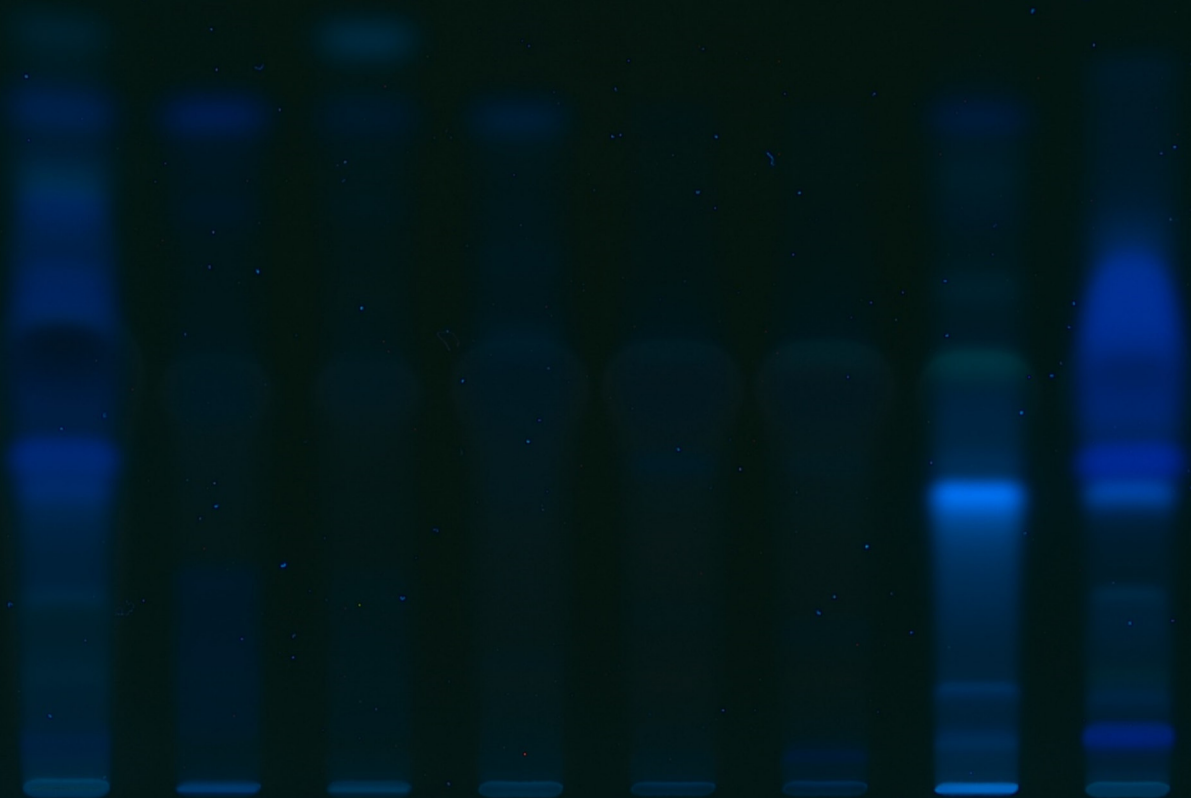

25/5/20 0.5ug/ml + M2

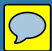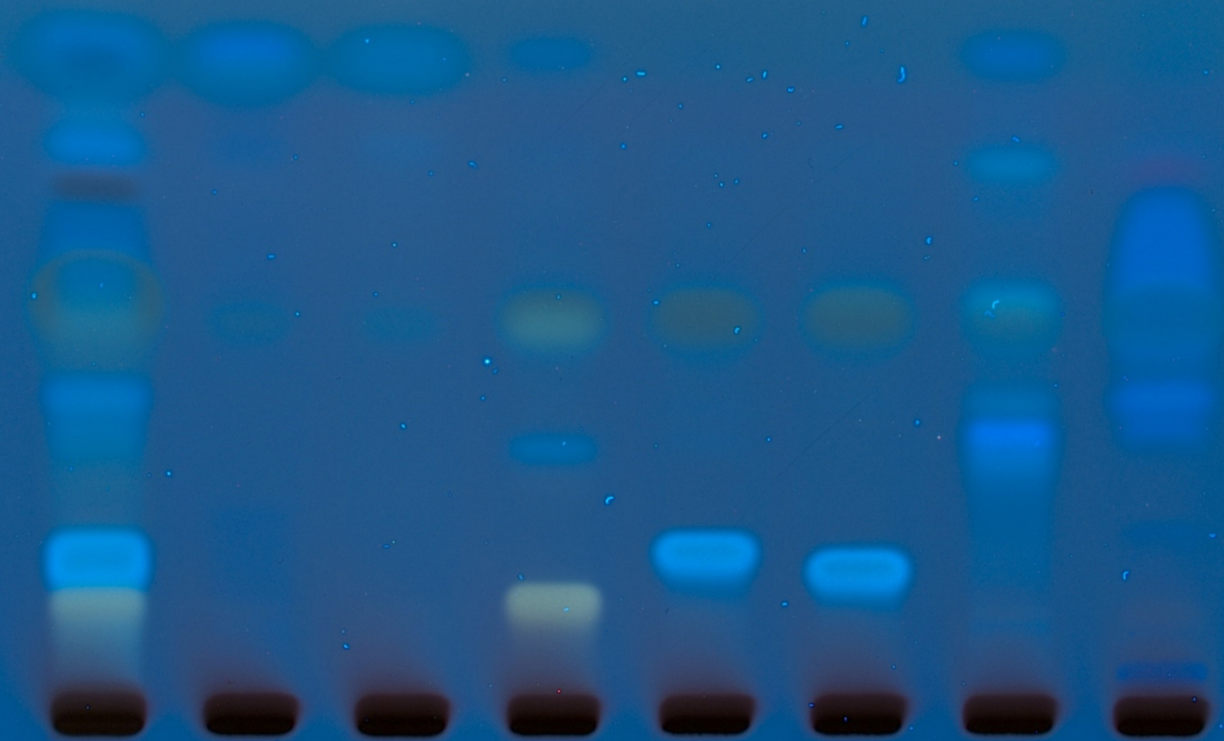

25/5/20 Kali mit Röhre

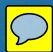

MeOH = Ethylacetat 15/11/19  
30 70

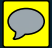

KeOH: JCM 14/11/19  
30 = 70

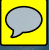

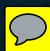

~~5000~~ = 7004 = Total  
50 = 50

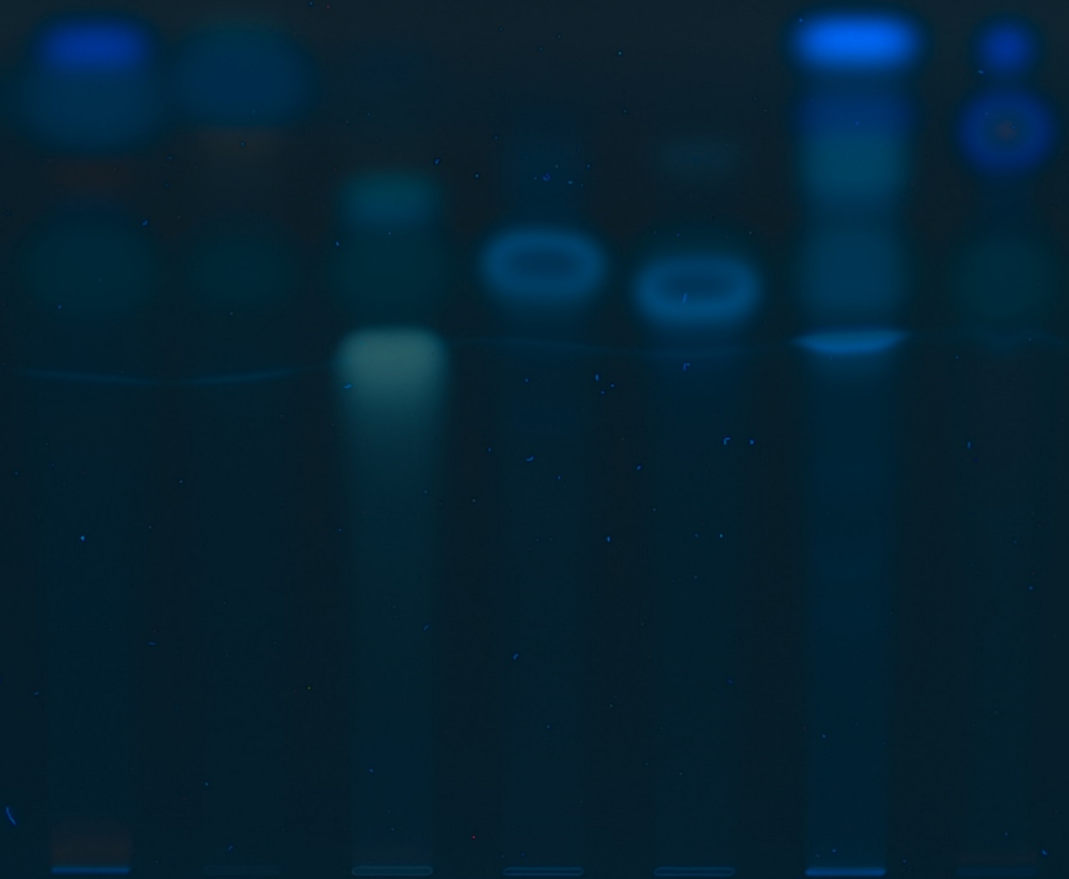

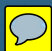

Dox: Tol  
95.5

8/11/11

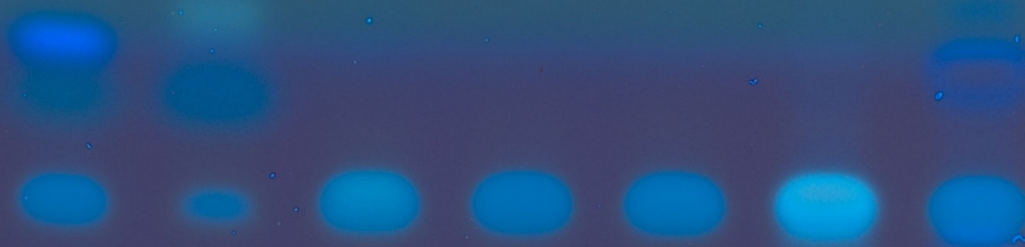

31/10/99 Day 101 - 1000000  
1000000 1000000 1000000

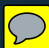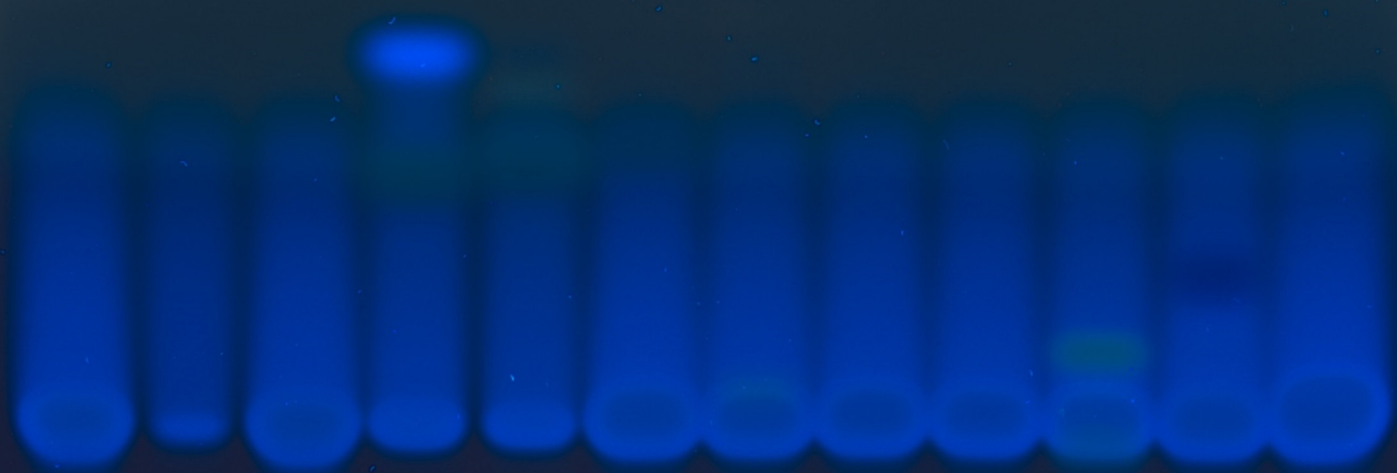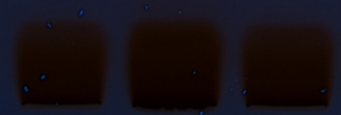

Vgl. Gm-GDP-OLD an 10/01

31/10/19

Dror: Tol: 63

45 45 10

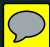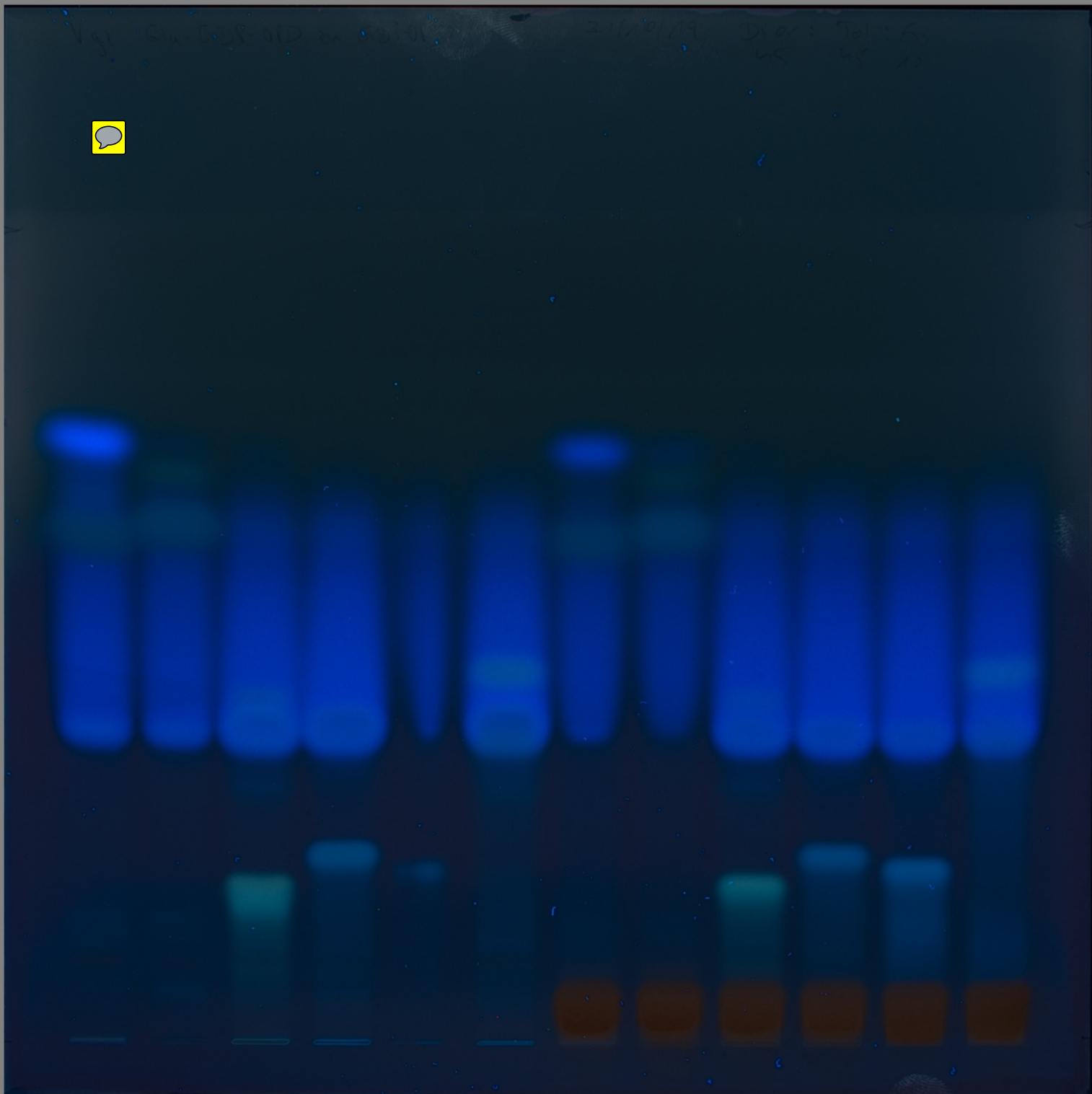

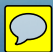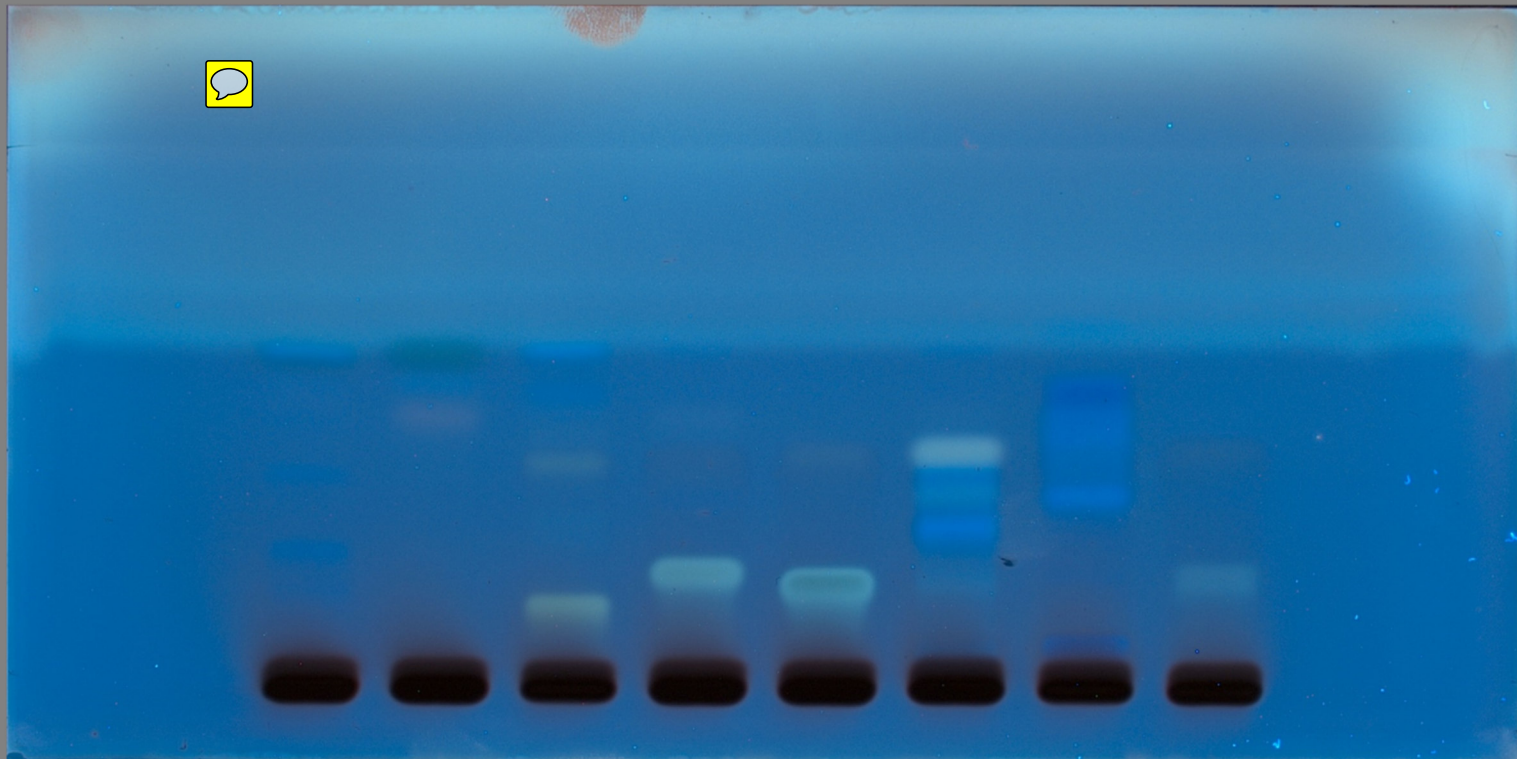

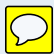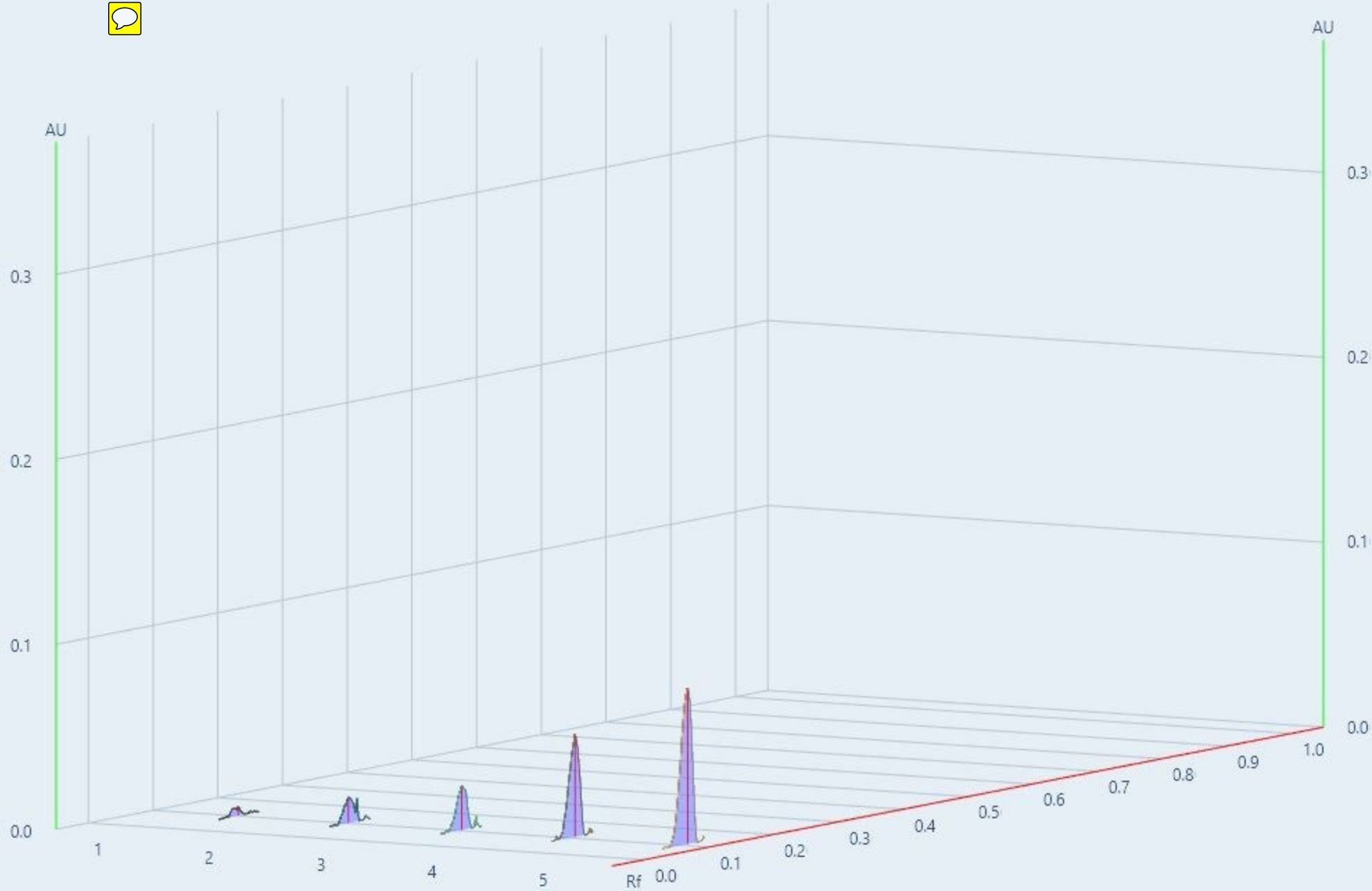

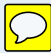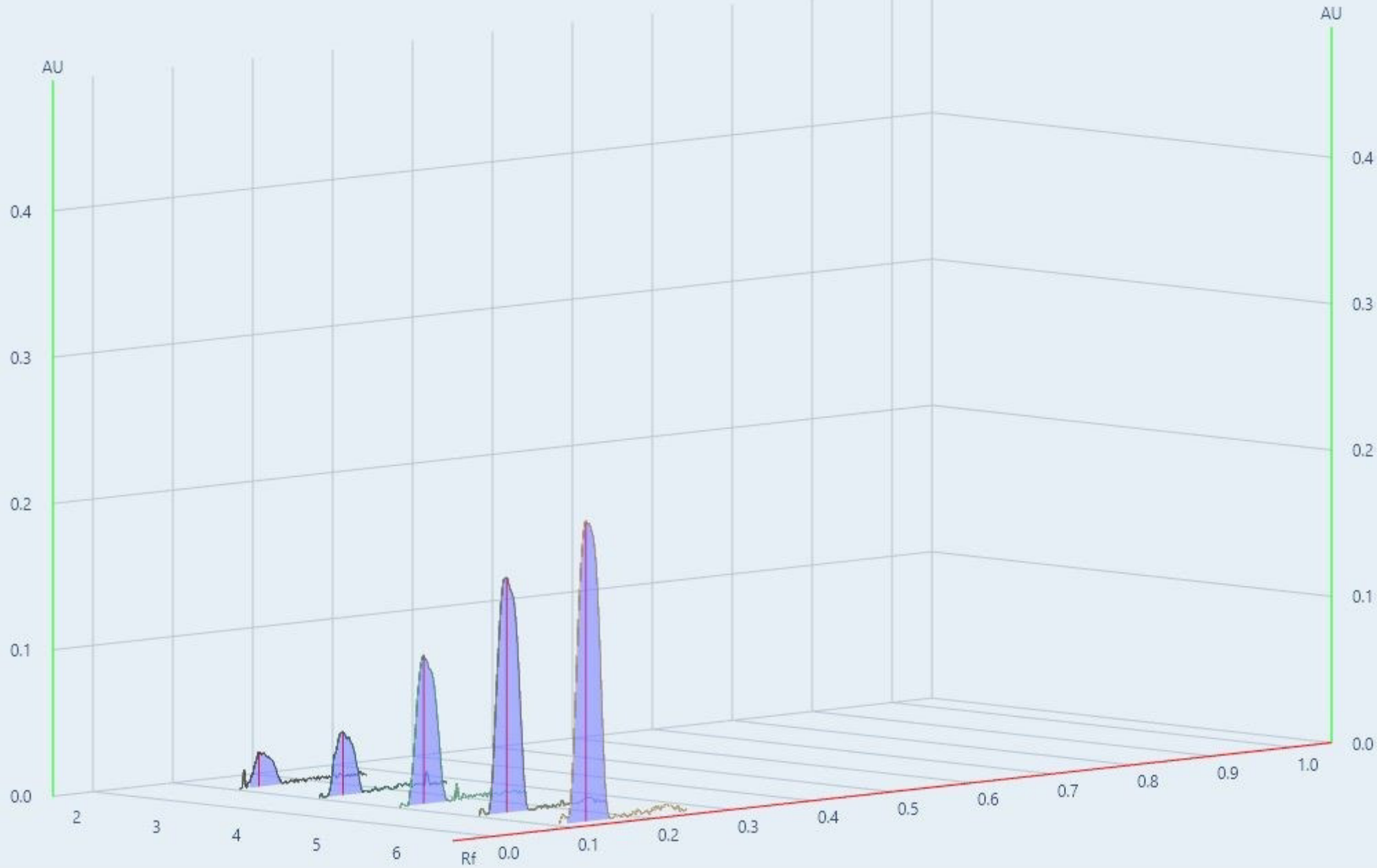

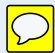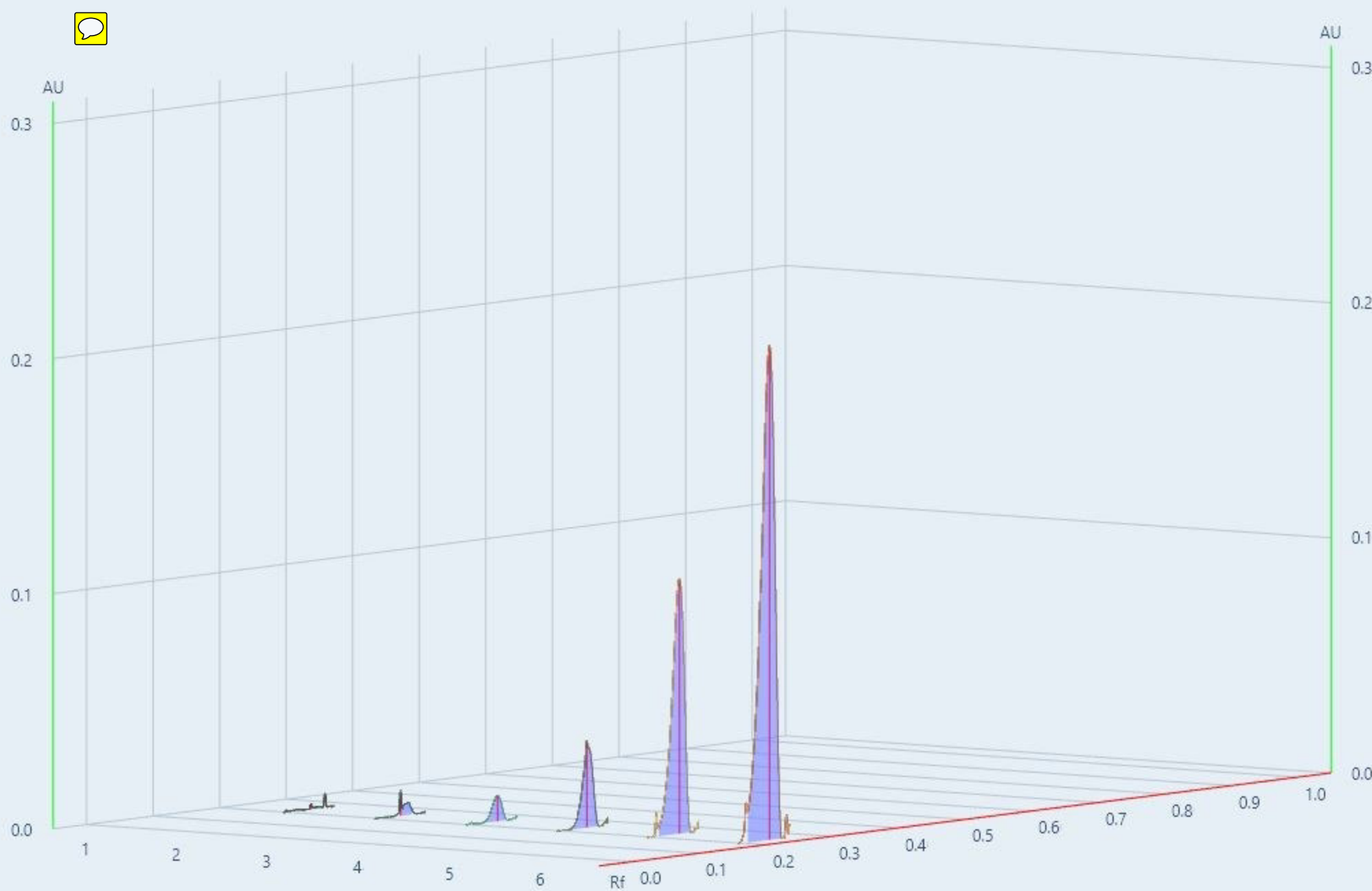

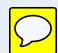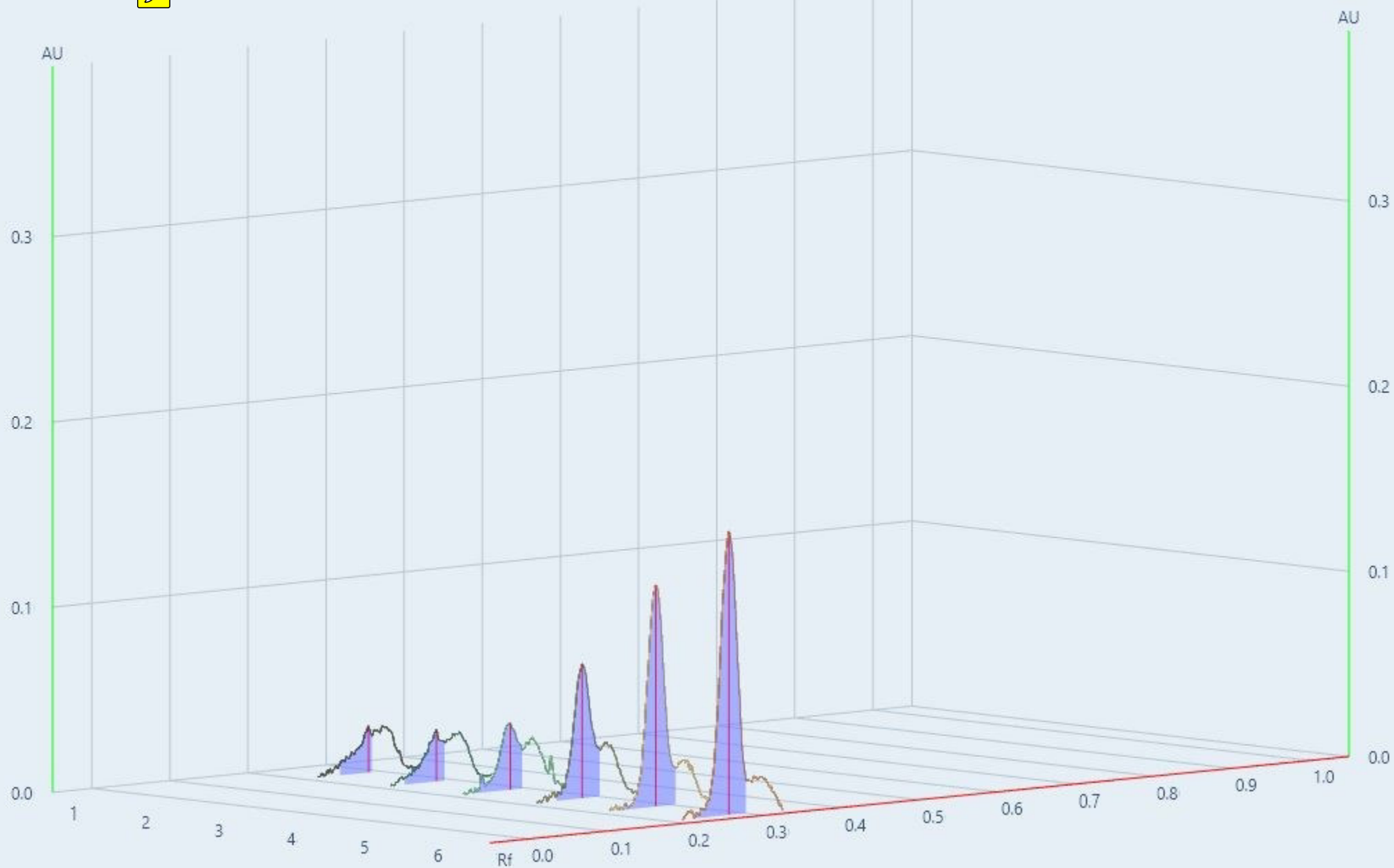

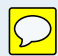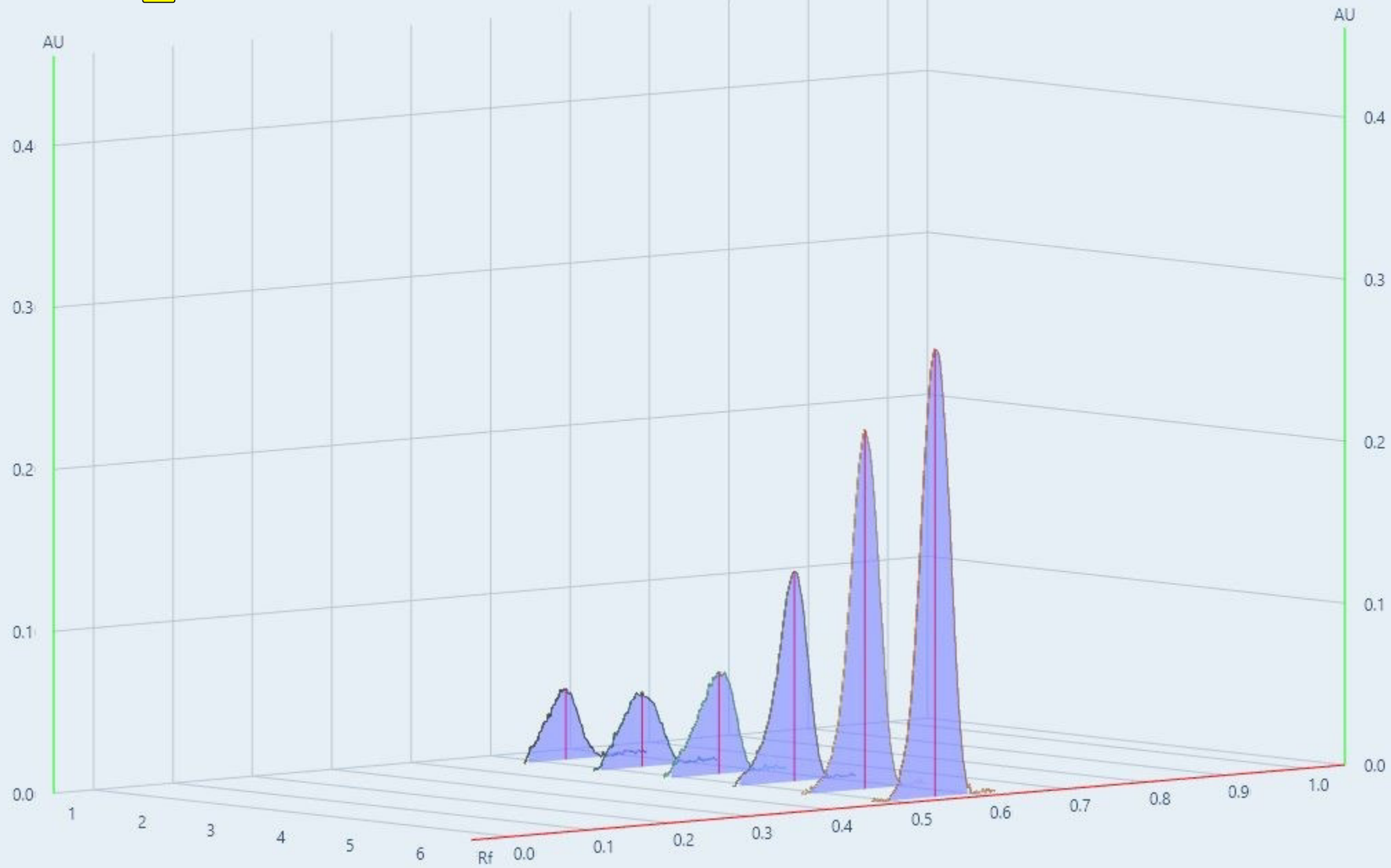

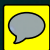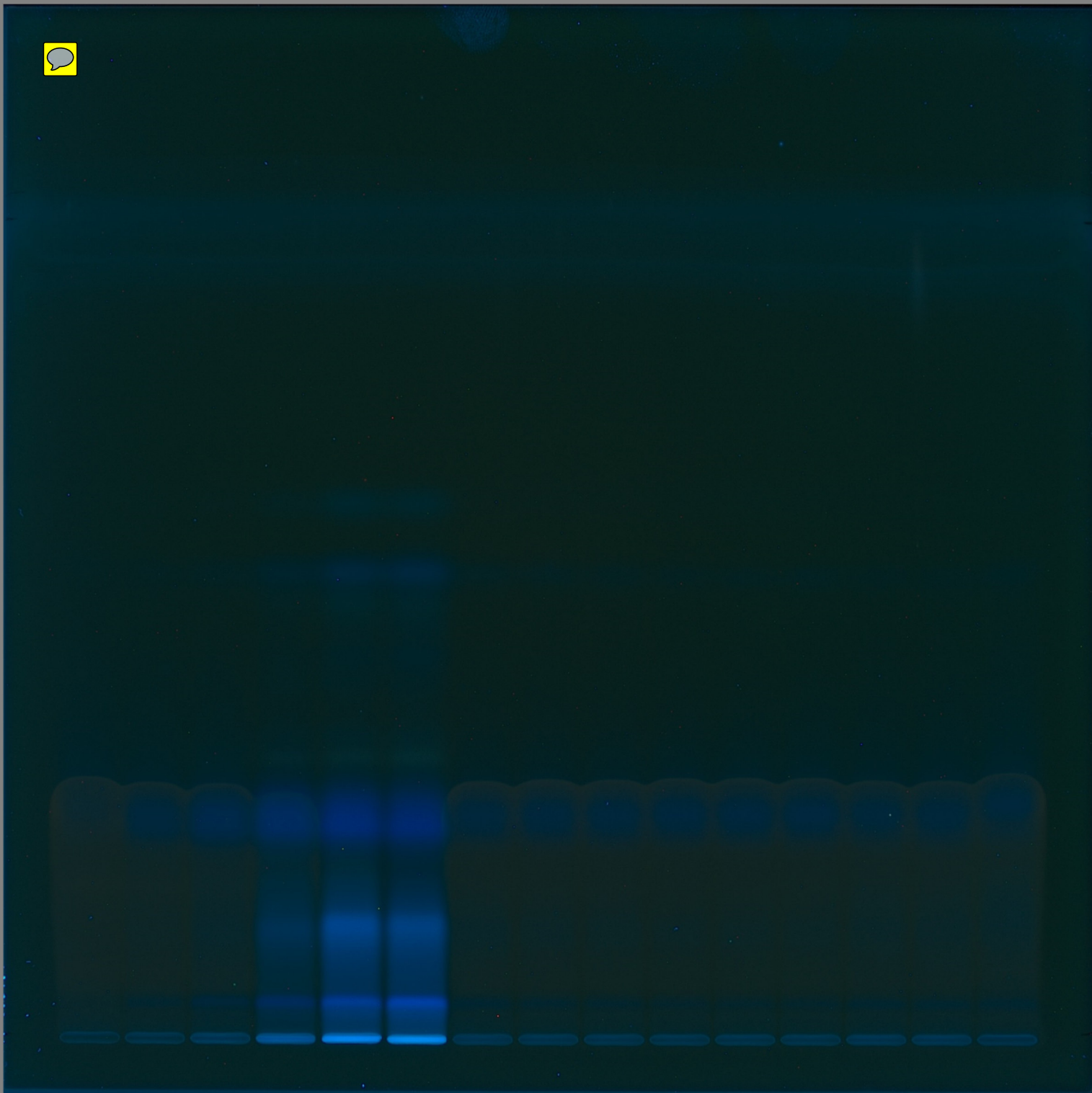

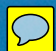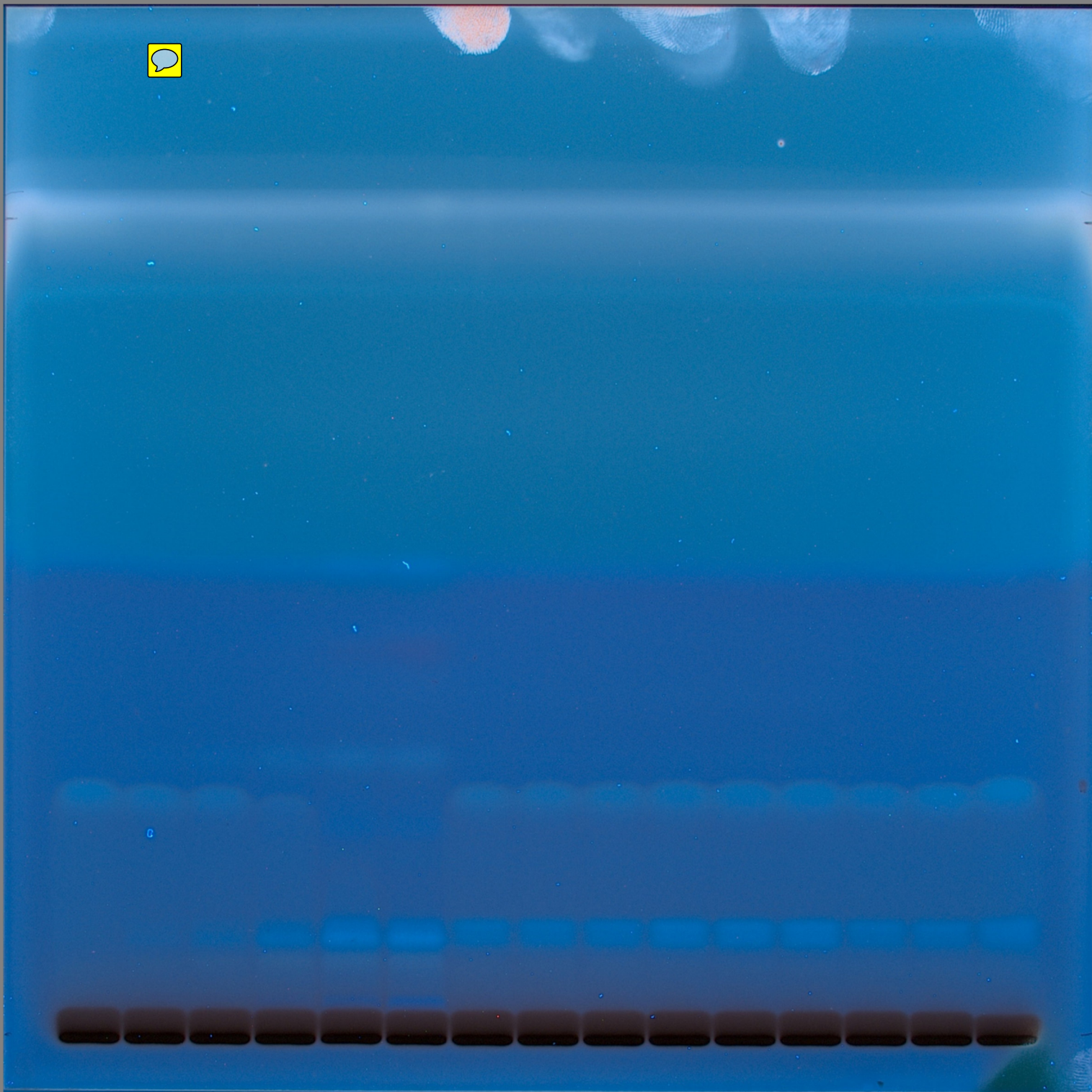

Supplement: S1 Raw images — (PDF) [file pone.0253811.s036.pdf]

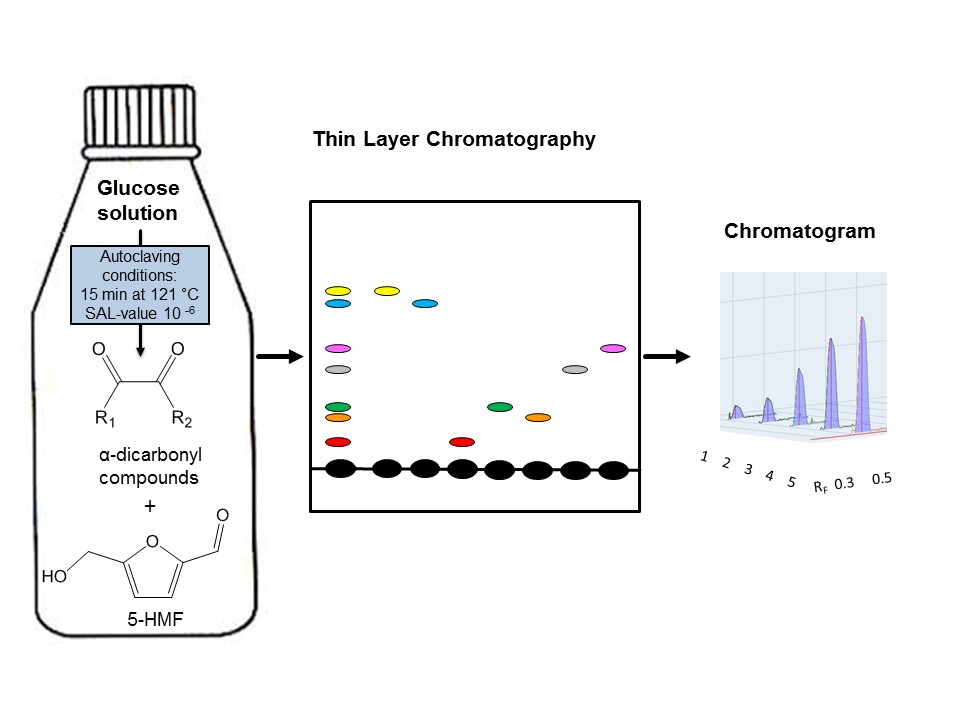

Supplement: S1 Graphical abstract — (TIF) [file pone.0253811.s037.tif]
