# Supplementary material for: Correction: Identification and quantification of glucose degradation products in heat-sterilized glucose solutions for parenteral use by thin-layer chromatography
Source: PLoS One. 2025 Apr 10;20(4):e0322667. doi: 10.1371/journal.pone.0322667 (PMC11984714; doi:10.1371/journal.pone.0322667)
Supplement: S1 File — This dataset provides an overview of empirically tested eluent compositions for the qualitative separation of derivatized GDPs (GO, MGO, 2-KDG, 3-DG, 3-DGal, 3,4-DGE, and 5-HMF) using OPD. Various solvent systems were explored to optimize chromatographic resolution, with results documented in TLC images (Figs S1-S7) and LC-MS/MS spectra (Figs S8-S15). The dataset also evaluates derivatization efficiency (Figs S16-S21) and HPTLC quantification (Figs S22-S31), detailing calibration and regression analysis. (DOCX) [file pone.0322667.s001.docx]

**Supplement**

**Eluent development**

The empirically ascertained and tested eluent compositions are listed in S1 Table. The table provides an overview of the fluid compositions used and developed to qualitatively demonstrate the GDPs GO, MGO, 2-KDG, 3-DG, 3-DGal, 3,4-DGE, and 5-HMF derivatized with OPD. All analytes were present at a concentration level of 0.5 mg/mL.

**S1 Table. Overview of tested mobile phase compositions.**

| **Eluent ( v/v or v/v/v)** | **Figure** |
| --- | --- |
| Water-methanol-glacial acetic acid-dichloroethane (10:15:25:50, v/v/v/v) | Data not shown |
| Methanol-ethyl acetate (30:70, v/v) | S1 Fig |
| Methanol-dichloromethane (30:70, v/v) | S2 Fig |
| Methanol-toluene (50:50, v/v) | S3 Fig |
| 1,4-Dioxane-toluene (95:5, v/v) | S4 Fig |
| 1,4-Dioxane-toluene-glacial acetic acid (45:45:10, v/v/v) | S5 Fig |

**All chromatograms were evaluated at 366 nm after being stained with thymol-sulfuric acid.**

First, the solvent water-methanol-glacial acetic acid-dichloroethane (10:15:25:50, v/v/v/v) was used. According to the pharmacopoeia monograph, it was originally used to separate fructose, glucose, lactose, and sucrose. In the present method it served to test its suitability for separating the derivatized GDPs from each other and from glucose [1]. As this separation was insufficient, further solvent compositions were tested.

An eluent needed be found which, although still polar, should have slightly less elution power than the one just described. The aim was to achieve lower Rf values due to the absence of the OH-bonding and polar interactions, which in the previous eluent composition were mainly caused by glacial acetic acid and water. In addition, the eluent should initially consist of only two components in order to estimate the effects of the individual eluent components more clearly.

The eluent composition methanol-ethyl acetate (30:70, v/v) was tested (S1 Fig). The Rf values were within a reasonable range (Rf between 0.35-0.7) and the colors of the spots were characteristic. Unfortunately, all derivatized GDPs, but especially glucose, smear at Rf = 0.25. GO and MGO ran equally high Rf (Rf = 0.62), but overlapped with OPD, 3.4-DGE, and 5-HMF (Rf = 0.6-0.7). 2-KDG had an Rf of 0.35, 3-DGal one of 0.42, and 3-DG one of 0.45. The next step was to try out a solvent component with lower elution force and fewer polar interactions. Therefore, ethyl acetate was replaced by dichloromethane.

Next, the eluent mixture methanol-dichloromethane (30:70, v/v) was tested (S2 Fig).

The aim of replacing ethyl acetate with dichloromethane was to make the eluent even less polar. Furthermore, the influence of hydrophobic interactions (quinoxaline-dichloromethane) needed to be investigated. The derivatized GDPs showed very high Rf values (0.5-0.95) and 5-HMF, 3,4-DGE, GO, and MGO ran almost to the solvent front (RF = 0.9-0.95). In addition, they all had an almost identical running height, which must be evaluated very critically, as the substances cannot be separated from each other in a potential mixture. 2-KDG had an Rf value of 0.5 and 3-DG had an Rf value of 0.63. Application errors occurred with 3-DGal and 3.4-DGE. The result of this eluent was not satisfactory because the Rf values were too high. Accordingly, the next eluent tested had to become less polar. In addition, the analytes needed to be better separated.

To make the eluent less polar and to reduce the running height, toluene was used instead of dichloromethane. Methanol was initially retained as a component as the derivatized GDPs nevertheless have polar substructures, and the bands of the analytes show a characteristic coloration. The newly tested eluent composition was methanol-toluene (50:50, v/v) (S3 Fig). The Rf values were in a good range (between 0.45-0.74). Unfortunately, the substances smeared, were blurred, and were too close together; presumably glucose ran up because the solvent was still too polar. 2-KDG, 3-DGal, and 3-DG were very close together and showed Rf values of 0.45, 0.52, and 0.55, respectively. GO and MGO had an Rf value of 0.68, 5-HMF and 3,4-DGE one of 0.74.

As the spots were not well separated and smeared, MeOH was replaced with 1,4-dioxane, which had a significantly weaker elution power in the next step. It was suspected that polar interactions of methanol, caused by its OH groups, hindered a good and sharp separation of the analytes. Finally, the mixing ratios (50:50, v/v), (80:20, v/v), (20:80, v/v) (data not shown) and (95:5, v/v) were tested for 1,4-dioxane-toluene. With the composition 1,4-dioxane-toluene (50:50, v/v), it was possible for the first time to clearly separate the OPD-derivatized products 2-KDG, 3-DG, and 3-DGal from each other optically, to distinguish them by absorption at 366 nm, and to retain the glucose spot at the starting line. Additionally, the derivatized analytes 2-KDG, 3-DG, and 3-DGal could be determined after staining with thymol-sulfuric acid reagent and heating by a strong fluorescent color. With the eluent composition 1,4-dioxane-toluene (95:5, v/v), sharper spots and lower Rf values were thus expected (S4 Fig). The effect of hydrophobic interactions of quinoxalines with 1,4-dioxane in relation to methanol was tested. The Rf values were significantly lower (0.15-0.65). Unfortunately, 3,4-DGE ran into the OPD spot at Rf = 0.5. 2-KDG, 3-DGal, and 3-DG achieved Rf values of 0.15, 0.25, and 0.28, respectively. The result showed that the spots were already significantly sharper. However, both spot sharpness and running height had to be optimized.

A polar modifier in the form of acetic acid was added to obtain sharper separation zones by suppressing interactions of the silica gel with the derivatized GDPs by filling the surface-active loci of the silica gel with acetic acid. The eluent composition 1,4-dioxane-toluene-glacial acetic acid (45:45:10, v/v/v) was tested (S5 Fig). Another positive aspect of adding acetic acid was the fact that the Rf values of the analytes increased slightly (0.2-0.6). 2-KDG, 3-DGal, and 3-DG achieved Rf values of 0.20, 0.22, and 0.24, respectively. MGO and GO achieved Rf values of 0.6. With 3,4-DGE an application error occurred. The derivatized 5-HMF was, for whatever reason, not visible on this TLC plate. Probably it was overlaid by the OPD spot. However, an undesirable side effect of the high acetic acid concentration was a strong light blue background signal that appeared around the OPD spot in the Rf range of 0.38-0.65.

These streaks were eliminated with the change towards the final eluent composition 1,4-dioxane-toluene-glacial acetic acid (49:49:2, v/v/v) (see manuscript, Figs 2A and 2B).

Figures S1-S5 provide an overview of the fluid compositions used and developed to separate the GDPs GO, MGO, 2-KDG, 3-DG, 3-DGal, 3,4-DGE, and 5-HMF derivatized with OPD. All analytes were present at a concentration level of 0.5 mg/mL with a volume of 10 µL at an application length of 10.0 mm.

**
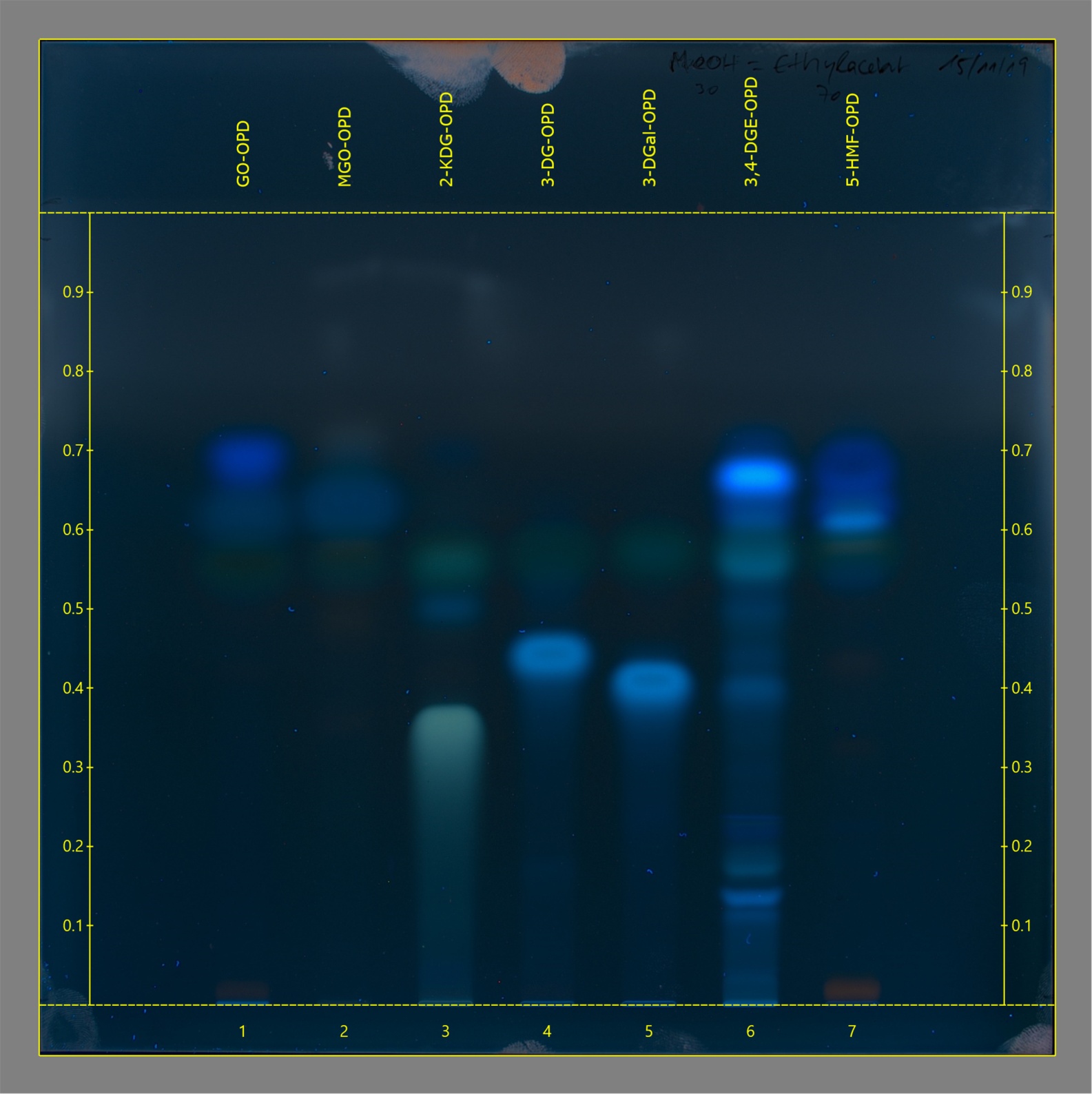
S1 Fig. TLC-plate with GDP-OPDs upon elution in methanol-ethyl acetate (30:70, v/v) and staining with thymol-sulfuric acid; image taken at the wavelength of 366 nm.**

**S2 Fig. TLC-plate with GDP-OPDs upon elution in methanol-dichloromethane (30:70, v/v) and staining with thymol-sulfuric acid; image taken at the wavelength of 366 nm.
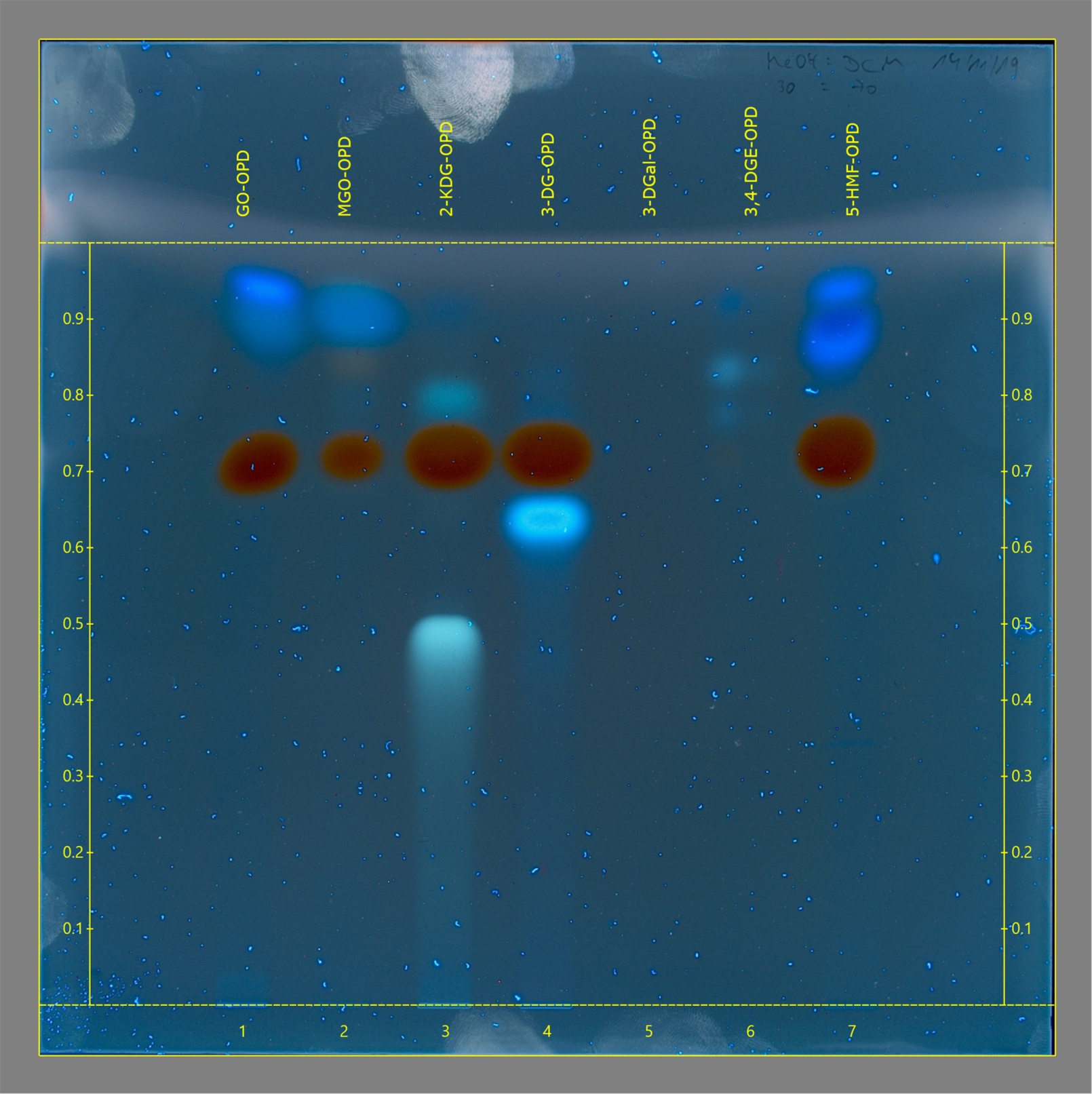
**

**S3 Fig. TLC-plate with GDP-OPDs upon elution in methanol-toluene (50:50, v/v) and staining with thymol-sulfuric acid; image taken at the wavelength of 366 nm.
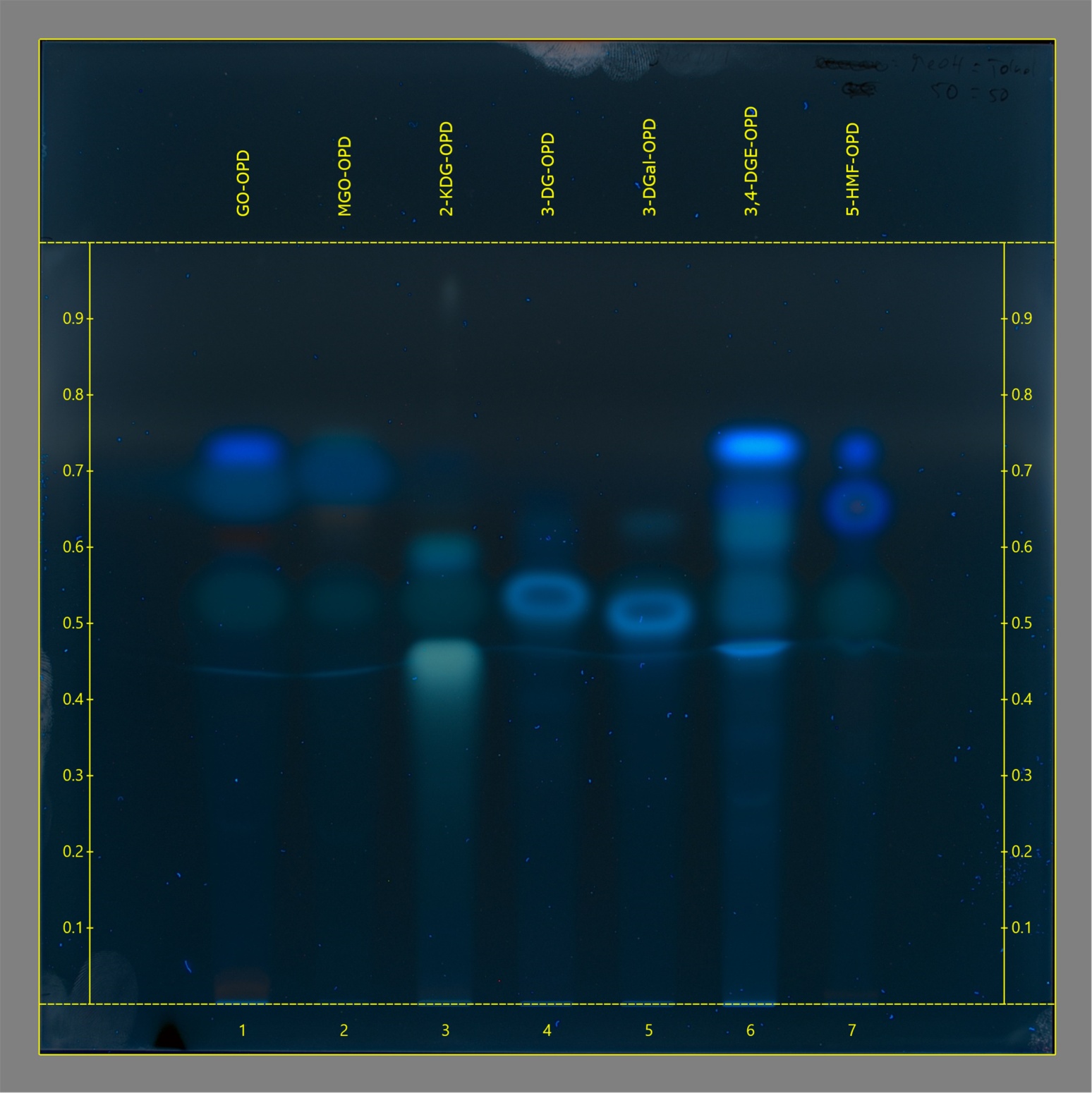
**

**S4 Fig. TLC-plate with GDP-OPDs upon elution in 1,4 dioxane-toluene (95:5, v/v) and staining with thymol-sulfuric acid; image taken at the wavelength of 366 nm.
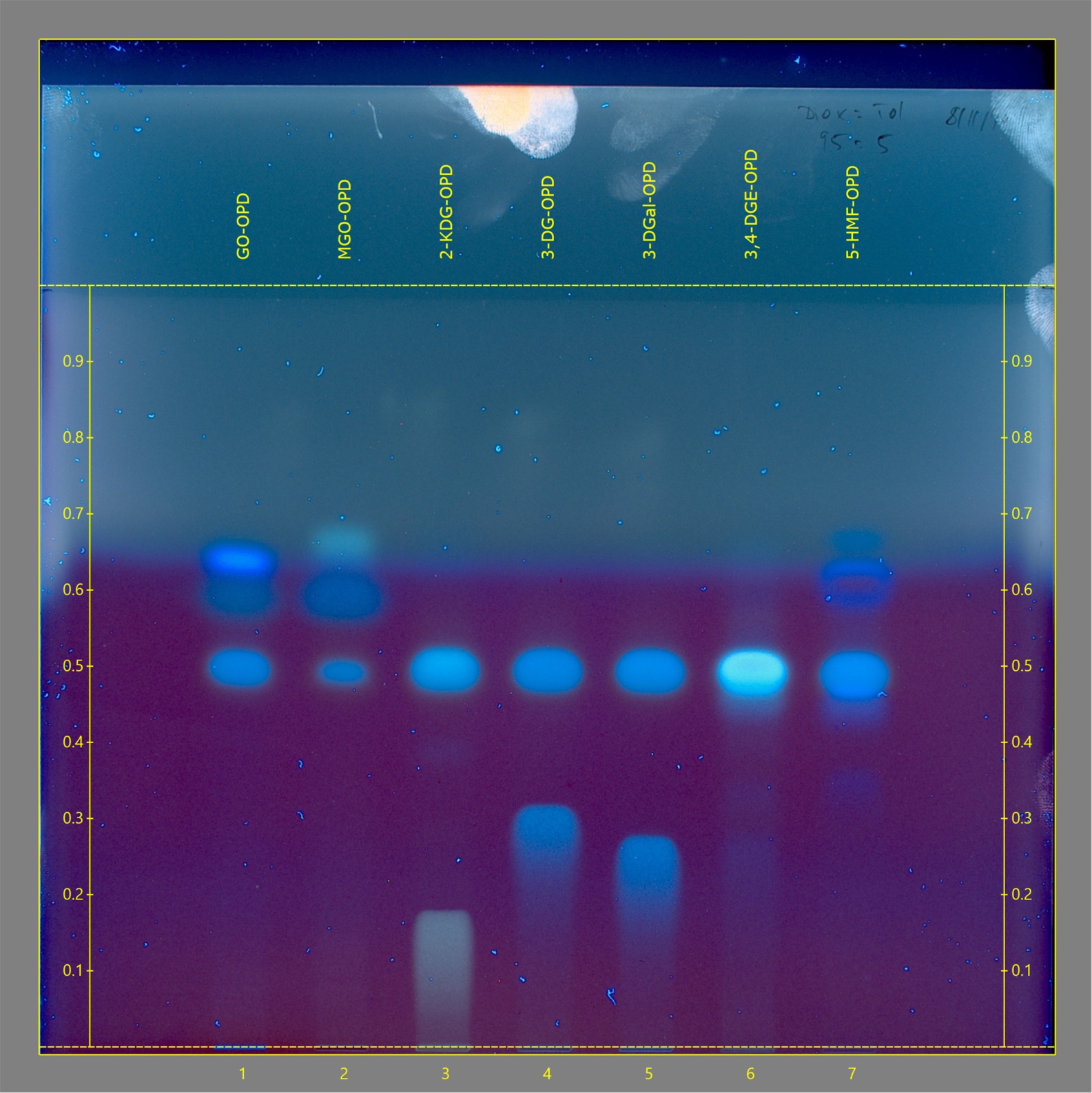
**

**
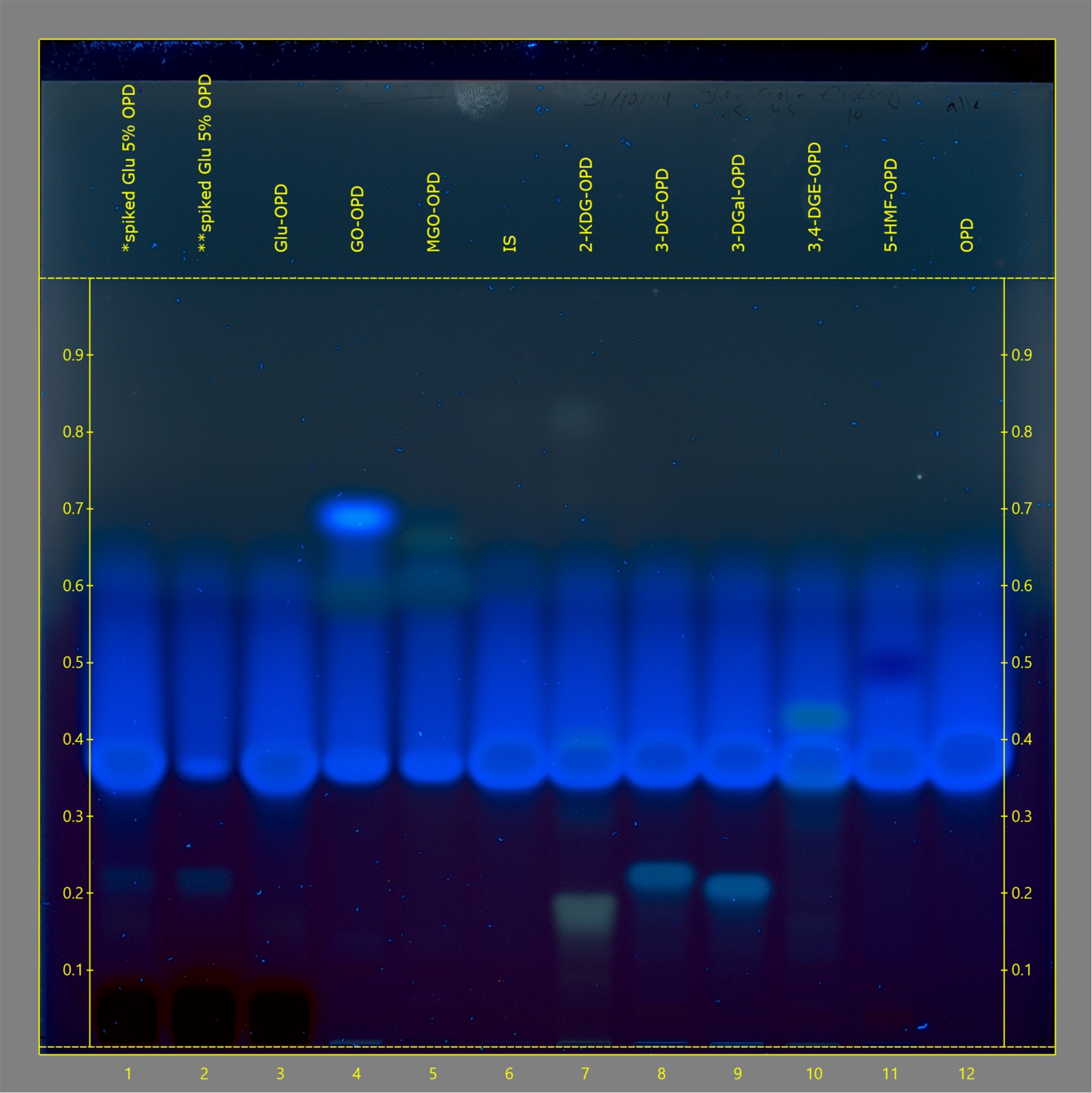
S5 Fig. TLC-plate with GDP-OPDs upon elution in 1,4 dioxane-toluene-glacial acetic acis (45:45:10, v/v/v) and staining with thymol-sulfuric acid; image taken at the wavelength of 366 nm.**

**S6 Fig. Derivatized GDP solutions with and without added glucose in order to test the influence of glucose on the consumption of the derivatization reagent OPD.**


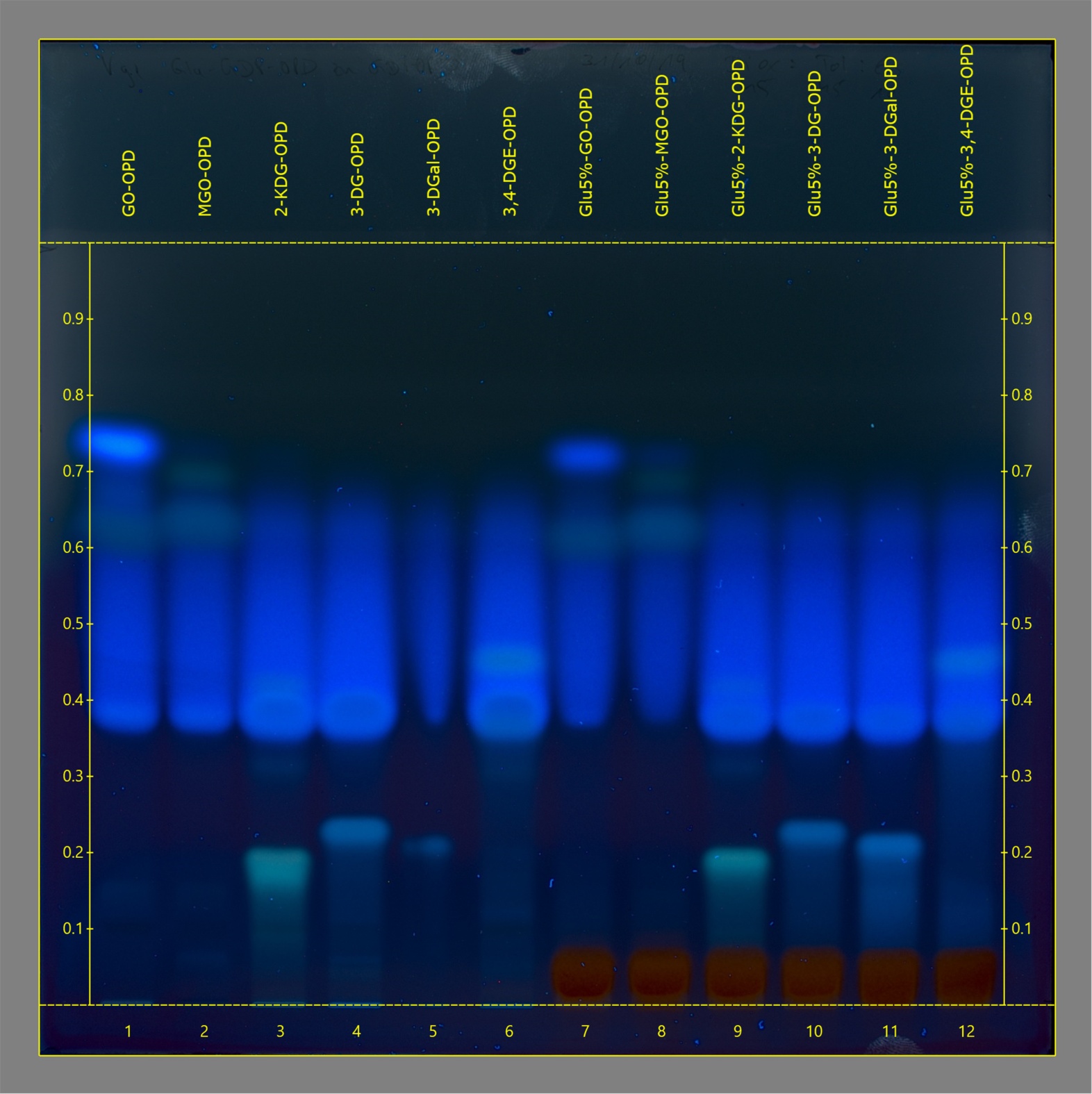
Each solution contained 1 mg/mL of the respective GDP, 3.75 mg OPD, and track 7-12 also contained 50 mg glucose. The applied volume was 10 µL at an application length of 10.0 mm. The objective was to determine the influence of glucose on OPD and its degradation products.

**Qualitative HPTLC analysis of the derivatized GDPs with the final eluent after treatment with thymol-sulphuric acid on a 10 x 20 cm plate (landscape orientation)**

The derivatized GDPs were prepared as described in the manuscript in section 2.2.1 and qualitatively developed on the HPTLC plate as described in section 2.2.3. However, 10 x 20 cm plates were used instead of the 20 x 20 cm plates used in the manuscript and the migration distance was 7 cm instead of the 16 cm reported in the manuscript. The rationale for the experiment was the question of whether the bands could be displayed even narrower and sharper this way.

**S7 Fig. Derivatized GDP solutions and mix with added glucose in order to test the influence of the smaller migration distance (7cm).**

**
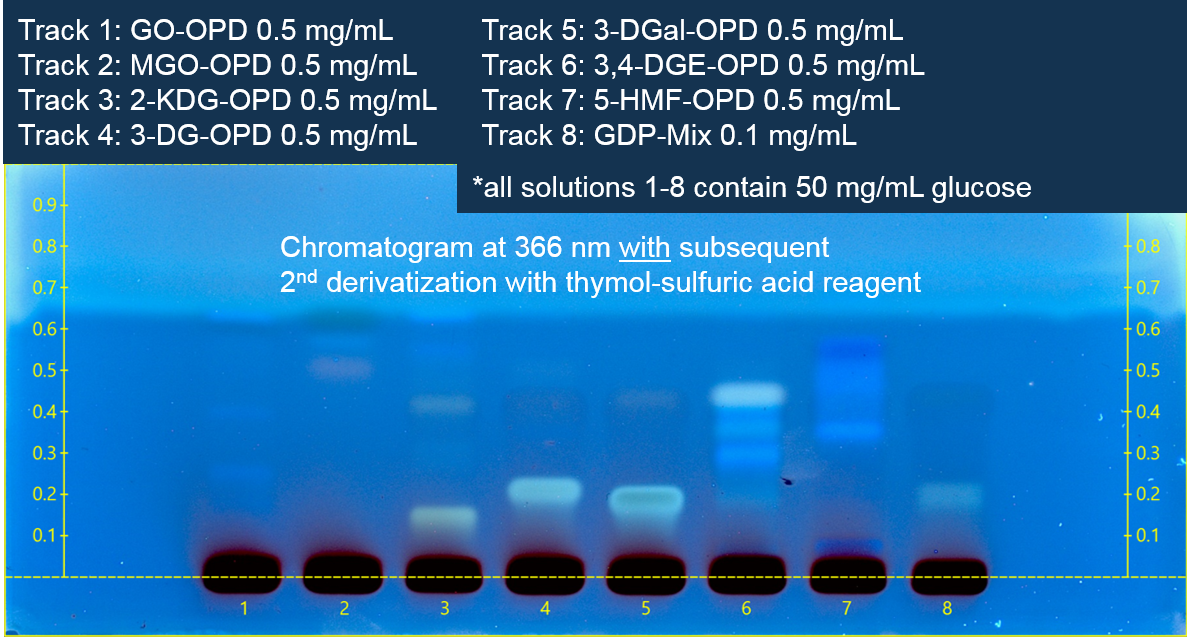
**

It could be shown that the use of the smaller plate with the shorter migration distance was detrimental for the method. The bands of the derivatized GDPs 2-KDG, 3-DGal, and 3-DG now had an almost identical Rf value, so that only qualitative and quantitative sum determinations of the three GDPs could be carried out, which represents a clear deterioration in comparison to the method described in 2.2.3. In addition, the glucose adhering to the starting line tends to excessive tailing and thus overlaps all of the three analytes mentioned. This is out of proportion to the benefit of minimally narrower bands. For this reason, the experiment was not continued quantitatively here.

**Structure elucidation and qualification of derivatized GDPs**

The following parameters were used for the structural elucidation and qualification of derivatized GDPs:

**S2 Table. LC-MS/MS parameters of all investigated GDPs derivatized with OPD.**

| **Analyte** | **Q1**  **[m/z]** | **Q3**  **[m/z]** | **Dwell time**  **[msec]** | **CE**  **[eV]** | **DP**  **[eV]** |
| --- | --- | --- | --- | --- | --- |
| 2-KDG-OPD | 251.1 | 173.2 | 50 | 20 | 100 |
|  | 251.1 | 145.1 | 50 | 20 | 100 |
| 3-DG-OPD/  3-DGal-OPD | 235.1 | 199.1 | 50 | 20 | 100 |
|  | 235.1 | 217.1 | 50 | 20 | 100 |
| 5-HMF-OPD | 215.1 | 197.1 | 50 | 25 | 100 |
|  | 215.1 | 169.1 | 50 | 25 | 100 |
| GO-OPD | 131.1 | 104 | 50 | 40 | 100 |
|  | 131.1 | 76.7 | 50 | 40 | 100 |
| 3,4-DGE-OPD | 217.1 | 169.1 | 50 | 20 | 100 |
|  | 217.1 | 181.1 | 50 | 20 | 100 |
| MGO-OPD | 145.1 | 118.1 | 50 | 40 | 100 |
|  | 145.1 | 77 | 50 | 40 | 100 |

The following figures S8-S15 show the structure elucidation by LC-MS/MS of the derivatized degradation products GO, MGO, 2-KDG, 3-DG, 3-DGal, 3,4-DGE, and 5-HMF. Shown are the Q1 [m/z] masses (the right signal in the diagram) and further structures resulting, for example, from cleavage and rearrangement. The two signals with the highest intensities were selected as Q3 [m/z] values.

**S8 Fig. Product ion mass spectrum of 2-KDG-OPD with hypothetical structure elucidation, *m/z* = 251.1, CE = 20 eV by LC-MS/MS
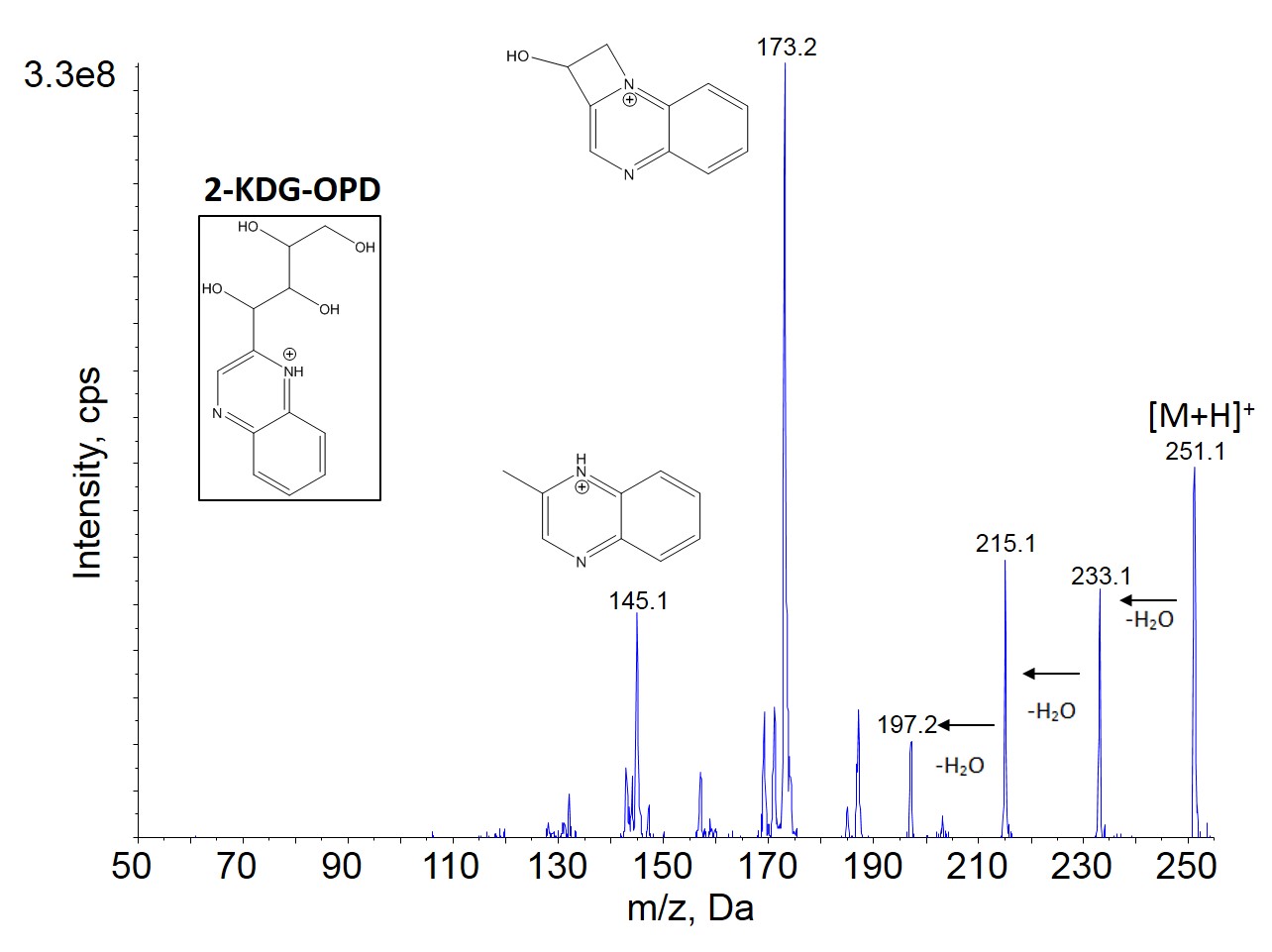
.**

**S9 Fig. Product ion mass spectrum of 3-DG-OPD with hypothetical structure elucidation, *m/z* = 235.1, CE = 20 eV by LC-MS/MS
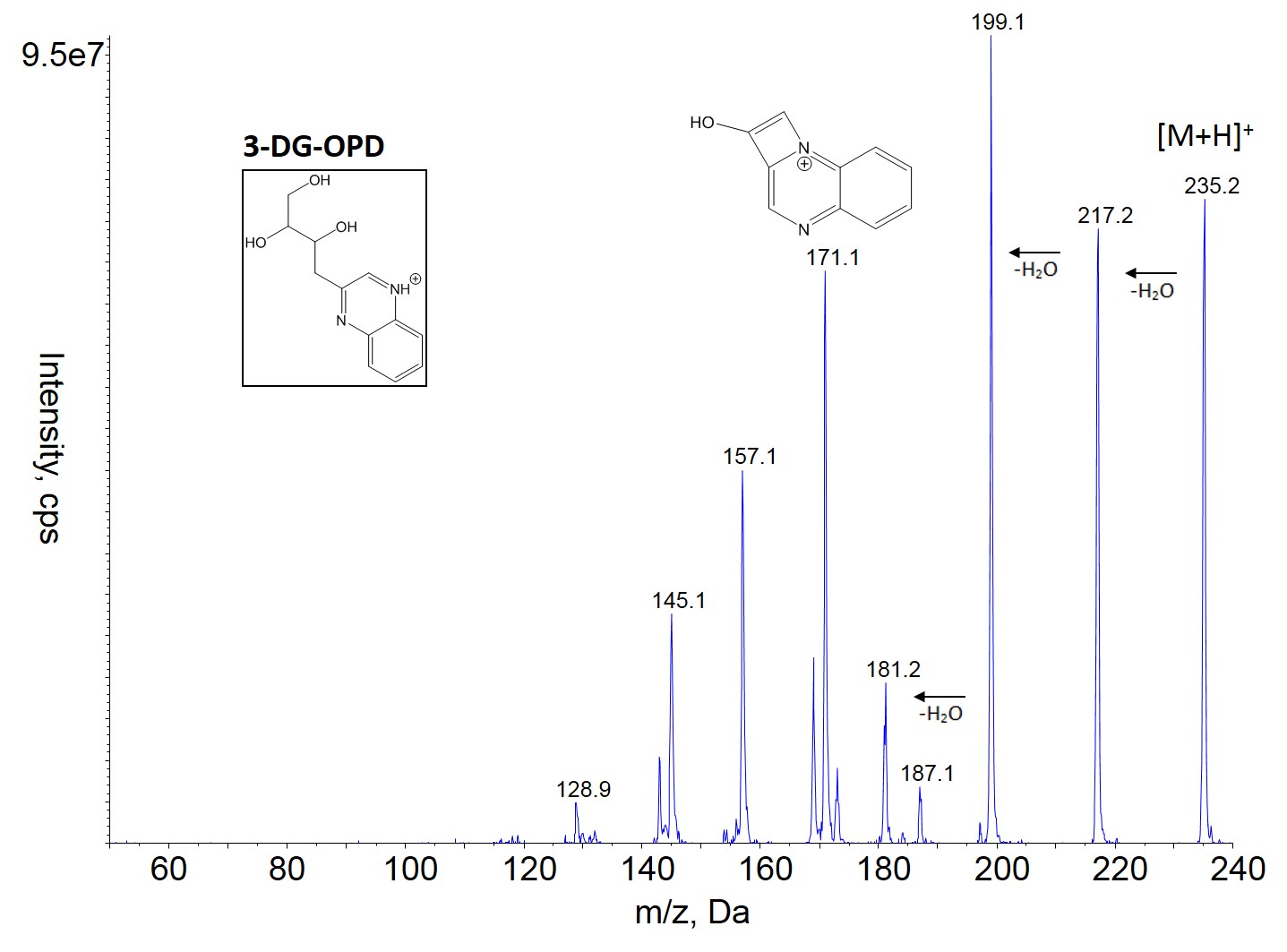
.**

**S10 Fig. Product ion mass spectrum of 3-DGal-OPD with hypothetical structure elucidation, *m/z* = 235.1, CE = 20 eV by LC-MS/MS
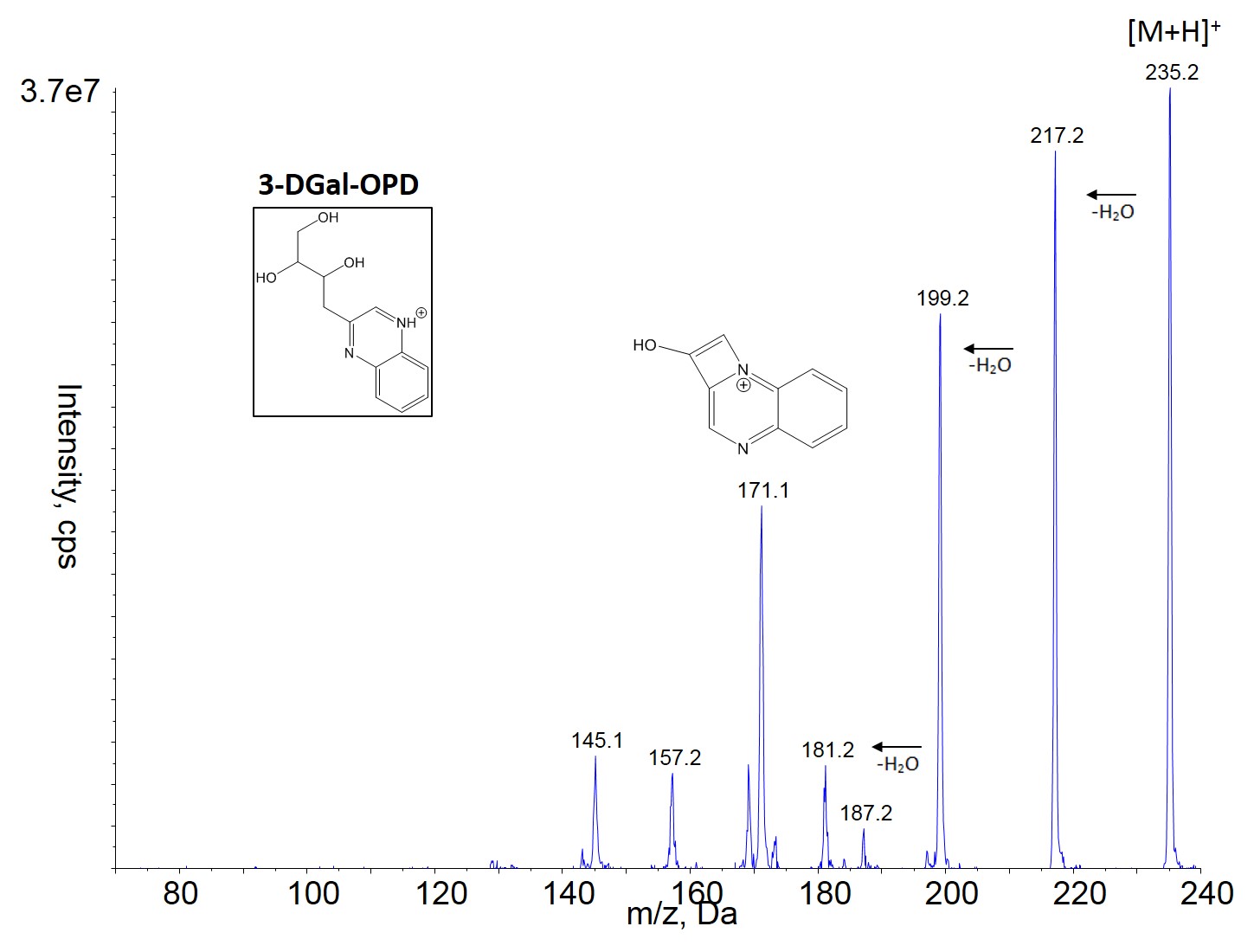
.**

**S11 Fig. Product ion mass spectrum of 5-HMF-OPD with hypothetical structure elucidation, *m/z* = 215.1, CE = 25 eV by LC-MS/MS**
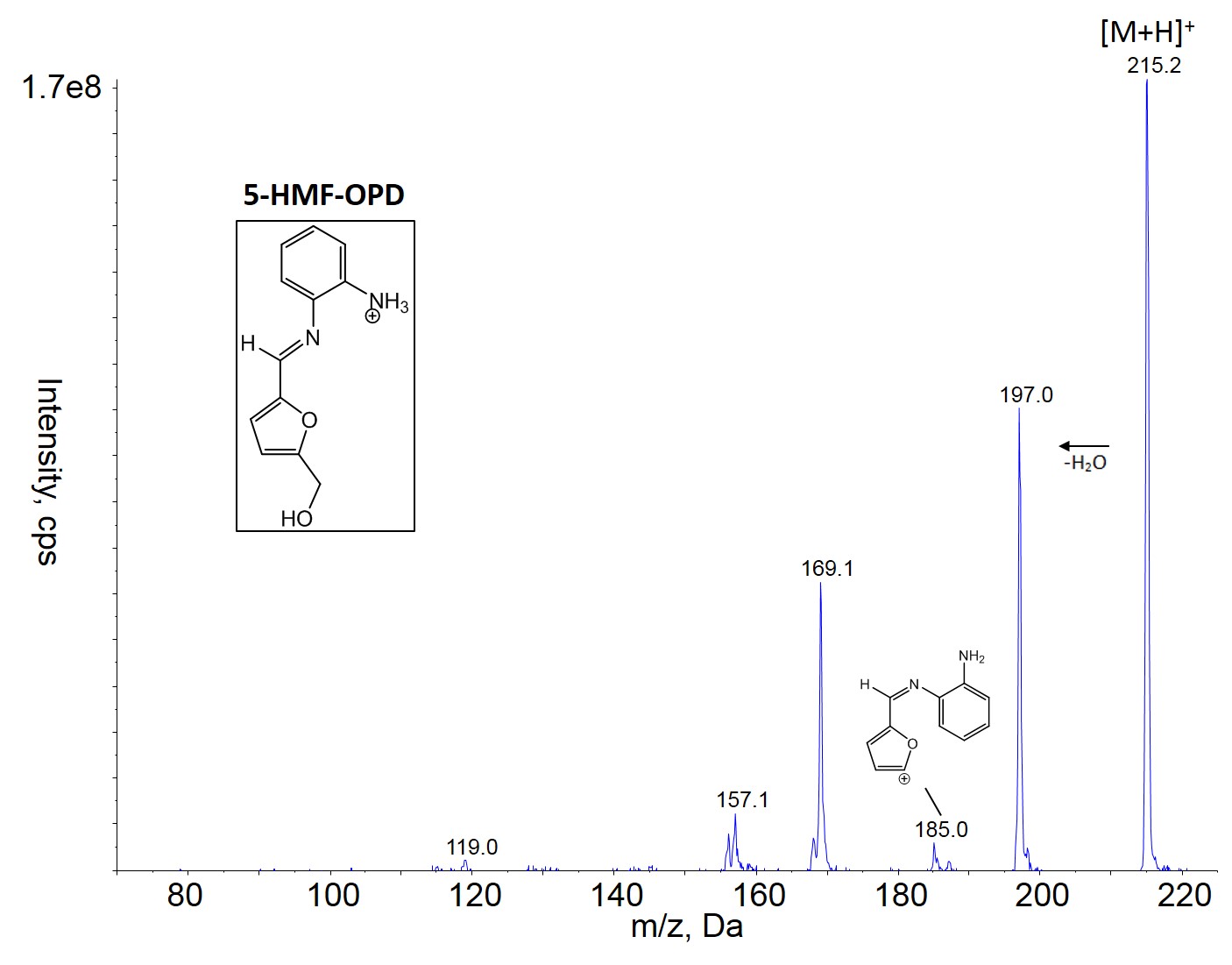
.

**S12 Fig. Product ion mass spectrum of GO-OPD with hypothetical structure elucidation, *m/z* = 131.1, CE = 40 eV by LC-MS/MS
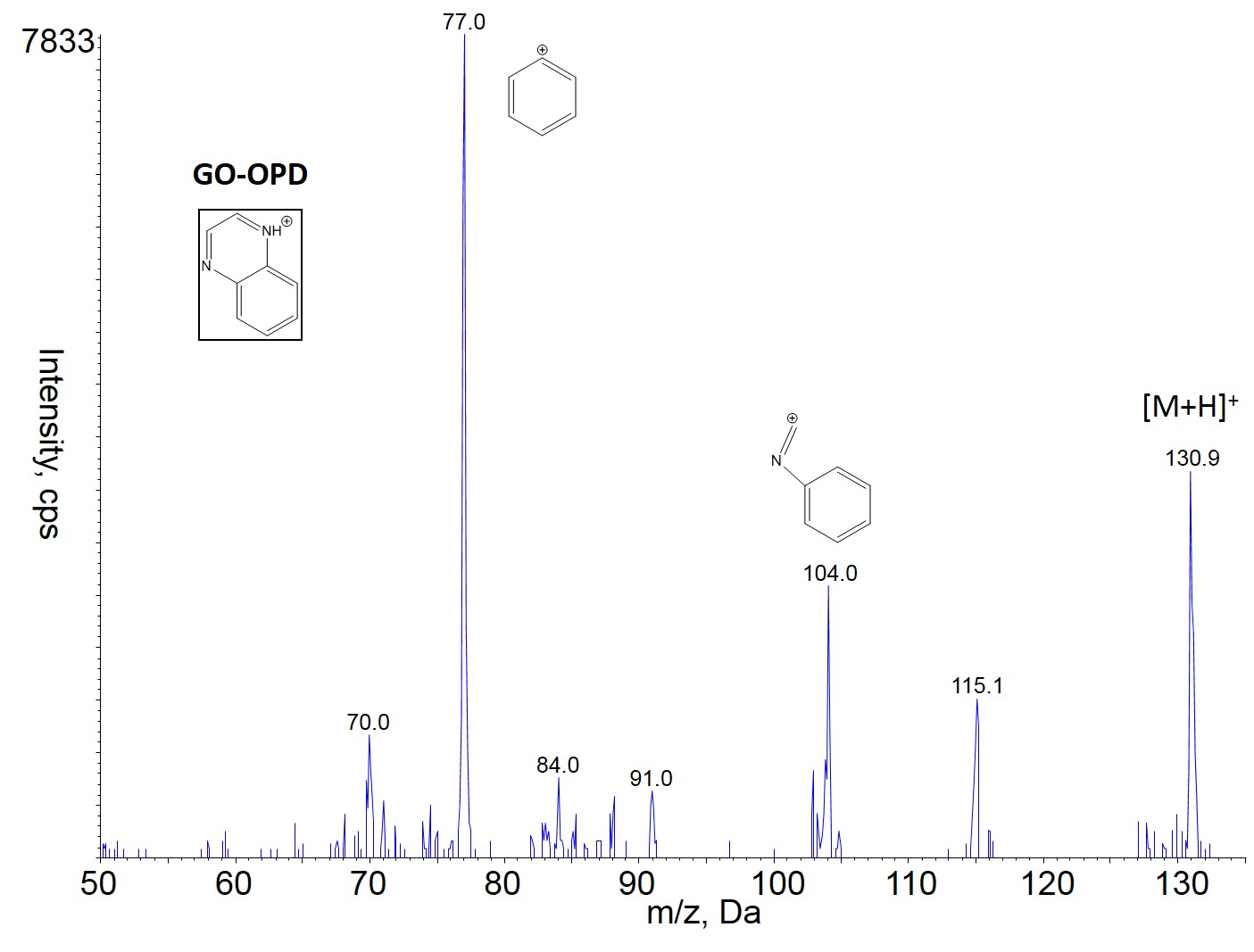
.**


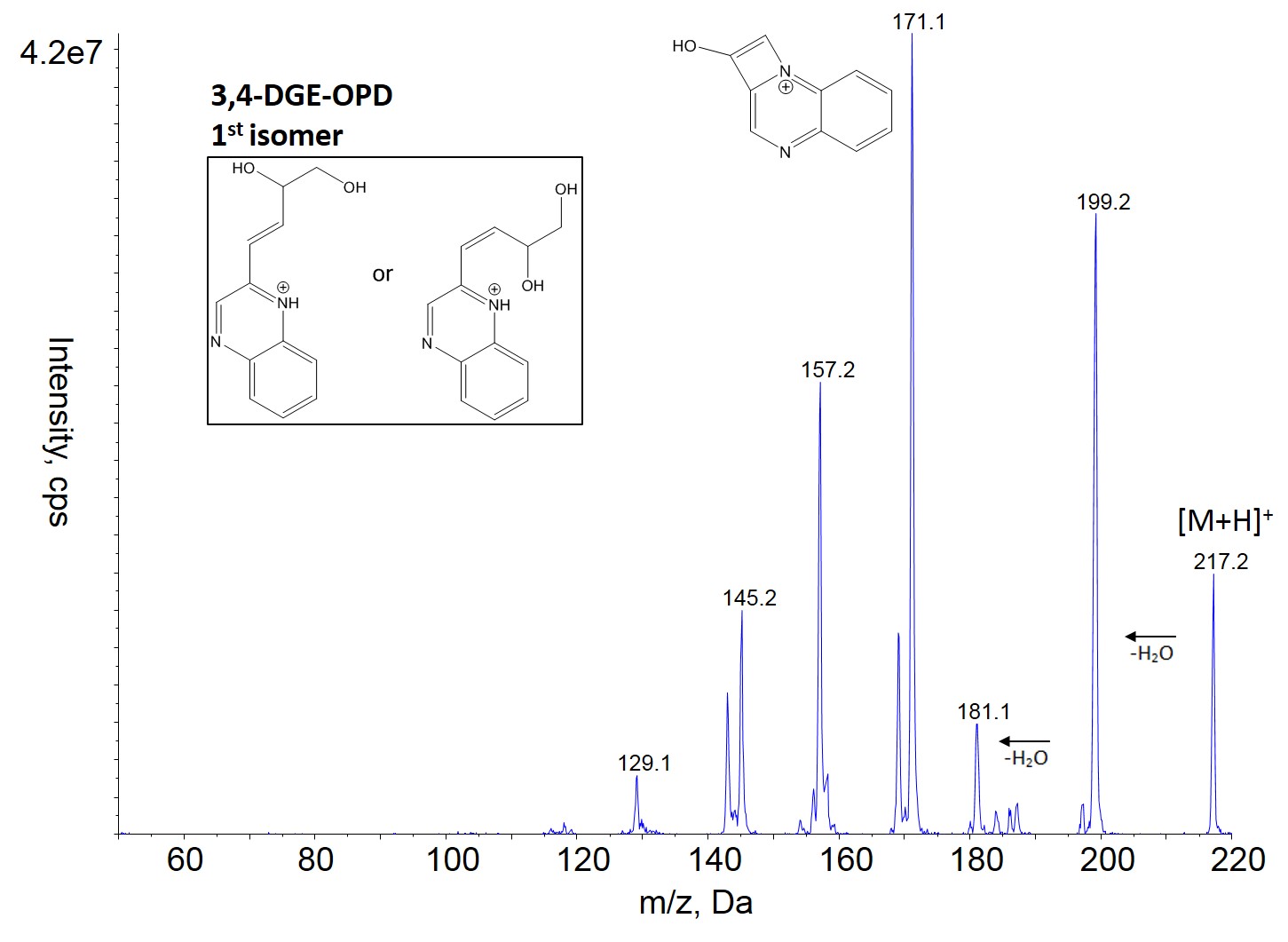
 **S13 Fig. Product ion mass spectrum of 3,4-DGE-OPD 1^st^ isomer with hypothetical structure elucidation, *m/z* = 217.1, CE = 20 eV by LC-MS/MS.**


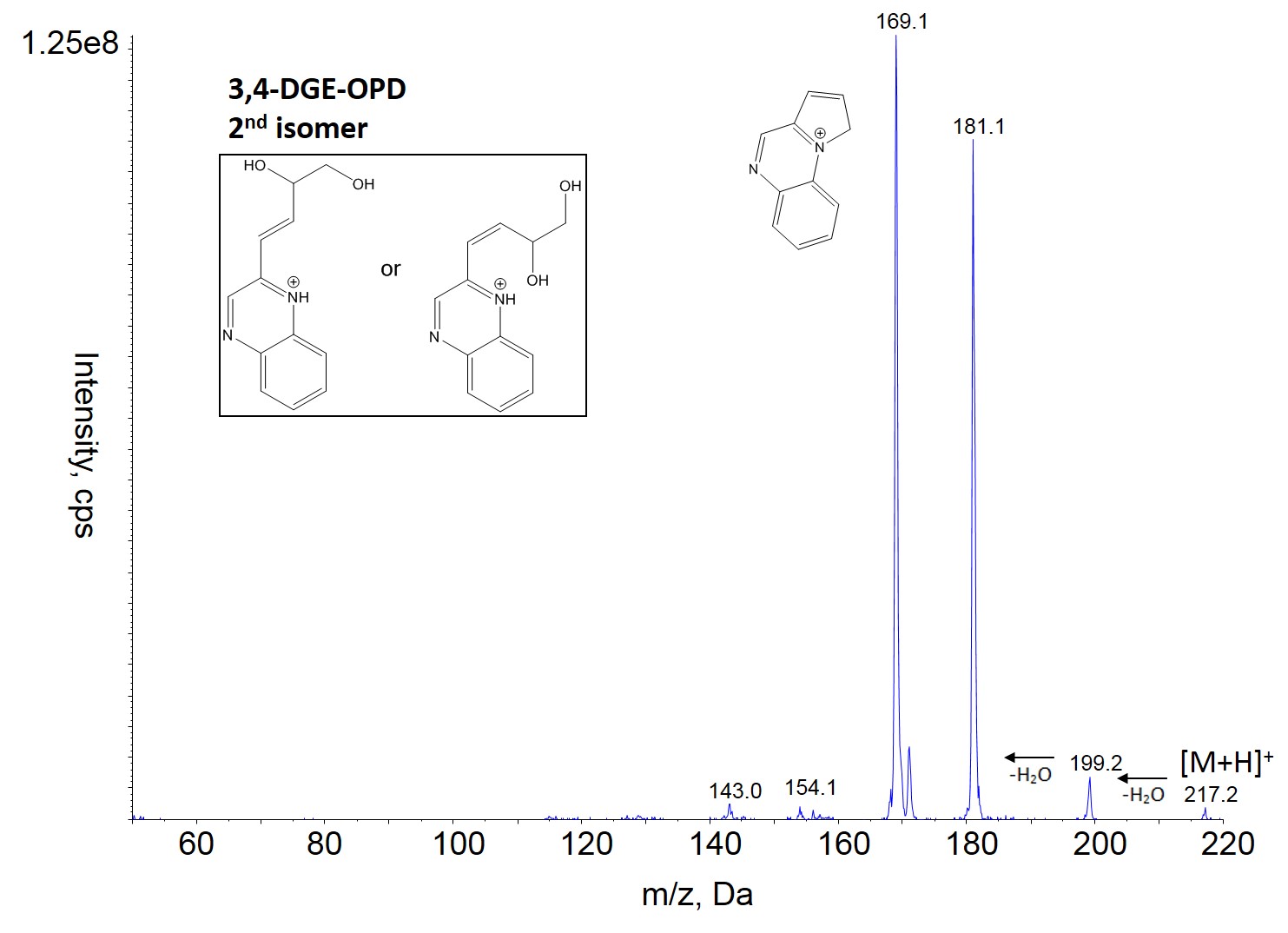
 **S14 Fig. Product ion mass spectrum of 3,4-DGE-OPD 2^nd^ isomer with hypothetical structure elucidation, *m/z* = 217.1, CE = 20 eV by LC-MS/MS.**

**S15 Fig. Product ion mass spectrum of MGO-OPD with hypothetical structure elucidation, *m/z* = 145.1, CE = 40 eV by LC-MS/MS
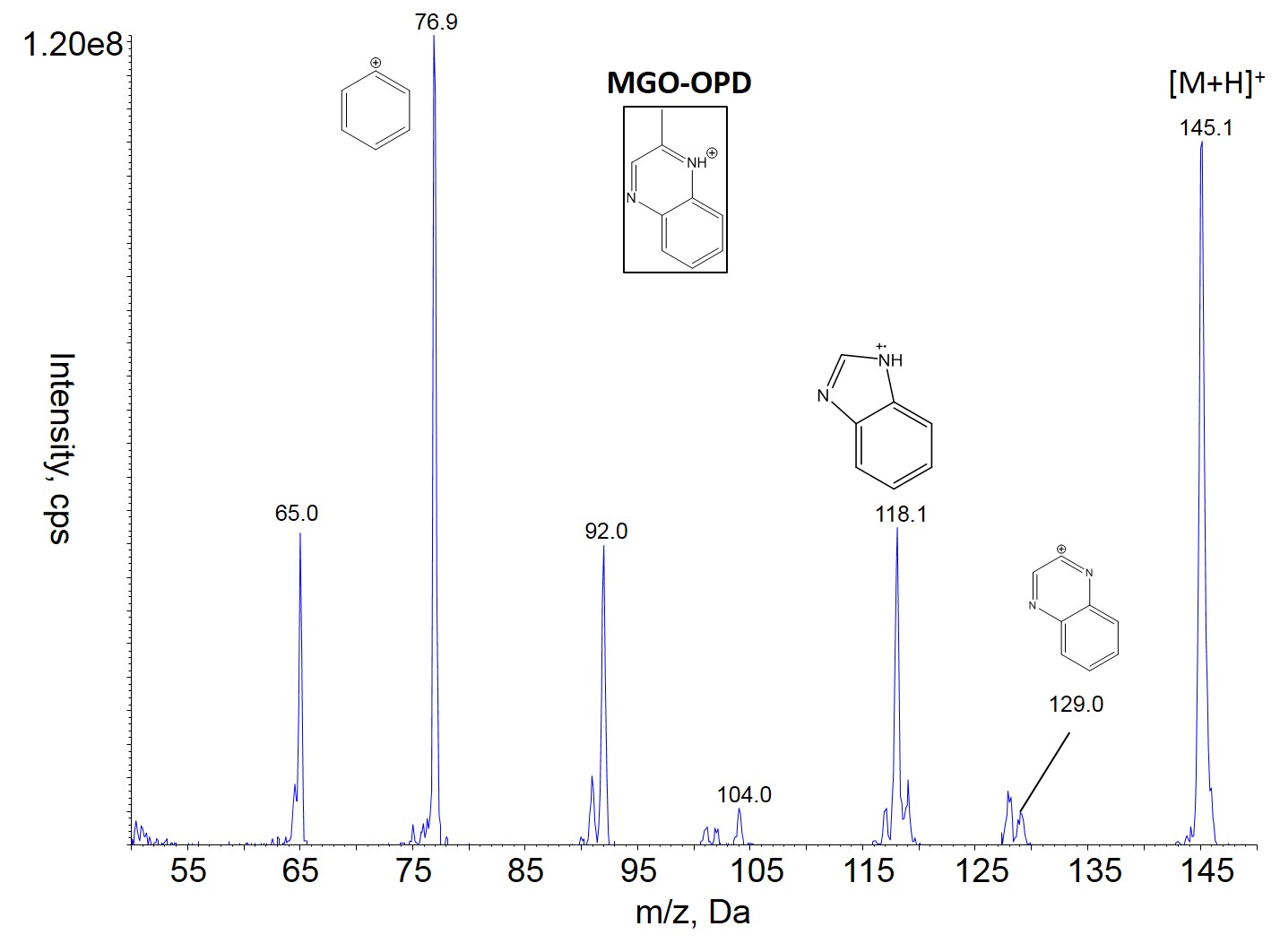
.**

**Evaluation of the derivatization procedure**

Depicted is the concentration of OPD 0.75 mg/mL +/- 20 % (0.60 mg/mL and 0.90 mg/mL) chosen for each GDP within the simultaneous quantification as well as the concentration of OPD 0.75 mg/mL without glucose matrix used in the method. The aim of the comparative measurement of the solution without glucose matrix was to determine whether the 7 GDPs were completely derivatized in the presence of the glucose.

In addition, the measurement was carried out over 24 h in order to better assess time effects.

**S16 Fig. Influence of the derivatization time and the concentration of the derivatization reagent for GO at a concentration level of 10 µg/mL.**

**S17 Fig. Influence of the derivatization time and the concentration of the derivatization reagent for MGO at a concentration level of 10 µg/mL.**

**S18 Fig. Influence of the derivatization time and the concentration of the derivatization reagent for 2-KDG at a concentration level of 10 µg/mL.**

**S19 Fig. Influence of the derivatization time and the concentration of the derivatization reagent for 3-DG/3-DGal at a concentration level of 10 µg/mL.**

**S20 Fig. Influence of the derivatization time and the concentration of the derivatization reagent for 3,4-DGE at a concentration level of 10 µg/mL.**

**S21 Fig. Influence of the derivatization time and the concentration of the derivatization reagent for 5-HMF at a concentration level of 10 µg/mL.**

The evaluation can be found in the revised manuscript in the section *Evaluation of the derivatization procedure*, pp. 15-16, lines 396-420.

**Quantification of derivatized GDPs via HPTLC**

The following figures S22-S26 show the quantification of the derivatized degradation products GO, MGO, 2-KDG, 3-DG, 3-DGal, 3,4-DGE, and 5-HMF. The integrated areas of the respective substances are shown at the specified Rf value (y-axis) in ascending concentration (x-axis).

**S22 Fig. Chromatogram of 2-KDG in the concentration range of 1-50 µg/mL at 420 nm recorded in absorption mode at Rf = 0.102
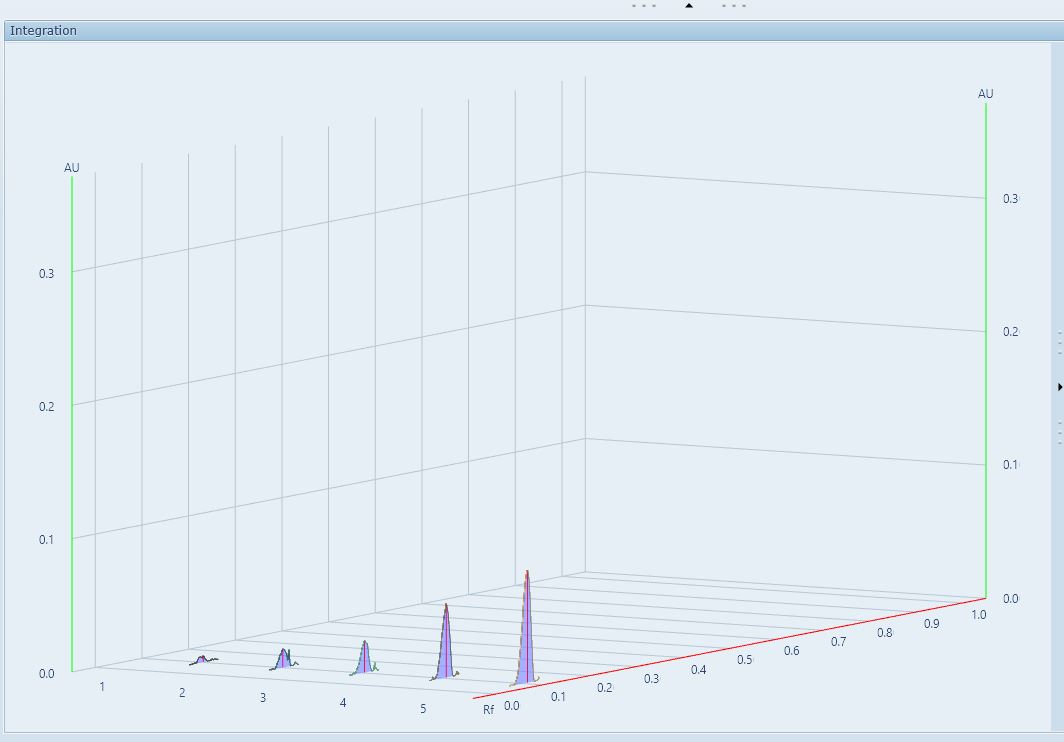
.**

**S23 Fig. Chromatogram of 3-DG/3-DGal in the concentration range of 10-150 µg/mL at 370 nm recorded in absorption mode at Rf = 0.134.**


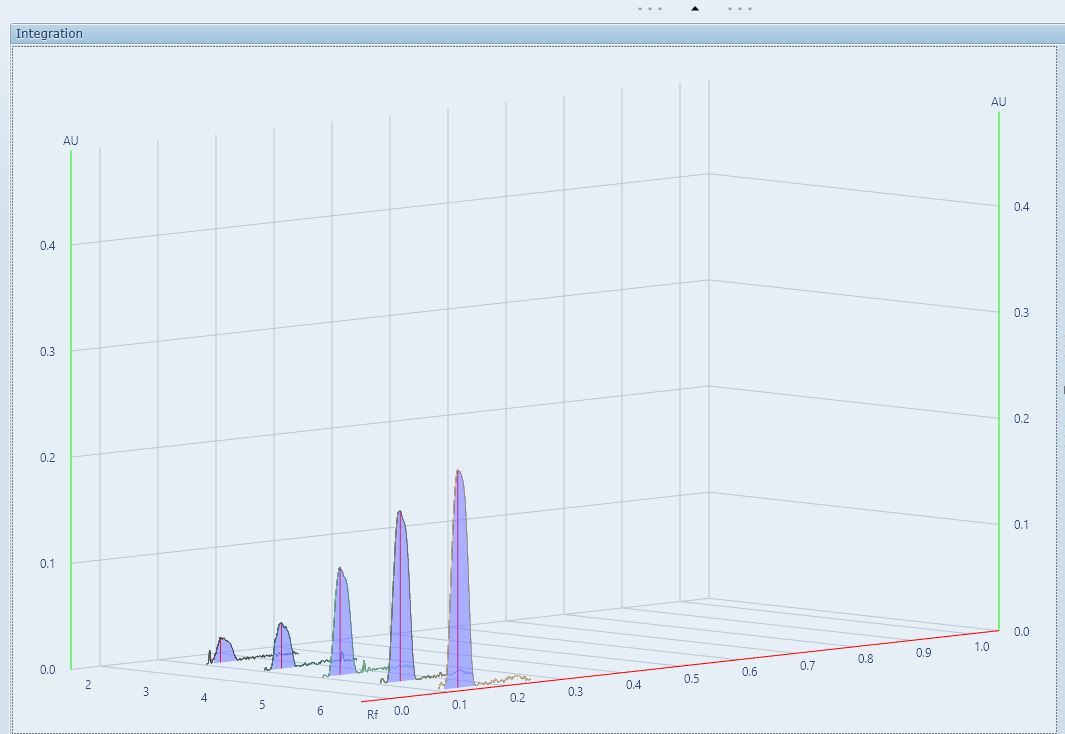


**S24 Fig. Chromatogram of 3,4-DGE in the concentration range of 1-75 µg/mL at 366 nm recorded in fluorescence mode at Rf = 0.237
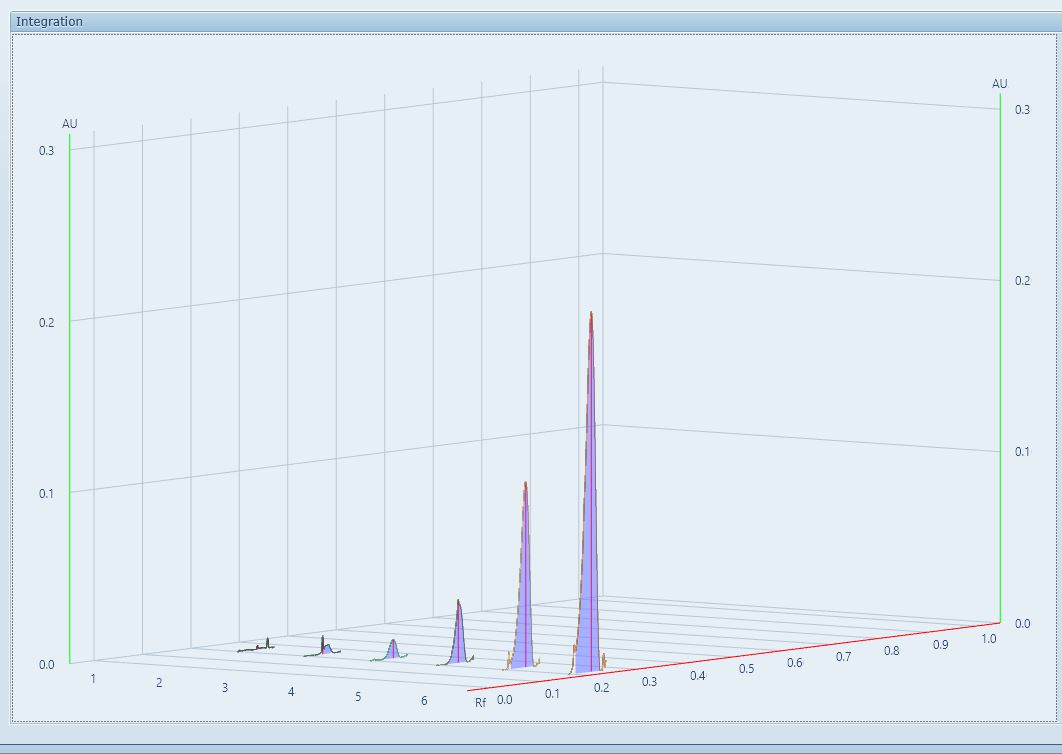
.**

**S25 Fig. Chromatogram of 5-HMF in the concentration range of 1-75 µg/mL at 330 nm recorded nm in absorption mode at Rf = 0.278
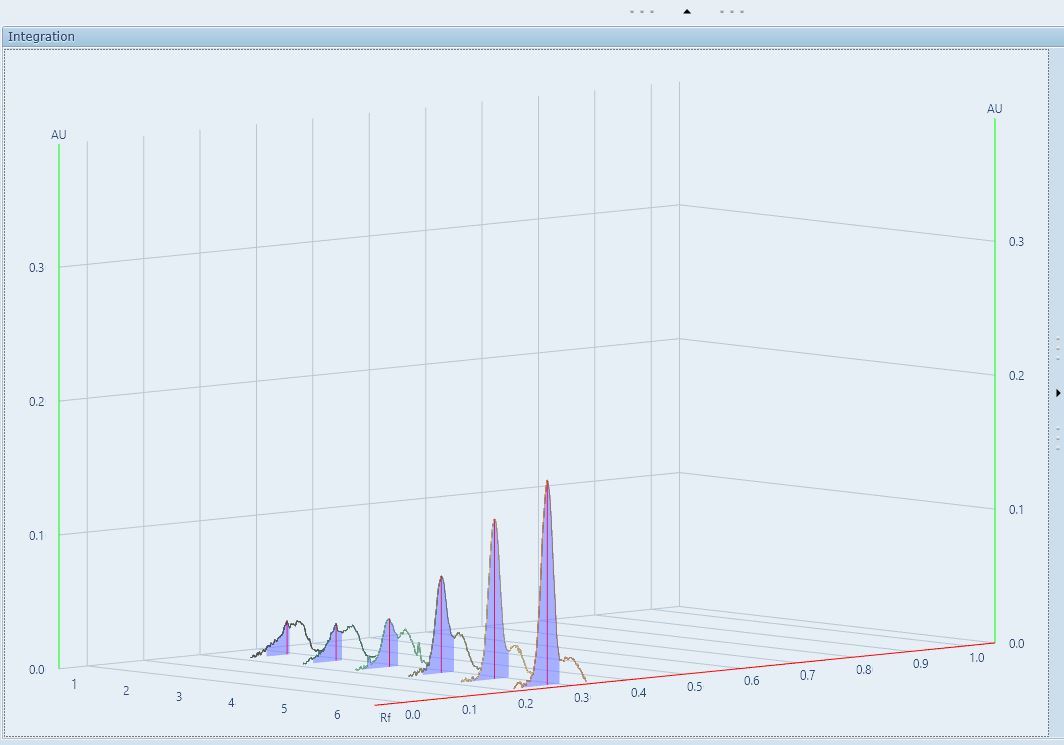
.**

**S26 Fig. Chromatogram GO/MGO in the concentration range of 2-150 µg/mL at 330 nm recorded in absorption mode at Rf = 0.571
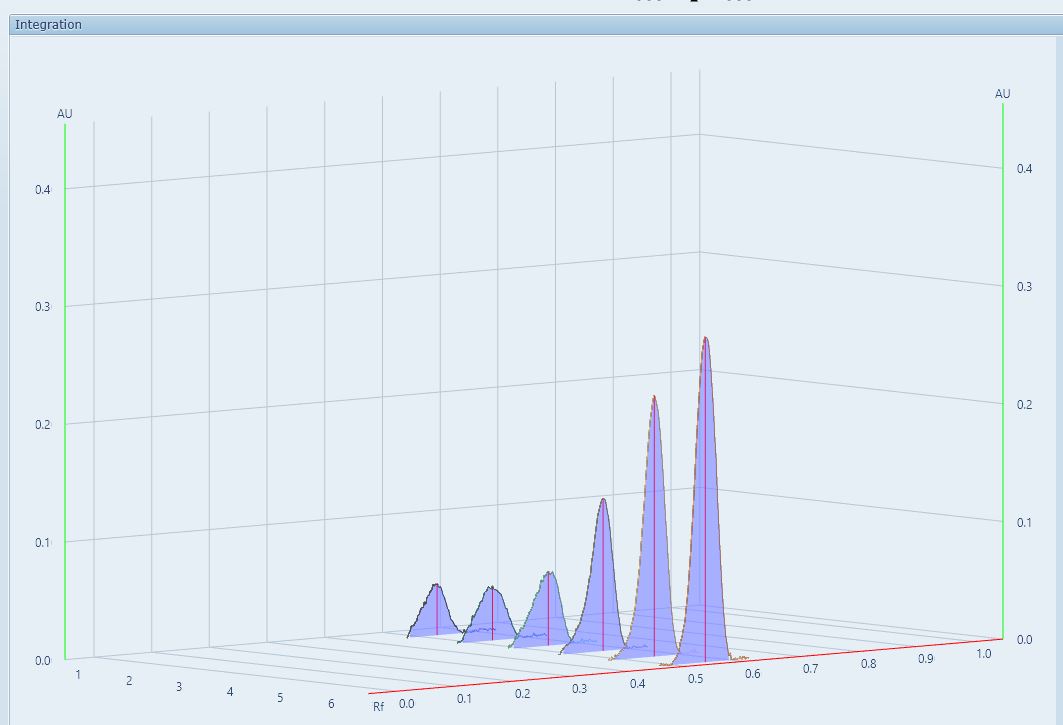
.**

**Creating the calibration function with Microsoft Excel 2016**

All chromatograms from figures S22-S26 were evaluated in Excel either by the peak area or by the peak height. Regression calculations were performed. This is shown in the following figures S27-S31.

**S27 Fig.
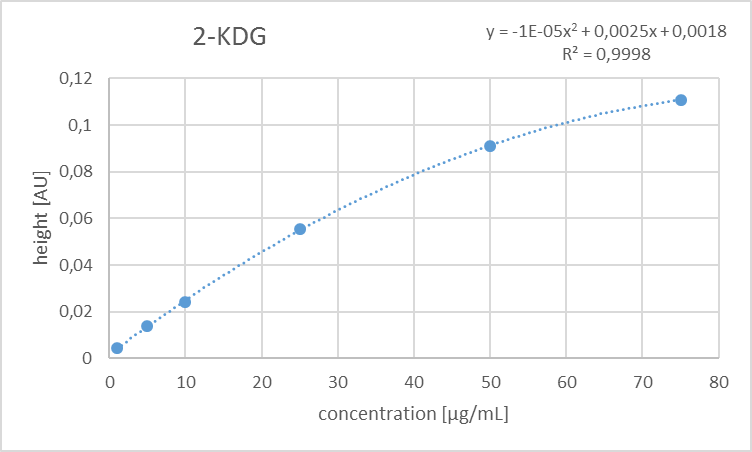
Polynomial regression of 2-KDG at a concentration level of 1-50 µg/mL at 420 nm in absorption mode at Rf = 0.102.**

**S28 Fig. Polynomial regression of 3-DG/3-DGal at a concentration level of 10-150 µg/mL at 370 nm in absorption mode at Rf = 0.134.**

**
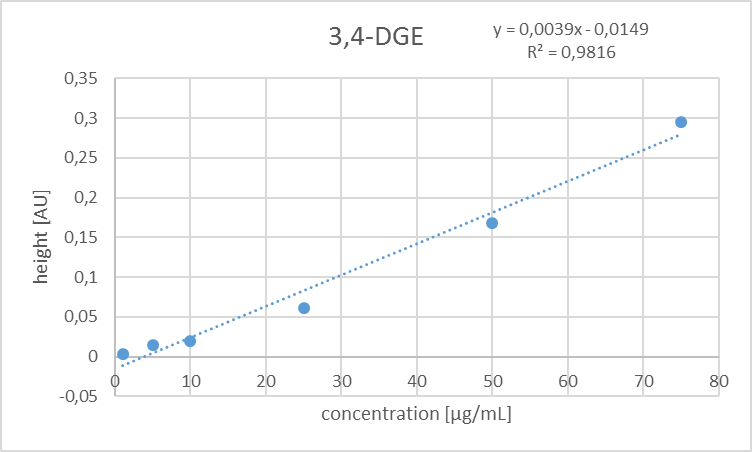
S29 Fig. Linear regression of 3,4-DGE at a concentration level of 1-75 µg/mL at 366 nm in fluorescence mode at Rf = 0.237.**

**S30 Fig. Polynomial regression of 5-HMF at a concentration level of 1-75 µg/mL at 330 nm in absorption mode at Rf = 0.278.**


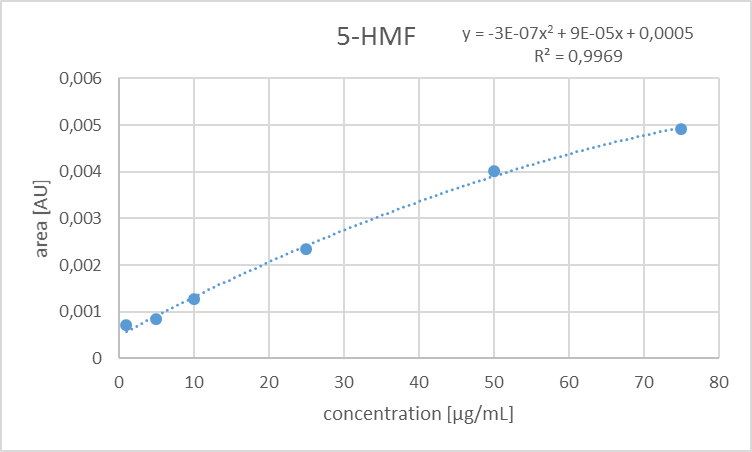


**S31 Fig. Polynomial regression of GO/MGO at a concentration level of 2-150 µg/mL at 330 nm in absorption mode at Rf = 0.571.**

**Determination of GDP concentrations in a 5 % finished drug solution. The chromatograms were shown at 366 nm without (S32 Fig) and with (S33 Fig) subsequent 2^nd^ derivatization with thymol-sulfuric acid reagent.**

The concentrations of the 7 degradation products GO/MGO, 2-KDG, 3-DG/3-DGal, 3,4-DGE, and 5-HMF in a 5 % glucose solution in a polypropylene bottle were investigated (see manuscript section 2.2.7). The HPTLC plate with the corresponding calibration solutions as well as the test solutions (triplicate measurement) is shown here.

**S32 Fig. Analysis of a 5% finished drug solution at 366 nm without subsequent 2^nd^ derivatization with thymol-sulfuric acid reagent.**
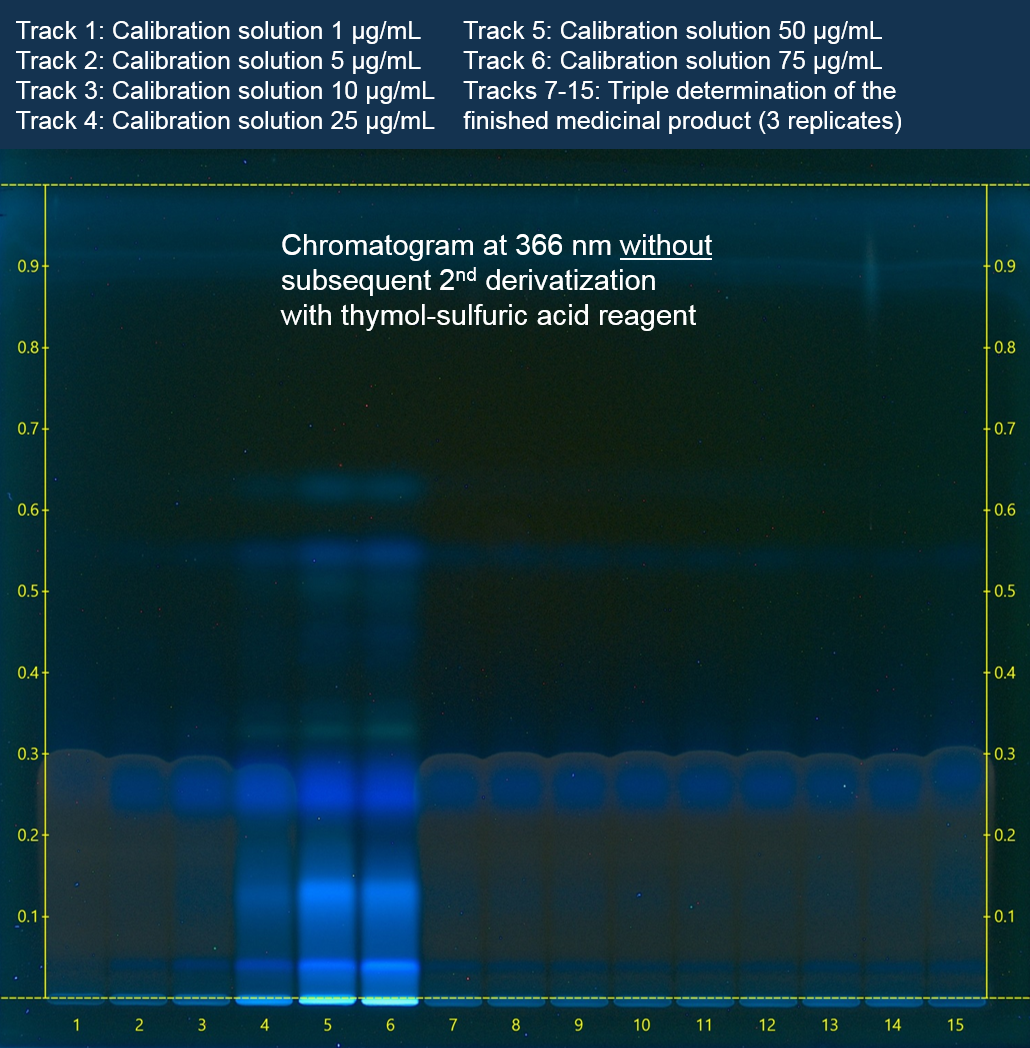


**S33 Fig. Analysis of a 5% finished drug solution at 366 nm with subsequent 2^nd^ derivatization with thymol-sulfuric acid reagent.**
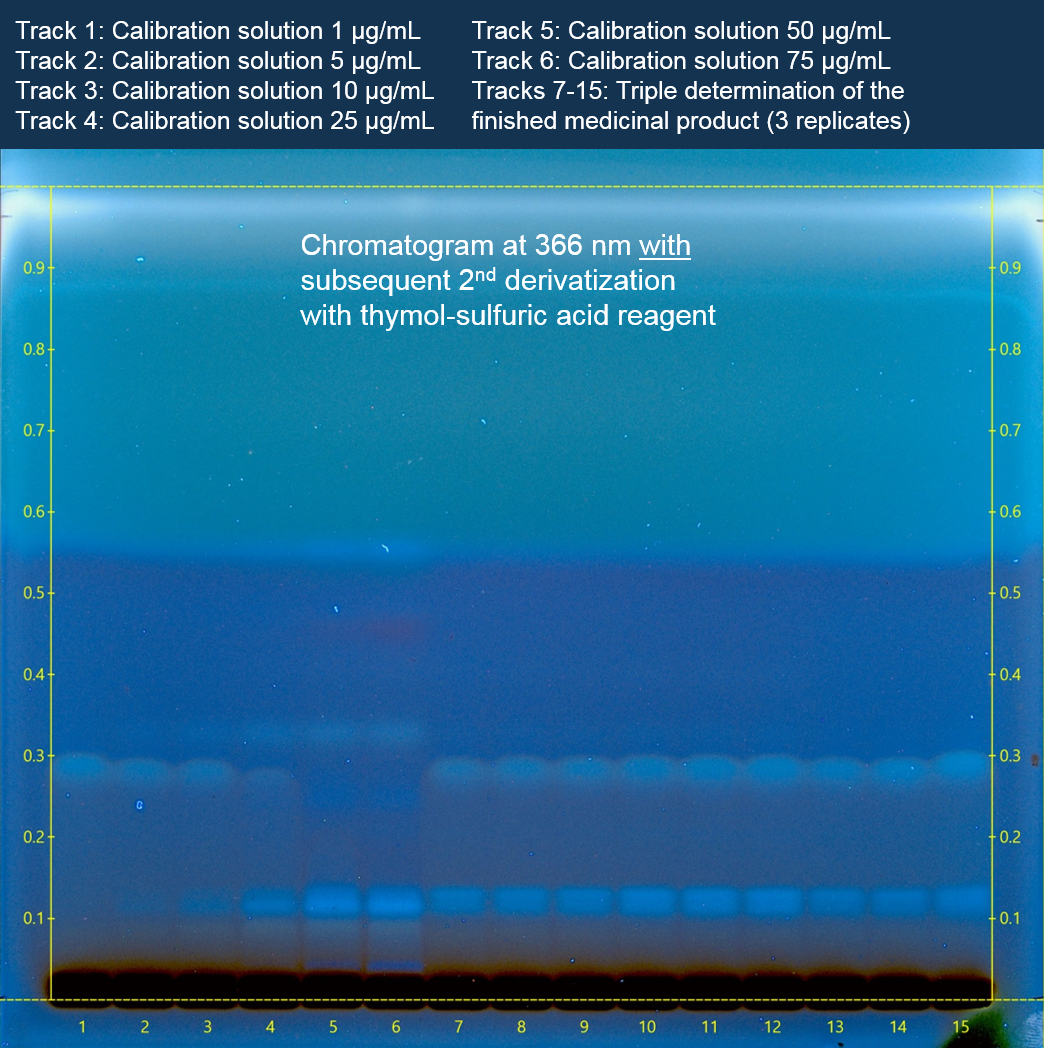


The calibration solutions contain all 7 GDPs in the respective concentrations mentioned in tracks 1-6, as well as 50 mg/mL glucose and 0.75 mg/mL OPD. 1 mL of the finished medicinal product also contained 0.75 mg/mL OPD (tracks 7-9).

**Statistical Analysis**

Linearity

- Linear regression is represented as y = ax + c

y- peak area of analyte (AUC)

a- slope

x- concentration of analyte (µg/mL)

c- y-axis intercept

- Polynomial regression is represented as y = ax^2^ + bx + c

y- peak area of analyte (AUC)

a- width and orientation

x- concentration of analyte (µg/mL)

b- shift in the relevant quadrant; vertex of the graph does not go through zero

c- y-axis intercept

Accuracy

Accuracy is determined as the mean value of three measurements at 3 specific concentrations divided by the true (spiked) value x 100%.

Mean(X̅) [2]


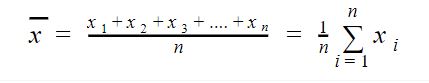


X- Measured value of the sample

n- number of samples

Precision

The precision is determined as the mean value of three measurements at 3 specific concentrations each. The precision is expressed as % RSD (= coefficient of variation). This is determined as follows:

Precision (expressed as coefficient of variation) = (s/ X̅) x 100 %

Standard deviation of a sample (s) [3]


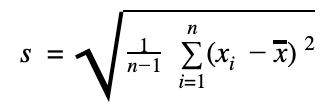


n- number of samples

X- Measured value of the sample

X̅- (mean) of the sample

Recovery

The mean recovery of three experiments for each concentration level was determined and expressed as: (GDP concentration-GDP concentration of the unspiked sample)/ added GDP concentration×100%.

**Reference** **List**

1. Council of Europe. European Pharmacopoeia 10.0. Glucose. Strasbourg: European Directorate for the Quality of Medicines; 2019. p. 2758-9.
2. scribbr. Arithmetisches Mittel verstehen und berechnen 2021 [Available from: https://www.scribbr.de/statistik/arithmetisches-mittel].
3. scribbr. Die Standardabweichung verstehen und berechnen 2021 [Available from: https://www.scribbr.de/statistik/standardabweichung].
